# Reshaping the Battlefield: Reprogramming the Melanoma Tumour Microenvironment (TME) by Anti-CTLA-4, Anti-PD-1, and Anti-PD-L1 Monotherapy and Combination Therapy: A Systematic Review and Meta-Analysis of Preclinical and Clinical Evidence

**DOI:** 10.3390/cells15131182

**Published:** 2026-06-29

**Authors:** Vasileios Alexandros Karakousis, Stylianos Mantalovas, Vasiliki Christina Karakousi, Ioannis S. Vizirianakis, Theodora Papamitsou, Leonidas Pavlidis, Christophoros S. Kosmidis

**Affiliations:** 1 School of Medicine, Faculty of Health Sciences, Aristotle University of Thessaloniki, 54124 Thessaloniki, Greece; steliosmantalobas@yahoo.gr (S.M.); kosmidisc@auth.gr (C.S.K.); 2 Laboratory of Histology-Embryology, School of Medicine, Faculty of Health Sciences, Aristotle University of Thessaloniki, 54124 Thessaloniki, Greece; thpapami@auth.gr (T.P.); 3 Department of Plastic Surgery, School of Medicine, Faculty of Health Sciences, Aristotle University of Thessaloniki, Papageorgiou General Hospital, 56403 Thessaloniki, Greece; pavlidisl@auth.gr (L.P.); 4 School of Pharmacy, Faculty of Health Sciences, Aristotle University of Thessaloniki, 54124 Thessaloniki, Greece; chriskarakousi@gmail.com (V.C.K.); 5 Laboratory of Pharmacology, School of Pharmacy, Faculty of Health Sciences, Aristotle University of Thessaloniki, 54124 Thessaloniki, Greece; ivizir@pharm.auth.gr (I.S.V.); 6 Department of Health Sciences, School of Life and Health Sciences, University of Nicosia, 1700 Nicosia, Cyprus; 7 Department of Surgery, European Interbalkan Medical Center, 57001 Thessaloniki, Greece

**Keywords:** melanoma, tumour microenvironment, immune checkpoint inhibitors, CTLA-4, PD-1, PD-L1, systematic review, meta-analysis, B16F10, biomarkers

## Abstract

**Highlights:**

**What are the main findings?**

- Immune checkpoint blockade consistently reprograms the melanoma tumour microenvironment toward an immune-activated, pro-inflammatory state, with robust increases in CD8^+^ T-cell infiltration demonstrated across preclinical B16F10 models and clinical cohorts.
- In the preclinical B16F10 literature, anti-PD-L1-containing regimens amplified terminal effector functions to a greater degree than anti-CTLA-4 monotherapy, producing larger effect sizes for IFN-γ, the CD8^+^/Treg ratio, and apoptosis, a differential not observed with anti-PD-1 or anti-CTLA-4 alone.

**What are the implications of the main findings?**

- The conserved CD8^+^ T-cell infiltration across species establishes a canonical mechanism of checkpoint inhibitor efficacy, while the divergent PD-L1 dynamics between mice and humans expose critical translational gaps that should be considered when extrapolating preclinical to clinical data.
- These findings provide a quantitative framework to guide rational combination design, inform the development of sequential or dual-targeting strategies that address compensatory immune checkpoints, and underscore the urgent need for harmonized TME biomarker reporting in immuno-oncology research to facilitate cross-study comparisons and accelerate clinical translation, ultimately shaping a roadmap for precision immunotherapy that bridges preclinical discovery with patient care.

**Abstract:**

Immune checkpoint inhibitors (ICIs), comprising anti-cytotoxic T-lymphocyte-associated protein 4 (CTLA-4), anti-programmed cell death protein 1 (PD-1), and anti-programmed death-ligand 1 (PD-L1), have transformed melanoma therapy, yet the tumour microenvironment (TME), the pivotal biological interface where therapeutic efficacy, resistance, and toxicity are determined, remains incompletely characterized. This dual systematic review and meta-analysis (PROSPERO: CRD420261374242) followed PRISMA 2020 and included 58 preclinical (B16F10/C57BL/6; 46 quantitative) and 44 clinical studies (19 quantitative) to calculate pooled standardized mean differences (SMDs) for six intratumoral TME parameters. Checkpoint blockade consistently shifted the TME toward an immune-activated state, an effect that remained robust in sensitivity analyses despite substantial heterogeneity (I-squared heterogeneity statistic (I^2^) = 68–88%). Preclinically, ICIs significantly increased CD8^+^ T-cell infiltration (SMD = 1.45, *p* < 0.001), interferon-gamma (IFN-γ) (SMD = 1.78, *p* < 0.001), CD8/regulatory T-cell (Treg) ratio (SMD = 0.91, *p* = 0.005), and apoptosis (SMD = 3.54, *p* < 0.001) and reduced PD-L1 (SMD = −0.88, *p* = 0.004) and Ki-67 (SMD = −1.43, *p* = 0.028). Clinically, CD8^+^ infiltration and PD-L1 both increased (SMD = 0.72, *p* < 0.001; SMD = 0.67, *p* = 0.001), contrasting with the preclinical PD-L1 decrease. Meta-regression demonstrated superior anti-PD-L1 efficacy over CTLA-4 for effector parameters: IFN-γ +3.59 (*p* = 0.009), CD8/Treg +10.69 (*p* = 0.003), apoptosis +9.76 (*p* = 0.004), and Ki-67 −6.28 (*p* = 0.040). These findings establish the TME as a critical determinant of ICI outcomes, indicate that PD-L1 amplifies effector functions in the B16F10 model, and highlight translational gaps in TME reprogramming.

## 1. Introduction

### 1.1. From Melanocyte to Malignancy: Epidemiology and Molecular Pathogenesis of Melanoma

The term **melanoma** derives from the Greek μέλας (melas, “black”) and -ωμα (-oma, “tumour”), reflecting the dark pigmentation of most lesions. **Cutaneous melanoma (CM)** is the most lethal form of skin cancer, accounting for over 75–80% of skin cancer mortality despite representing only 1–4% of all skin cancer cases [1,2]. Its global incidence has risen dramatically, with ~325,000 new cases and 57,000 deaths in 2020 [1]; in the United States, it is the fifth most common cancer, with incidence surging ~320% from 1975 to 2018 [3], while the lifetime risk for White individuals approaches 1 in 38, with a 1.6-fold male predominance [4]. Intermittent, intense ultraviolet (UV) radiation, especially blistering childhood sunburns, confers an approximately 2-fold relative risk [4,5]; host risk factors include fair skin, red hair, high nevus counts (>100, ~7-fold risk), atypical/dysplastic nevi, and personal or family history of melanoma [1,2], with familial cases (~10%) linked to germline mutations in cyclin-dependent kinase inhibitor 2A (CDKN2A) (up to 40% of hereditary cases) and BRCA1 associated protein 1 (BAP1) [2].

Melanoma arises from malignant transformation of melanocytes, with aberrant mitogen-activated protein kinase **(MAPK) pathway** activation in up to 90% of cases via mutually exclusive v-Raf murine sarcoma viral oncogene homolog B **(BRAF)** (37–60%, predominantly p.V600E) and neuroblastoma rat sarcoma virus oncogene homolog **(NRAS)** (15–30%) mutations that drive uncontrolled proliferation [2,3]. Subsequent inactivation of tumour suppressors, CDKN2A, tumour protein p53 (TP53), phosphatase and tensin homolog (PTEN) (lost in 10–30%), and neurofibromin 1 (NF1) (10–15%) enables progression from benign nevi to invasive disease [1,2,6]. Melanoma initially expands horizontally (radial growth phase), where it is curable by excision; vertical invasion (vertical growth phase) confers metastatic potential [1]. **Breslow thickness** is the strongest prognostic factor and the cornerstone of the American Joint Committee on Cancer **(AJCC) 8th edition staging system** [1,4].

Clinically, melanoma is suspected in any changing pigmented lesion and is classically evaluated using the **ABCDE criteria:** Asymmetry, Border irregularity, Color variegation, Diameter > 6 mm, and Evolution [1]. Diagnosis is aided by dermoscopy (increasing sensitivity up to 18%) [5], confirmed by excisional biopsy with assessment of Breslow thickness, ulceration, mitotic rate, and margin status [1] and supported by a panel of immunohistochemical markers including S100, human melanoma black 45 (HMB45) and melanoma antigen recognized by T cells 1 (Melan-A/MART1), SRY-related HMG-box 10 (SOX10) and microphthalmia-associated transcription factor (MITF), and PReferentially Expressed Antigen in Melanoma (PRAME) [2]. Melanoma is classified into four major clinicopathological subtypes: superficial spreading melanoma (~70%), nodular melanoma (~15%), lentigo maligna melanoma (~10%), and acral lentiginous melanoma (~2–5%), with additional rarer variants including desmoplastic and nevoid melanoma [1,5].

Staging according to the AJCC 8th edition integrates Breslow depth, ulceration (T category), nodal involvement (N category), and distant metastasis (M category, with serum lactate dehydrogenase (LDH) as a key modifier) [1,3,4]. Five-year survival rates range from >99% for stage IA disease to ~35% for stage IV melanoma, though outcomes for advanced disease have improved dramatically with modern systemic therapies [1,3]. Prognostic factors beyond staging include older age, male sex, head/neck location, ulceration, and elevated mitotic rate [1,2].

The therapeutic landscape for melanoma has been revolutionized by **immune checkpoint inhibitors (ICIs).** For resectable high-risk melanoma (stages IIB–IIC and III), adjuvant anti-programmed cell death protein 1 (PD-1) therapy (nivolumab or pembrolizumab) for one year significantly reduces the risk of recurrence and improves distant metastasis-free survival [1]. In the neoadjuvant setting, administration of ICIs prior to surgery, often with combination regimens such as nivolumab plus ipilimumab, induces major pathological responses and is associated with favorable long-term outcomes [1]. For unresectable or metastatic (stage IV) disease, frontline immunotherapy with anti-PD-1 monotherapy or dual checkpoint blockade (anti-PD-1 plus anti-cytotoxic T-lymphocyte-associated protein 4 [CTLA-4]) yields durable responses, while targeted therapy with BRAF plus MEK (mitogen-activated extracellular signal-regulated kinase) inhibitors is the standard for BRAF-mutant tumours [3]. Novel strategies, including lymphocyte activation gene 3 (LAG-3) inhibitors (relatlimab) in combination with nivolumab, personalized neoantigen vaccines, and adoptive cell transfer with tumour-infiltrating lymphocytes (TILs), are expanding the therapeutic armamentarium [3]. Despite these advances, primary and acquired resistances remain major clinical challenges, underscoring the need for a comprehensive, quantitative synthesis of tumour microenvironment **(TME)** reprogramming by distinct checkpoint inhibitor classes. A clearer understanding of the signalling pathways triggered by each checkpoint is therefore indispensable for identifying the molecular determinants of sensitivity and resistance, and for prioritising rational combination therapies.

### 1.2. A Dual Systematic and Meta-Analytic Cartography of TME Reprogramming

Beyond direct immune checkpoint blockade, effective immunotherapy fundamentally reprograms the melanoma TME from an immunosuppressive **(“cold”)** state toward an inflamed, T-cell-infiltrated **(“hot”)** state. This reprogramming involves coordinated changes in cellular composition, cytokine milieu, vascular architecture, and metabolic landscape. Preclinically, the B16F10 syngeneic model has been instrumental in dissecting these TME alterations; however, species-specific differences in adaptive resistance mechanisms, such as divergent PD-L1 dynamics, underscore the need for parallel quantitative synthesis across murine and human studies. We hypothesized that anti-CTLA-4, anti-PD-1, and anti-PD-L1 therapies would produce distinct and quantifiable TME signatures and that preclinical and clinical evidence would show both conserved and divergent remodeling patterns. The present dual systematic review and meta-analysis therefore aims to comprehensively map the conserved and divergent axes of TME remodeling induced by distinct immune checkpoint inhibitor classes, providing a quantitative framework to guide biomarker development and rational combination strategies in melanoma. As detailed below, the synthesis reveals a conserved CD8^+^ T-cell infiltration across species, a critical divergence in PD-L1 dynamics, and superior effector-function amplification by anti-PD-L1-containing regimens, thereby establishing a quantitative foundation for next-generation combination strategies.

This dual systematic review and meta-analysis was prospectively registered with the International Prospective Register of Systematic Reviews **(PROSPERO; registration number CRD420261374242)**. The full protocol, including pre-specified Population, Intervention, Comparator, Outcome, Timing, Setting **(PICOTS)** criteria, search strategies, eligibility criteria, and analytical plans for both preclinical and clinical arms, is publicly available on the PROSPERO website and is also provided as Supplementary File S1. No substantive deviations from the registered protocol were made during the conduct of this review.

### 1.3. Molecular Mechanisms of CTLA-4, PD-1, and PD-L1 Immune Checkpoint Blockade

The clinical application of ICIs in melanoma began with the approval of the anti-CTLA-4 antibody **ipilimumab** (immunoglobulin G1 [IgG1]) by the U.S. Food and Drug Administration (FDA) in 2011 for unresectable or metastatic melanoma, based on a significant overall survival benefit over a gp100 (glycoprotein 100) vaccine alone (median 10.1 vs. 6.4 months) [7]. CTLA-4 is upregulated on T cells shortly after activation and competes with the co-stimulatory receptor cluster of differentiation 28 (CD28) for binding to B7 ligands cluster of differentiation 80/cluster of differentiation 86 (CD80/CD86) on antigen-presenting cells (APCs), raising the threshold for T-cell priming and maintaining peripheral tolerance [8,9]. Ipilimumab blocks this interaction, thereby enhancing CD28-mediated co-stimulation, increasing interleukin (IL)-2 production, and promoting the expansion and diversification of effector T-cell repertoires within secondary lymphoid organs [7]. Additionally, the IgG1 fragment crystallizable (Fc) domain of ipilimumab may contribute to antitumour efficacy by engaging Fc gamma receptors (FcγRs) on innate immune cells, leading to antibody-dependent cellular cytotoxicity (ADCC) and depletion of intratumoral regulatory T cells (Tregs) [8,10].

In contrast, the PD-1 pathway operates predominantly within peripheral tissues and the tumour microenvironment (TME). PD-1 is expressed on activated T cells, B cells, and natural killer (NK) cells, while its ligands programmed cell death ligand 1 (PD-L1) and programmed cell death ligand 2 (PD-L2) are broadly expressed on tumour cells, stromal cells, and immune infiltrates [10]. Engagement of PD-1 by PD-L1 recruits the Src homology 2 domain-containing phosphatase 2 (SHP-2), which dephosphorylates proximal T-cell receptor (TCR) signaling molecules, thereby attenuating T-cell activation and driving a state of exhaustion characterized by reduced cytokine production and cytotoxic capacity [7,11]. This exhaustion is further reinforced by immunosuppressive metabolites within the TME, such as lactate and kynurenine, and by the physical barrier imposed by increased extracellular matrix stiffness [11]. The anti-PD-1 antibodies **nivolumab** and **pembrolizumab** (both immunoglobulin G4 (IgG4), FDA-approved in 2014) disrupt this inhibitory axis, reinvigorating exhausted cluster of differentiation 8^+^ (CD8^+^) T cells and restoring effector functions such as interferon γ (IFN-γ) secretion, granzyme B release, and tumour cell killing [10,12]. Notably, clinical response to PD-1 blockade is associated not only with the reinvigoration of pre-existing intratumoral exhausted T cells but also with the systemic recruitment of novel T-cell clonotypes from the peripheral blood, a process termed “clonal replacement”, as well as the expansion of C-X-C motif chemokine ligand 13 (CXCL13)^+^ T-cell subsets and the presence of mature tertiary lymphoid structures (TLSs) within the TME [11,13]. Anti-PD-L1 antibodies (e.g., **atezolizumab**, **durvalumab**) target the ligand rather than the receptor, achieving a similar functional outcome by preventing PD-L1 from engaging PD-1 and potentially modulating PD-L1-expressing myeloid cells within the TME [8]. In cutaneous melanoma, skin-resident immune populations, particularly tissue-resident memory T cells and Langerhans cells, also shape the local immune landscape and influence ICI responsiveness [11].

**Figure 1:**
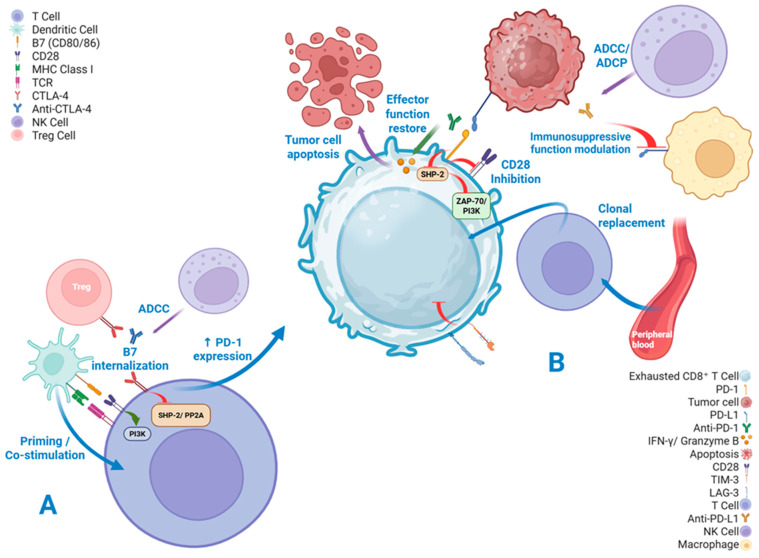
**Molecular mechanisms of immune checkpoint blockade by anti-cytotoxic T-lymphocyte-associated protein 4 (CTLA-4) (ipilimumab, tremelimumab), anti-programmed cell death protein 1 (PD-1) (nivolumab, pembrolizumab), and anti-programmed death-ligand 1 (PD-L1) (atezolizumab, durvalumab, avelumab) antibodies.** (**A**) **Anti-CTLA-4 pathway**. Left: An antigen-presenting dendritic cell (pale green) displays major histocompatibility complex class I (MHC-I) (green) and B7 cluster of differentiation 80/cluster of differentiation 86 (CD80/CD86) (orange) on its surface. These engage T-cell receptor (TCR) (pink) and cluster of differentiation 28 (CD28) (blue), respectively, on a naïve/effector T cell (purple). This dual interaction delivers Signal 1 (antigen presentation) and Signal 2 (co-stimulation), leading to intracellular activation of phosphoinositide 3-kinase (PI3K)–protein kinase B (AKT) and downstream interleukin-2 (IL-2) production and T-cell proliferation (blue arrow). Cytotoxic T-lymphocyte-associated protein 4 (CTLA-4) (red), also expressed on the T cell, competes with CD28 for B7 and recruits Src homology 2 domain-containing phosphatase 2 (SHP-2)/protein phosphatase 2A (PP2A) (yellow inhibitory oval) to dampen TCR/CD28 signaling. CTLA-4 also promotes B7 internalization. The anti-CTLA-4 antibody ipilimumab (blue) blocks CTLA-4–B7 binding, restoring co-stimulation. Additionally, ipilimumab (immunoglobulin G1, IgG1) engages Fc gamma receptors (FcγRs) on natural killer (NK) cells (pale purple) to mediate antibody-dependent cellular cytotoxicity (ADCC) against regulatory T cells (Tregs) (pale red) that highly express CTLA-4, leading to Treg depletion. CTLA-4 blockade also upregulates programmed cell death protein 1 (PD-1) expression (blue arrow) on T cells, linking to the PD-1 pathway. (**B**) **Anti-PD-1 and anti-PD-L1 pathways.** An exhausted cluster of differentiation 8 (CD8^+^) T cell (pale turquoise) expresses PD-1 (yellow), T-cell immunoglobulin and mucin domain-containing protein 3 (TIM-3) (orange), and lymphocyte activation gene 3 (LAG-3) (light blue) on its surface. A tumour cell (dark brown) expresses PD-L1 (blue). PD-1–PD-L1 engagement recruits SHP-2 (yellow oval), which inhibits proximal zeta chain-associated protein kinase 70 (ZAP-70)/PI3K signaling (red inhibitory arrow) and also directly inhibits CD28 co-stimulation (red inhibitory arrow), driving T-cell exhaustion. The anti-PD-1 antibodies nivolumab/pembrolizumab (green) block PD-1–PD-L1 binding, restoring effector functions including interferon γ (IFN-γ) and granzyme B (yellow) production (green arrow). Restored cytotoxicity leads to tumour cell apoptosis (fragmented cell, purple arrow). Systemic clonal replacement from peripheral blood (blood vessel) supplies novel T-cell clonotypes that contribute to the anti-tumour response. In the anti-PD-L1 pathway, a tumour cell expresses PD-L1 on its surface. The anti-PD-L1 antibody (golden) binds directly to PD-L1, preventing its interaction with PD-1 on the exhausted CD8^+^ T cell. This blockade restores T cell effector function (IFN-γ, granzyme B) and leads to tumour cell apoptosis. Additionally, anti-PD-L1 antibodies of the IgG1 subclass (e.g., avelumab) engage FcγRs on NK cells (pale purple) to mediate ADCC/antibody-dependent cellular phagocytosis (ADCP) against PD-L1^+^ tumour cells. Furthermore, PD-L1 is also expressed on tumour-associated macrophages (Mφ, light yellow). Anti-PD-L1 blockade of this ligand reverses immunosuppressive myeloid cell modulation, thereby alleviating a key axis of immune evasion within the tumour microenvironment. Together, these mechanisms highlight the dual action of anti-PD-L1 therapy: direct restoration of T-cell cytotoxicity and remodeling of the immunosuppressive myeloid compartment. Created with BioRender.com [7,8,9,10,11,12,13].

Dual checkpoint blockade with anti-CTLA-4 plus anti-PD-1 (ipilimumab plus nivolumab, FDA-approved 2015) yields superior response rates and overall survival compared with either monotherapy, as demonstrated in the **CheckMate 067 trial**, where 3-year overall survival reached 58% for the combination versus 52% for nivolumab and 34% for ipilimumab alone, albeit with increased immune-related adverse events **(irAEs)**, underscoring the complementary and potentially synergistic nature of these two pathways [7,12]. Additional immune checkpoints, including LAG-3 (relatlimab approved 2022 in combination with nivolumab), as well as investigational strategies such as personalized messenger RNA (mRNA) neoantigen vaccines, oncolytic viral therapies (e.g., talimogene laherparepvec [T-VEC]), and adoptive cell transfer with TILs, are expanding the therapeutic armamentarium but lie beyond the scope of the present systematic review and meta-analysis [7,12]. The molecular cascades underlying CTLA-4, PD-1, and PD-L1 checkpoint blockade, including their intracellular signaling nodes, Fc-mediated effector functions, and cross-talk, are illustrated in Figure 1.

### 1.4. The B16F10 Syngeneic Model: A Clinically Anchored Platform for Dissecting Melanoma TME Reprogramming

Preclinically, the **B16F10** murine melanoma model, syngeneic to immunocompetent **C57BL/6** mice, is the most widely employed system for evaluating ICI efficacy and TME remodeling. Originally isolated for its high metastatic potential, B16F10 reliably disseminates via both lymphatic and hematogenous routes, recapitulating key features of clinical melanoma progression [14]. The model is notoriously difficult to treat, characterized by low major histocompatibility complex (MHC) class I expression and poor intrinsic immunogenicity, which together limit direct translation of certain adaptive resistance mechanisms (e.g., PD-L1 dynamics) [15]. Nevertheless, this stringency renders B16F10 a conservative and clinically relevant platform for dissecting conserved axes of immune activation and for stress-testing novel immunotherapeutic strategies.

## 2. Materials and Methods

### 2.1. Literature Search and Screening

#### 2.1.1. Preclinical Arm

A comprehensive systematic search was executed across four major electronic databases, **PubMed (*n* = 528), Scopus (*n* = 441), Web of Science (*n* = 480),** and the **Cochrane Library (*n* = 529),** from their respective dates of inception through 20 January 2026. The search syntax was meticulously constructed to intersect four conceptual domains: (i) the experimental model (e.g., “melanoma,” “B16F10,” “syngeneic,” “murine”); (ii) the immunotherapeutic intervention, encompassing anti-CTLA-4 agents (ipilimumab, tremelimumab), anti-PD-1 agents (nivolumab, pembrolizumab), and anti-PD-L1 agents (atezolizumab, avelumab, durvalumab), as well as broader terms such as “immune checkpoint inhibitor;” (iii) descriptors of the tumour microenvironment (e.g., “tumour infiltrating lymphocytes,” “CD8,” “Forkhead box P3 (FoxP3),” “cytokine,” “chemokine,” “stroma”); and (iv) the preclinical study design (e.g., “in vivo,” “animal model,” “mouse”). No restrictions were placed on publication date at the search stage. Searches were confined to English-language literature. The complete, database-specific search strings are publicly available in the PROSPERO registry and are also provided as Supplementary Table S1.

**Figure 2:**
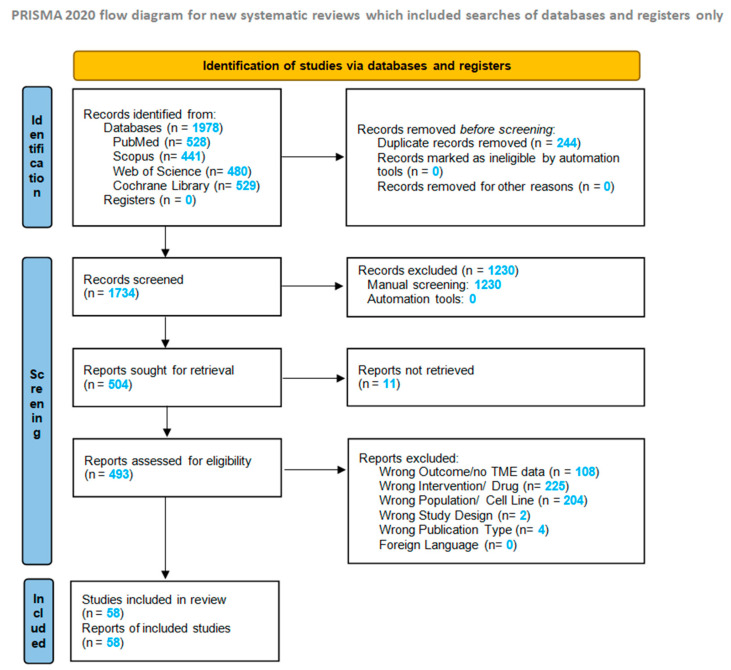
**Preferred Reporting Items for Systematic Reviews and Meta-analyses (PRISMA) 2020 flow diagram for the preclinical arm of the systematic review and meta-analysis.** This flowchart depicts the selection process for preclinical studies evaluating the tumour microenvironment (TME) in cutaneous B16F10 murine melanoma models treated with anti-CTLA-4, anti-PD-1 and/or anti-PD-L1 immunotherapy. ***Note:*** *Some studies were excluded for more than one reason; therefore, the sum of exclusion reasons exceeds the total number of excluded reports.* **Source:** Page, M.J.; et al. *BMJ* **2021**, *372*, n71. https://doi.org/10.1136/bmj.n71 [16]. This work is licensed under CC BY 4.0. To view a copy of this license, visit https://creativecommons.org/licenses/by/4.0/.

The entire selection workflow adhered strictly to the Preferred Reporting Items for Systematic Reviews and Meta-Analyses **(PRISMA) 2020 statement** [16]; the completed PRISMA 2020 checklist is provided as Supplementary File S2. The PRISMA flow diagram detailing the preclinical study selection is presented in Figure 2. Following the removal of **244** duplicate records, **1734** unique citations were advanced to the title and abstract screening phase. Working within the Rayyan systematic review platform (https://rayyan.ai), two independent reviewers, blinded to one another’s assessments, evaluated each record against the pre-specified eligibility criteria. Discrepancies in this initial stage were resolved through consensus-driven discussion; when unanimity proved elusive, a third adjudicator rendered the decisive verdict. All exclusion decisions were systematically documented and cross-checked across reviewers, ensuring full transparency and adherence to PRISMA 2020 standards. This rigorous triage ensured that only studies conforming to the pre-defined model, intervention, and read-out criteria advanced to synthesis, thereby safeguarding the internal validity of the pooled estimates.

A total of **1230** records were excluded at the title and abstract level. The full texts of the remaining **504** reports were methodically procured for detailed appraisal; full text retrieval failed for 11 reports. Each of the **493** thoroughly evaluated reports was measured against the study inclusion criteria. The principal rationales for exclusion during the full text review were: inappropriate outcome metrics or absence of quantifiable tumour microenvironment data **(*n* = 108),** utilization of an ineligible intervention or pharmaceutical agent **(*n* = 225),** employment of an unsuitable cell line or animal model **(*n* = 204),** inapt study architecture **(*n* = 2),** and publication of a non-eligible article type **(*n* = 4).** Several reports were excluded on multiple grounds; consequently, the aggregate of individual exclusion reasons exceeds the total number of excluded reports. Ultimately, **58** studies fulfilled all eligibility requirements and were enshrined in the qualitative systematic review. Of these, **46** contributed extractable quantitative data (mean and standard deviation) and were advanced to the meta-analytic synthesis.

##### Rationale for Stringent Preclinical Model Standardization and Inclusion Criteria

The preclinical immuno-oncology literature is characterized by extraordinary methodological heterogeneity, encompassing diverse syngeneic mouse strains (e.g., C57BL/6, BALB/c); melanoma cell lines with variable immunogenicity (e.g., B16F10, B16-OVA (ovalbumin-expressing), YUMM, RET); and an array of drug dosing schedules and analytical endpoints, as well as a variety of tumour inoculation methods (e.g., intradermal, intravenous) and drug administration routes (e.g., intravenous, intratumoral). While this breadth reflects the exploratory nature of the field, it introduces substantial experimental noise that can obscure the true biological signal attributable to a specific therapeutic intervention when studies are aggregated in a systematic review. Without rigorous harmonization, meta-analysis risks comparing fundamentally different biological contexts, equating an immune response against a highly immunogenic engineered antigen (OVA) to a response against endogenous melanoma antigens, rather than isolating the effect of immune checkpoint blockade itself. Consequently, to address our specific research question concerning the differential reprogramming of the tumour microenvironment (TME) by anti-CTLA-4 and anti-PD-1/PD-L1 therapies, a deliberate and restrictive approach to study inclusion is not merely a methodological preference but an epistemological necessity.

We therefore applied an a priori set of stringent inclusion criteria designed to create a biologically and immunologically standardized cohort. All included studies were restricted to **immunocompetent C57BL/6 mice** bearing **unmodified, parental B16F10 cutaneous** melanoma tumours **implanted subcutaneously** and receiving **intraperitoneal administration** of the therapeutic agent. This specific syngeneic pairing, together with the requirement for subcutaneous tumour inoculation and intraperitoneal drug delivery, represents the most widely utilized and translationally challenging model in the field, characterized by its relatively low mutational burden and poor intrinsic immunogenicity, thereby providing a conservative and clinically relevant baseline for assessing TME modulation. Critically, we excluded all antigen-enhanced variants (e.g., B16-OVA) to prevent the artificial inflation of immunogenicity, as well as studies employing intradermal or intravenous tumour inoculation or intravenous or intratumoral drug delivery, and mandated that all TME data be derived exclusively from **intratumoral analysis.** The requirement for intratumoral quantification, as opposed to peripheral blood or splenic assessment, ensures anatomical precision, allowing us to interrogate the actual battlefield of the tumour-host interface where spatial architecture, cellular composition, and stromal remodeling dictate therapeutic success or failure. Furthermore, we enforced a strict analytical discipline whereby a study was included only if it contained a dedicated, extractable experimental leg meeting all model, intervention, and tissue-source criteria, rejecting any data that required pooling with ineligible comparators.

This methodological framework entails a conscious trade-off between generalizability and internal validity. By sacrificing breadth across varied murine models and cell lines, we constructed a curated, high-fidelity corpus of comparable experiments. The resulting reduction in exogenous experimental variance substantially enhances the statistical power and biological interpretability of our pooled estimates. The heterogeneity that remains within this standardized cohort, for instance, variability in the magnitude of CD8+ T-cell infiltration or the CD8/Treg ratio, can therefore be attributed with greater confidence to true biological variation in treatment response, tumour heterogeneity, or subtle differences in experimental design rather than to fundamental incompatibilities in model systems. This approach does not limit the review’s value; rather, it transforms the analysis from a diffuse summary of disparate findings into a focused, mechanistic interrogation of how checkpoint blockade remodels the TME within a specific, translationally anchored context. The resulting synthesis provides a clearer and more actionable foundation for identifying biomarkers of response and resistance and informing the rational design of next-generation combination strategies.

#### 2.1.2. Clinical Arm

A systematic literature search was performed in four electronic databases: **PubMed (*n* = 531), Scopus (*n* = 452), Web of Science (*n* = 585),** and **Cochrane Library (*n* = 542),** from inception to 20 January 2026. The search strategy combined terms related to the population (e.g., “melanoma”, “stage III/IV”); the intervention, encompassing anti-CTLA-4 agents (e.g., ipilimumab), anti-PD-1 agents (e.g., nivolumab), and anti-PD-L1 agents (e.g., atezolizumab); as well as broader terms such as “immune checkpoint inhibitor,” the tumour microenvironment (e.g., “tumour infiltrating lymphocytes”, “CD8”, “PD-L1”, “cytokine”, “chemokine”), and the study design (e.g., “clinical trial”, “cohort”, “biomarker study”). The search was restricted to English-language publications. No publication date restrictions were applied. The complete, database-specific search strings are publicly available in the PROSPERO registry and are also provided as Supplementary Table S1.

**Figure 3:**
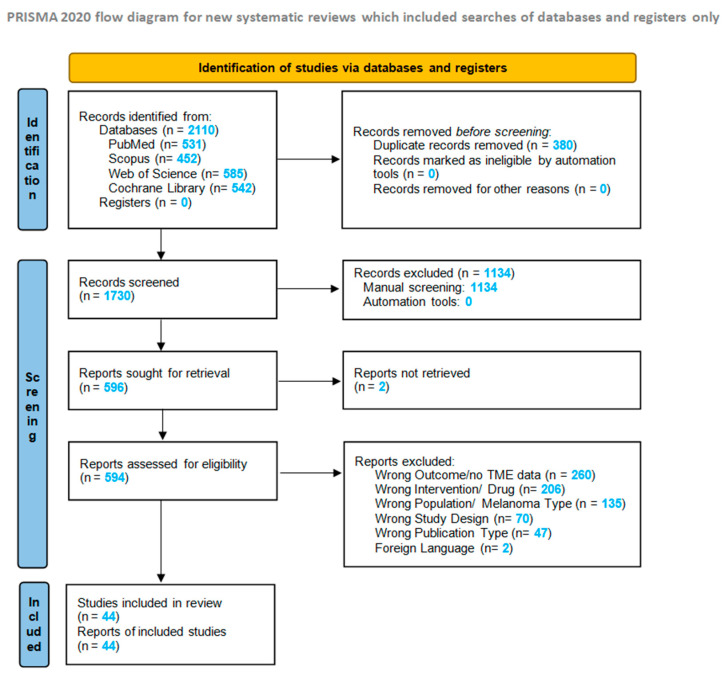
**PRISMA 2020 flow diagram for the clinical arm of the systematic review and meta-analysis.** This flowchart represents the selection process for studies evaluating the tumour microenvironment (TME) in cutaneous melanoma patients treated with anti-CTLA-4, anti-PD-1 and/or anti-PD-L1 immunotherapy. ***Note:*** *Some studies were excluded for more than one reason; therefore, the sum of exclusion reasons exceeds the total number of excluded reports.* **Source:** Page, M.J.; et al. *BMJ* **2021**, *372*, n71. https://doi.org/10.1136/bmj.n71 [16]. This work is licensed under CC BY 4.0. To view a copy of this license, visit https://creativecommons.org/licenses/by/4.0/.

The study selection process was conducted in accordance with the PRISMA 2020 guidelines [16]. The PRISMA flow diagram for the clinical arm is presented in Figure 3.

Two independent reviewers (blinded to each other’s decisions) screened the titles and abstracts of all records retrieved from the electronic databases using the Rayyan systematic review web application. Disagreements at the title/abstract stage were resolved by discussion; if no consensus was reached, a third reviewer made the final decision. The same two reviewers then assessed the full-text articles of potentially eligible studies against the pre-specified inclusion and exclusion criteria. Again, any discrepancies were resolved by consensus or consultation with a third reviewer. The search yielded **2110** records. After removing **380** duplicates, **1730** records were screened. Of these, **1134** were excluded based on title/abstract (manual screening). The remaining **596** reports were sought for retrieval; two could not be obtained. The full texts of the remaining **594** reports were assessed for eligibility. The reasons for exclusion were: wrong outcome/no TME data **(*n* = 260)**, wrong intervention/drug **(*n* = 206)**, wrong population/melanoma type **(*n* = 135)**, wrong study design **(*n* = 70),** wrong publication type **(*n* = 47)**, and foreign language **(*n* = 2)**. Some studies were excluded for more than one reason; therefore, the sum of exclusion reasons exceeds the total number of excluded reports. Ultimately, **44** studies met the eligibility criteria and were included in the systematic review. Of these, **19** provided extractable mean and standard deviation data and were included in the quantitative meta-analysis.

### 2.2. Selection and Prioritization of TME Parameters for Quantitative Synthesis

Following the exhaustive extraction of TME data from all eligible preclinical and clinical studies, a panel of three investigators undertook a systematic, consensus-driven appraisal to determine which parameters possessed sufficient statistical viability for meta-analytic pooling. The decision was governed entirely by the empirical landscape of the extracted data: parameters were prioritized according to the frequency with which studies reported extractable means and standard deviations, thereby ensuring that each pooled estimate was anchored in a robust evidentiary foundation. From the assembled corpus, six intratumoral endpoints emerged in the following hierarchical order of data richness: (1) **CD8^+^ T-cell infiltration**, (2) **CD8^+^/regulatory T-cell (Treg) ratio**, (3) **programmed death-ligand 1 (PD-L1) expression**, (4) **interferon-γ (IFN-γ) levels**, (5) **Ki-67 proliferation index**, and (6) **apoptosis**. This sequence was applied identically across the preclinical and clinical arms, furnishing a symmetrical framework for the comparative synthesis. The complete curated datasets, comprising every individual study’s mean, standard deviation, sample size, and drug-class assignment for each parameter, are archived as Supplementary Table S2 (preclinical arm, **99 data points,** and clinical arm, **44 data points**). A single data point was defined as the complete set of information required to compute one standardized mean difference for one comparison from one study: the number of subjects, the mean, and the standard deviation for each group, together with the comparator category that defined the grouping.

### 2.3. Completeness of Data Across Key TME Parameters

#### 2.3.1. Preclinical Arm

A methodical audit of the six pre-specified intratumoral parameters across the 46 included preclinical studies revealed a reporting landscape both richer and more granular than that observed in the clinical corpus, yet still marked by notable asymmetry, as the enumeration below makes clear. **CD8^+^ T cell infiltration** was by far the most comprehensively documented endpoint, with extractable quantitative data available in **39 studies (85%; 41 data points)**. This ubiquity reflects the central role of cytotoxic T lymphocyte abundance as the canonical readout of immunotherapeutic efficacy in syngeneic melanoma models. The **CD8/Treg ratio**, a functionally salient metric of the intratumoral effector to suppressor equilibrium, was extractable from **16 studies (35%; 17 data points)**, providing a moderately robust foundation for pooled analysis. **Interferon-γ (IFN-γ)**, the quintessential T helper 1 (Th1) type cytokine and harbinger of productive anti-tumour immunity, was quantifiable in **19 studies (41%; 21 data points)**, confirming that the reporting of this central cytokine was both abundant and sufficiently consistent to warrant quantitative pooling across studies.

Critically, the preclinical corpus furnished substantially more abundant and consistently reported data for the terminal effector parameters of proliferation and apoptosis than were available in the clinical literature. **Ki-67** immunohistochemistry, the gold standard surrogate for cellular proliferation, was extractable from **8 studies (17%; 9 data points)**, while **apoptosis**, assessed predominantly via terminal deoxynucleotidyl transferase dUTP nick end labeling (TUNEL) staining or cleaved caspase-3 immunoreactivity, was reported in **6 studies (13%; 7 data points)**. Although these denominators remain modest relative to CD8^+^ infiltration, they nevertheless constitute a meaningful evidentiary base that permits quantitative synthesis of tumour cell fate dynamics following checkpoint blockade, a dimension almost entirely absent from the clinical meta-analysis. **PD-L1** expression on tumour or immune cells, in contrast, was the most sparsely reported parameter, with extractable data emerging from only **3 studies (7%; 4 data points)**.

The richer preclinical data for apoptosis and Ki-67 thus provide a crucial mechanistic counterweight to the clinical synthesis, offering direct experimental evidence for the terminal consequences of tumour microenvironment reprogramming, namely, the suppression of tumour cell proliferation and the induction of programmed cell death, which remains largely opaque in human studies.

#### 2.3.2. Clinical Arm

Among the six pre-specified intratumoral parameters, **CD8^+^ T-cell infiltration** was by far the most frequently reported in the clinical literature, appearing in **17 studies (89%)** and yielding **19 data points**. Although this dataset was considerably smaller than its preclinical counterpart, it nonetheless provided a sufficiently robust foundation for meta-analytic pooling. A notable reversal of the preclinical pattern was observed for **PD-L1 expression**, which was documented in **9 clinical studies (47%; 10 data points)** and exceeded the clinical reporting of many other parameters; indeed, PD-L1 was the only endpoint for which the clinical contribution substantially outweighed the preclinical one. The **CD8/Treg ratio** occupied an intermediate position, with extractable data from **7 studies (37%; 8 data points)**, a modest but clinically informative tally.

In contrast, the remaining parameters were sparsely represented. **IFN-γ** was extractable from only **4 clinical studies (21%; 4 data points)**, reflecting that this cytokine, despite its central biological role, was reported in only a small minority of clinical studies, a pattern that constrained the statistical power available for its pooled analysis.

**Ki-67** proliferation data were available from just **2 studies (11%)**, yielding **3 data points**, a stark contrast to the more abundant preclinical data for this terminal effector parameter. **Apoptosis** was the most poorly documented endpoint, reported in **a single clinical study (5%)** as apoptotic debris count per high-power field **(1 data point)**, thereby precluding any quantitative synthesis in the clinical meta-analysis. Overall, the clinical arm furnished **45 data points** from **19 distinct studies**, an evidentiary base that, while valuable, was markedly leaner than the preclinical corpus and lacked the mechanistic depth for proliferation and apoptosis that the preclinical synthesis uniquely provided. This asymmetry underscores the complementary nature of the two arms: the clinical data illuminate the core immunological signals, while the preclinical data supply the mechanistic depth that human studies currently lack.

##### Assessment of Patient Overlap Across Included Studies

To avoid double-counting of patients in the meta-analysis, we systematically verified potential population overlap among the 19 included clinical studies. Each study was cross-checked by clinical trial registration number, enrolment period, treatment regimen, sample size, contributing institutions, and author groups. All studies were confirmed to have independent patient populations with no evidence of overlap.

### 2.4. Handling of Heterogeneous Units

#### 2.4.1. Preclinical Arm

A fundamental challenge in synthesizing intratumoral immune parameters across the 46 included preclinical studies was the profound heterogeneity in measurement scales and reporting conventions. CD8^+^ T cell infiltration, for instance, was variously expressed as cells per cubic millimeter, cells per milligram of tumour tissue, percentage of cluster of differentiation 45 (CD45^+^) leukocytes, percentage of cluster of differentiation 3 (CD3^+^) T lymphocytes, absolute counts per high-power microscopic field, or total cells per gram of tumour mass. The CD8/Treg ratio was derived from disparate parent populations, with Tregs reported as a fraction of cluster of differentiation 4 (CD4^+^) cells, CD45^+^ cells, or total viable cells. IFNγ concentrations were assessed by tissue lysates (picograms per milligram protein), flow cytometric intracellular staining (percentages), immunohistochemical intensity scores, and Western blotting (relative densitometry units). Ki-67 and apoptosis were quantified as percentages of positive cells, absolute counts per field, or normalized fold changes relative to control. Direct arithmetic pooling of raw means across such fundamentally incommensurable scales would be statistically indefensible. Consequently, all preclinical meta-analyses were conducted using the **standardized mean difference (SMD; Hedges’ g)**, which expresses the difference between the treatment and control groups in units of standard deviation. This **dimensionless metric** harmonizes the disparate measurement scales, enabling valid quantitative synthesis of the direction and magnitude of therapeutic effects across studies regardless of the original units. The use of SMD is a widely endorsed approach for meta-analyses of continuous outcomes afflicted by heterogeneous measurement conventions.

#### 2.4.2. Clinical Arm

An analogous, yet distinct, measurement heterogeneity characterized the clinical literature as well. For example, CD8^+^ T cell density was reported as cells/mm^2^, ×10^3^ cells/mm^2^, percentage of CD45^+^ cells, percentage of CD8^+^-positive area, or percentage of all cells. Similarly, PD-L1 expression was quantified using different scoring systems (immunohistochemistry (IHC) score, digital spatial profiling (DSP) score, melanoma PD-L1 expression score (MELscore), mRNA ratio, or log10% tumour cells), and IFNγ was measured as signature scores derived from RNA sequencing (RNA-seq) or mRNA expression, ΔZ scores, or antilog robust multi-array average (RMA) units. Direct pooling of raw means across such disparate scales would be invalid. Therefore, all analyses were performed using the SMD, which expresses the difference between two groups (e.g., responders versus non-responders, post-treatment versus pre-treatment, no relapse versus relapse) in units of standard deviation, thereby enabling meaningful aggregation across studies regardless of the original measurement scale. This approach is the standard of practice in evidence synthesis, explicitly endorsed by international methodological guidelines and extensively validated in meta-epidemiological research as the preferred metric when synthesising studies that assess the same underlying construct through incomparable instruments or scales.

### 2.5. Data Extraction from Graphical Sources

Since the majority of primary studies reported quantitative results only in graphical form (e.g., bar charts, scatter plots, line graphs) without accompanying numerical tables, a substantial number of means and standard deviations were obtained using the validated, open-source tool WebPlotDigitizer (version 4.6; Ankit Rohatgi, Pacifica, CA, USA). Extraction was performed independently by two reviewers, and any discrepancies were resolved through discussion with a third investigator. This approach is widely accepted in meta-analytic research and enabled the inclusion of data that would otherwise have been unavailable for quantitative synthesis.

### 2.6. Standard Deviation Estimation

#### 2.6.1. Preclinical Arm

For studies in which exact **standard deviations (SDs)** were reported directly in the text or tables, these values were extracted without modification, e.g., [17,18]. When data were presented as the **standard error of the mean (SEM)**, the SD was calculated using the established formula SD = SEM × √n, where n denotes the sample size per group, e.g., [19,20,21,22,23,24,25]. For studies that displayed data as box plots, the SD was estimated from the interquartile range using the **Wan method** [26], specifically, SD = **interquartile range (IQR)**/1.35, e.g., [27]. When only the minimum, mean, and maximum values were available from dumbbell plots or bar charts, the SD was estimated as (max − min)/4, a conservative approximation based on the properties of the normal distribution, e.g., [28,29,30,31,32,33,34,35,36]. For stacked bar charts, means were calculated as the difference between the top of each segment and the baseline, and error bars were interpreted according to the figure legend, typically as SEM, with subsequent conversion to SD [19]. In cases where raw individual data points were plotted, means and SDs were computed directly from the extracted values, e.g., [37,38,39,40,41]. For the CD8/Treg ratio, when the ratio was not directly reported but its constituent means and SDs were available, the ratio mean was computed as CD8/Treg, and the SD was derived via **error propagation**, assuming no correlation between the numerator and denominator, e.g., [19,20,23,29,32,33,42]. In a few instances, the CD8 mean and SD were derived from the product of two reported percentages (e.g., %CD3^+^ of CD45^+^ and %CD8^+^ of CD3^+^), with the SD propagated accordingly [43]. These methodical, transparent approaches ensured that all eligible studies, regardless of their original data presentation format, could be incorporated into the quantitative synthesis without compromising the integrity or robustness of the pooled estimates.

#### 2.6.2. Clinical Arm

Likewise, for studies reporting exact SD, these were extracted directly, e.g., [44,45,46]. When only the SEM was available, SD was calculated as SEM multiplied by the square root of the sample size [47,48]. For box-plot data, the Wan method [26] was applied [49], and for data presented on a logarithmic axis [50], the extracted values naturally reflect the log-normal distribution, yielding a larger SD that is appropriately handled by the SMD metric used in the meta-analysis. Where only the range or interquartile range was reported, the SD was estimated as range/4 [51,52] or IQR/1.35 [53]. These established methods ensured that all eligible studies could be included in the quantitative synthesis without compromising the robustness of the pooled estimates. Combined with the standardized analytical framework applied across both arms of the review, including algebraic conversions and geometric approximations, these estimation procedures formed a rigorous and transparent pipeline that rendered the heterogeneous clinical evidence amenable to quantitative pooling alongside the preclinical data.

### 2.7. Drug Regimens and Subgroup Analysis

#### 2.7.1. Preclinical Arm

The 46 included preclinical studies evaluated a diverse spectrum of immune checkpoint inhibitors, encompassing monoclonal antibodies **targeting CTLA-4 (clones** 9H10, 9D9), the **PD-1/PD-L1** axis (clones RMP1-14, 10F.9G2, BE0146), and their combinations. This experimental breadth exceeded that of the clinical literature, with five distinct therapeutic categories represented: **anti-CTLA-4 monotherapy**, **anti-PD-1 monotherapy**, **anti-PD-L1 monotherapy**, **anti-CTLA-4 + anti-PD-1 dual blockade**, and **anti-CTLA-4 + anti-PD-L1 dual blockade**. To facilitate a clinically meaningful and statistically robust synthesis, studies were stratified into these five categories for subgroup analysis. In instances where a study reported multiple eligible arms (e.g., both anti-CTLA-4 and anti-PD-1 monotherapy groups within the same publication), each arm was treated as an independent data point in the relevant subgroup. All meta-analyses were conducted both as an overall pooled estimate encompassing all drug classes and as subgroup analyses stratified by therapeutic category. This dual approach permitted an assessment of the global TME remodeling effects of checkpoint blockade while enabling a mechanistic dissection of the differential reprogramming elicited by distinct nodes of immune inhibition, whether targeting CTLA-4-mediated priming, PD-1/PD-L1-mediated effector exhaustion, or the synergistic engagement of both pathways.

#### 2.7.2. Clinical Arm

The clinical studies included in this meta-analysis evaluated immune checkpoint inhibitors targeting CTLA-4 (ipilimumab, tremelimumab), the PD-1/PD-L1 axis (pembrolizumab, nivolumab, toripalimab), or their combination. To enable clinically meaningful synthesis, studies were classified into three drug categories: **anti-CTLA-4 monotherapy**, **anti-PD-1 monotherapy**, and **anti-CTLA-4+anti-PD-1 combination therapy**. In one study [44], patients receiving anti-PD-1 monotherapy or anti-PD-1+anti-CTLA-4 combination were pooled, and individual patient data could not be separated. Given that the number of patients on monotherapy in this study was too small to drive the overall effect estimate, the entire cohort was classified under the combination subgroup. No clinical study evaluating anti-PD-L1 monotherapy or any anti-PD-L1-containing combination met the inclusion criteria for the quantitative meta-analysis; therefore, this drug class does not appear in the clinical subgroup analysis. All analyses were performed both as an overall pooled estimate across all drug classes and as subgroup analyses stratified by drug category to explore potential differential effects on TME parameters.

##### Comparators and Subgroup Analysis

In the clinical arm, all comparisons extracted from the included studies were collectively considered under the umbrella term **‘favorable vs. non-favorable outcome’**. Subsequently, subgroup analyses were performed according to three distinct comparator categories: (1) **‘responders vs. non-responders’**, which encompassed radiological response, ctDNA status [46], and pathological response [49]; (2) **‘post-treatment vs. pre-treatment’**, comprising paired longitudinal comparisons within the same patients; and (3) **‘no relapse vs. relapse’**, representing the unique time-to-event comparison from one study [54]. For each comparator category, the favorable outcome was defined as follows: responders (including radiological response, ctDNA negativity, and pathological response) versus non-responders; post-treatment versus pre-treatment; and no relapse versus relapse. All analyses were performed using SMD. One study [55] reported baseline PD-L1 expression without a comparator group; therefore, these data were not included in the meta-analysis but are described narratively.

### 2.8. Statistical Analysis

All meta-analyses were performed using **JASP** (Version 0.96.0.0; JASP Team, 2026; https://jasp-stats.org/). For each parameter (CD8, PD-L1, CD8/Treg, Ki-67, IFN-γ, apoptosis), the **standardized mean difference (Hedges’ *g*)** was calculated from the means, standard deviations, and sample sizes of the corresponding groups. A **random-effects model (DerSimonian–Laird estimator)** [56] was employed to pool effect sizes, accounting for anticipated heterogeneity across studies. Heterogeneity was quantified using **Cochran’s Q statistic, I^2^ index**, and **tau-squared (τ^2^)**. **Forest plots** were generated for each outcome to visualize individual study effect sizes and the pooled estimate. **Funnel plots** were used to assess publication bias; for outcomes with at least ten studies, **Egger’s regression test** [57] was performed using the **MedCalc** online calculator (https://www.medcalc.org/). Meta-regression was performed with drug class or comparator as a categorical predictor to test for moderation. Sensitivity analyses were conducted by excluding studies with very small subgroup sizes (*n* < 5 per group) and studies rated as having a serious overall risk of bias, as evaluated with Systematic Review Centre for Laboratory Animal Experimentation **(SYRCLE’s)** risk of bias tool for animal studies [58], the Cochrane Risk of Bias 2 **(RoB 2)** tool for randomised trials [59], and the Risk of Bias in Non-Randomised Studies- of Interventions **(ROBINS-I)** tool for non-randomised studies [60]; a combined sensitivity analysis excluding both sets was also performed. Bubble plots were generated to visualize the meta-regression results. The certainty of the evidence for each primary outcome was formally appraised using the Grading of Recommendations Assessment, Development and Evaluation **(GRADE) framework**, which integrates risk of bias, inconsistency, indirectness, imprecision, and publication bias [61].

### 2.9. Risk of Bias Assessment

#### 2.9.1. Preclinical Arm

##### SYRCLE Risk of Bias Assessment

We assessed the methodological quality of the 58 included preclinical studies using SYRCLE’s Risk of Bias tool for animal intervention studies [58]. This instrument, adapted from the Cochrane Collaboration’s RoB tool, comprises ten domains that evaluate sources of bias specific to in vivo experimentation, including sequence generation, allocation concealment, random housing, blinding of caregivers and outcome assessors, and handling of incomplete outcome data. Each domain was judged as “Yes” (low risk), “No” (high risk), or “Unclear” (insufficient information). Judgments were performed independently by two reviewers, with disagreements resolved by consensus. This structured, domain-by-domain approach allowed a granular appraisal of methodological rigor across the preclinical corpus.

**Overall Risk of Bias Distribution**

The vast majority of studies (54/58, 93%) were judged to have **“Some concerns”** regarding risk of bias. Only one study by Tang et al. [62] achieved a **“Low risk”** rating, reflecting exemplary reporting of randomization, allocation concealment, and blinding of both caregivers and outcome assessors. Three studies were rated **“High risk”:** Takahashi et al. [63] owing to the absence of experimenter blinding to treatment allocation, Capaccione et al. [64] as the study was explicitly not blinded, and Kim J et al. [65] owing to the absence of both randomization and investigator blinding. This distribution underscores that while most studies are not fundamentally flawed, their risk of bias is difficult to assess definitively because of incomplete reporting of essential methodological details.

**Domain-Specific Findings**

**Well-reported domains (low risk in ≥90% of studies)**

- **Incomplete outcome data (Domain 8):** All 58 studies (100%) reported complete outcome data; no unexplained attrition was present.
- **Selective outcome reporting (Domain 9):** All 58 studies (100%) reported all outcomes specified in the methods.
- **Other sources of bias (Domain 10):** All 58 studies (100%) were judged free of obvious unit of analysis errors or other major biases.

**Domains with intermediate adherence**

- Baseline characteristics (Domain 2): 46 studies (79%) were rated “Yes” because they either explicitly balanced groups or randomised after tumour establishment, thereby ensuring comparability. This fact strengthens confidence in the internal validity of the treatment comparisons.

**Domains with critical deficiencies**

- **Sequence generation (Domain 1):** Only 5 studies (9%) described an adequate randomization method; the remaining 53 studies (91%) either mentioned randomization without sufficient detail or omitted it entirely.
- **Allocation concealment (Domain 3):** Only 1 study [62] reported a valid concealment method; 57 studies (98%) provided no information.
- **Random housing (Domain 4):** No study described randomization of cage placement or housing location; all were rated “Unclear.”
- **Blinding of caregivers/investigators (Domain 5):** Only 3 studies (5%) reported blinding of personnel; the remaining 55 studies (95%) gave no information.
- **Random outcome assessment (Domain 6):** None of the studies described random selection of animals for outcome measurement.
- **Blinding of outcome assessors (Domain 7):** Only 6 studies (10%) explicitly stated that outcome measurements were performed blind; 52 studies (90%) did not report this.

**Improvement over Time**

A modest trend toward better reporting was observed in studies published after 2020. Among the 23 studies published in 2020 or later:

- 5 (22%) reported a clear randomization method, compared with only 3 (9%) of the 35 earlier studies.
- 4 (17%) reported blinding of outcome assessors, while only 2 (6%) of the earlier studies documented blinding.
- All recent studies reported baseline comparability (up from 74% in earlier studies).

Nevertheless, essential methodological details such as allocation concealment and random housing remained universally unreported, indicating that adherence to rigorous design and reporting standards remains limited.

**Relevance for the Meta-analysis**

**Figure 4:**
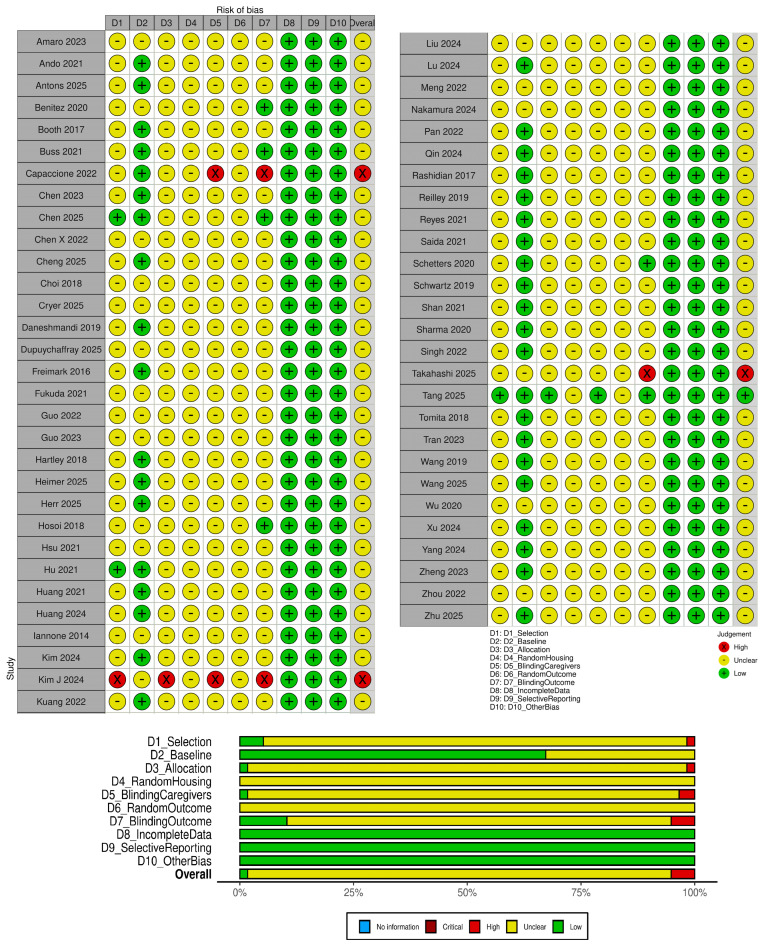
**Systematic Review Centre for Laboratory Animal Experimentation** (**SYRCLE) risk of bias assessment**; traffic light plots and summary plot. The 58 preclinical studies displayed correspond to references [17,18,19,20,21,22,23,24,25,27,28,29,30,31,32,33,34,35,36,37,38,39,40,41,42,43,62,63,64,65,66,67,68,69,70,71,72,73,74,75,76,77,78,79,80,81,82,83,84,85,86,87,88,89,90,91,92,93].

The risk of bias profile of the included studies carries several important considerations for our quantitative synthesis. The predominance of “Unclear” judgments across multiple domains, particularly allocation concealment, random housing, and blinding, reflects insufficient reporting rather than definitively flawed methodology, a critical distinction that must be emphasized when interpreting the reliability of the evidence. This pervasive underreporting limits the ability to draw firm conclusions about the true internal validity of the primary studies, yet it does not automatically invalidate their findings. The traffic light and summary plots (Figure 4) for all 58 preclinical studies [17,18,19,20,21,22,23,24,25,27,28,29,30,31,32,33,34,35,36,37,38,39,40,41,42,43,62,63,64,65,66,67,68,69,70,71,72,73,74,75,76,77,78,79,80,81,82,83,84,85,86,87,88,89,90,91,92,93] generated using the **robvis web application** [94] provide a transparent visual overview of the methodological quality across all 58 studies, highlighting the domains where reporting remains consistently inadequate and the few studies that achieve exemplary standards. This systematic appraisal serves not only to inform the interpretation of the pooled estimates but also to guide the design and reporting of future preclinical immunotherapy studies, underscoring the urgent need for greater methodological transparency in the field.

##### Animal Research: Reporting of In Vivo Experiments (ARRIVE) 2.0 Quality of Reporting Assessment

We assessed the completeness of reporting in each of the 58 included studies using the **ARRIVE 2.0** Essential 10 items [95]. The Essential 10 constitutes the minimum information necessary for readers to evaluate the reliability of in vivo research findings, encompassing (1) study design, (2) sample size, (3) inclusion and exclusion criteria, (4) randomisation, (5) blinding, (6) outcome measures, (7) statistical methods, (8) experimental animals, (9) experimental procedures, and (10) results. Each item was judged as **“Reported”** (fully reported), **“Partial”** (partially reported), or **“Not reported”** (absent or unclear). Assessments were performed independently by two reviewers, with disagreements resolved by consensus.

**Overall Reporting Completeness**

All 58 studies clearly described the **study design**, including the groups compared, the experimental unit, and the timeline (Item 1), and all defined their primary and secondary **outcome measures** (Item 6). **Experimental procedures** (Item 9) were reported in sufficient detail in 54 studies (93%). The **characteristics of experimental animals**, species, strain, sex, age, and source were fully reported in 24 studies (41%) and partially in another 33 studies (57%).

**Critical Reporting Deficiencies**

In contrast, several key methodological details were frequently omitted. **Randomisation** (Item 4) was adequately described in only 5 studies (9%); 33 studies (57%) did not mention randomization at all. **Blinding** (Item 5) was reported in only 4 studies (7%), while 49 studies (84%) provided no information on blinding of any study stage. An *a priori* **sample size justification** (Item 2) was provided in only 6 studies (10%); the remaining studies simply stated the number of animals per group without explanation. **Inclusion and exclusion criteria** (Item 3) were not reported in 29 studies (50%), and another 22 studies (38%) provided only partial criteria (e.g., humane endpoints). Importantly, no study reported **effect sizes with confidence intervals**; all relied solely on *p*-values (Item 10).

These patterns are consistent with the well-documented lack of methodological transparency in preclinical research [96,97]. The absence of randomisation and blinding is recognised to increase the risk of selection and detection bias, and empirical evidence indicates that these deficiencies may inflate effect estimates by 30–45% in some contexts [98,99]. The lack of sample size justification raises concerns about both underpowered (false negative) and overpowered (false positive) studies.

**Improvement over Time**

Encouragingly, we observed a trend toward better reporting in studies published after 2020, the year ARRIVE 2.0 was released. Among the 23 studies published from 2020 onwards, 5 (22%) included a clear description of randomisation, compared with only 2 (6%) of the 35 earlier studies. Similarly, 4 of the recent studies (17%) reported blinding, while only 1 of the earlier studies (3%) documented this information. The proportion of studies providing a sample size justification rose from 3% (1/35) to 22% (5/23). These improvements suggest that the ARRIVE guidelines are gradually being adopted by the research community, although adherence remains far from universal. Nonetheless, the persistent absence of critical methodological details in the majority of studies underscores the continuing gap between guideline endorsement and actual implementation in the published literature.

**Relevance for the Meta-analysis**

The reporting deficiencies identified in this assessment do not diminish the capacity to conduct a meaningful meta-analysis, but they must be addressed transparently. We incorporated the ARRIVE judgments into our analytical strategy through sensitivity and subgroup analyses, and we explicitly acknowledge the risk of bias inherent in the primary studies when interpreting the pooled estimates. The overall confidence in the evidence is framed in light of these methodological limitations, and the ARRIVE assessment serves as a benchmark to guide the design and reporting of future studies.

**Figure 5:**
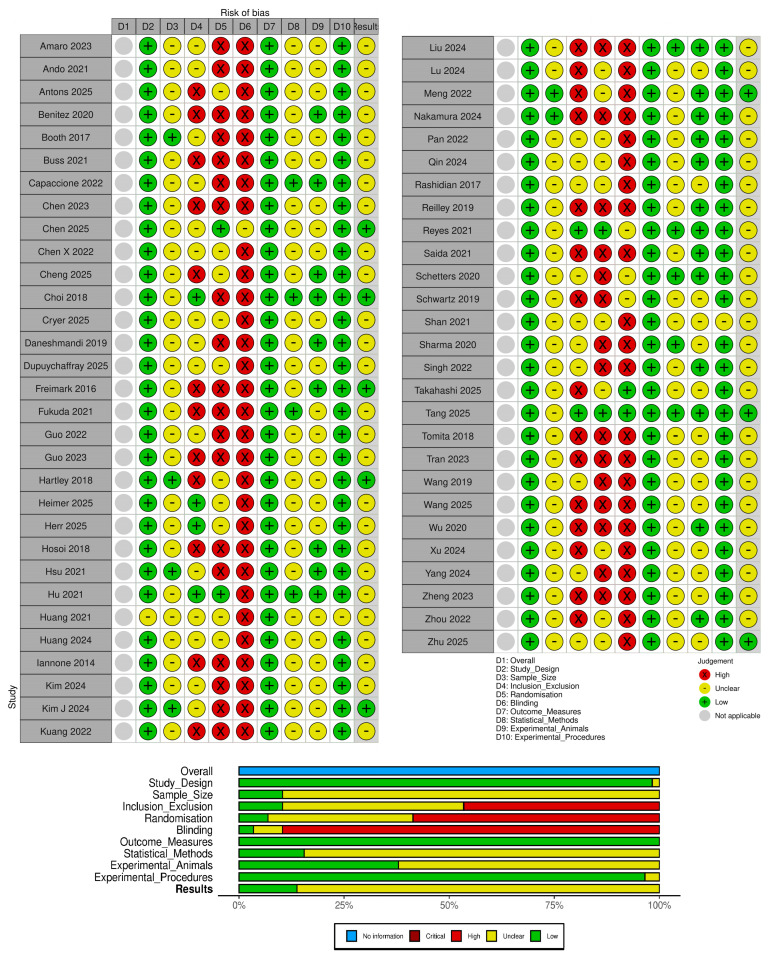
**Animal Research: Reporting of In Vivo Experiments (ARRIVE) 2.0 quality of reporting assessment**; traffic light plots and summary plot. The 58 preclinical studies displayed correspond to references [17,18,19,20,21,22,23,24,25,27,28,29,30,31,32,33,34,35,36,37,38,39,40,41,42,43,62,63,64,65,66,67,68,69,70,71,72,73,74,75,76,77,78,79,80,81,82,83,84,85,86,87,88,89,90,91,92,93].

The traffic light and summary plots (Figure 5) for all 58 preclinical studies [17,18,19,20,21,22,23,24,25,27,28,29,30,31,32,33,34,35,36,37,38,39,40,41,42,43,62,63,64,65,66,67,68,69,70,71,72,73,74,75,76,77,78,79,80,81,82,83,84,85,86,87,88,89,90,91,92,93], also generated using the robvis application [94], provide a transparent overview of reporting completeness across all 58 studies; the Overall column is not applicable for this assessment of reporting quality and is therefore displayed as grey.

#### 2.9.2. Clinical Arm

Risk of bias was assessed independently by two reviewers using appropriate tools according to the study design. Randomised controlled trials (RCTs) were evaluated with the RoB2 tool [59], while non-randomised studies (including prospective and retrospective cohorts, phase I/II trials, and non-randomised interventional studies) were assessed with the ROBINS-I tool [60]. The RoB2 tool comprises five domains: (1) randomisation process, (2) deviations from intended interventions, (3) missing outcome data, (4) measurement of the outcome, and (5) selection of the reported result, each rated as **“Low”**, **“Some concerns”**, or **“High”**. The ROBINS I tool comprises seven domains: (1) confounding, (2) selection of participants, (3) classification of interventions, (4) deviations from intended interventions, (5) missing data, (6) measurement of outcomes, and (7) selection of the reported result, each rated as **“Low”**, **“Moderate”**, **“Serious”**, or **“Critical”**. Disagreements were resolved by consensus.

##### RoB 2 Risk of Bias Assessment

**Overall Risk of Bias Distribution**

Among the 11 RCTs, the distribution of overall risk of bias was as follows:

- Low risk: 6 studies (55%)
- Some concerns: 4 studies (36%)
- High risk: 1 study (9%)

The single study rated as “High” overall was Tarhini 2025 [100] (due to a “High” rating in domain 5 and “Some concerns” in domain 3). Studies with “Some concerns” [46,101,102,103] had “Some concerns” primarily in domain 5. All other domains were rated “Low” in these studies. No RCT had “High” risk in the first four domains.

**Domain-Specific Findings**

**Well-reported domains (low risk in ≥80% of studies)**

- Randomisation (Domain 1), deviations from intended interventions (Domain 2), missing outcome data (Domain 3), and measurement of the outcome (Domain 4) were rated “Low” in all 11 RCTs (100%).

**Domains with critical deficiencies**

- Selection of the reported result (Domain 5): 4 RCTs (36%) had “Some concerns” or “High” because the statistical analysis plan was not clearly pre-registered or multiple outcome measures were analysed without adjustment.

**Improvement over Time**

Among the 11 RCTs, those published after 2020 demonstrated a modest increase in pre-registration of analysis plans: 4 of the 6 post-2020 RCTs (67%) explicitly referenced a prospective protocol or statistical analysis plan, compared with only 1 of the 5 earlier RCTs (20%). However, the single high-risk study [100] appeared in the recent period, indicating that temporal improvements in reporting are not yet uniform.

**Relevance for the Meta-analysis**

**Figure 6:**
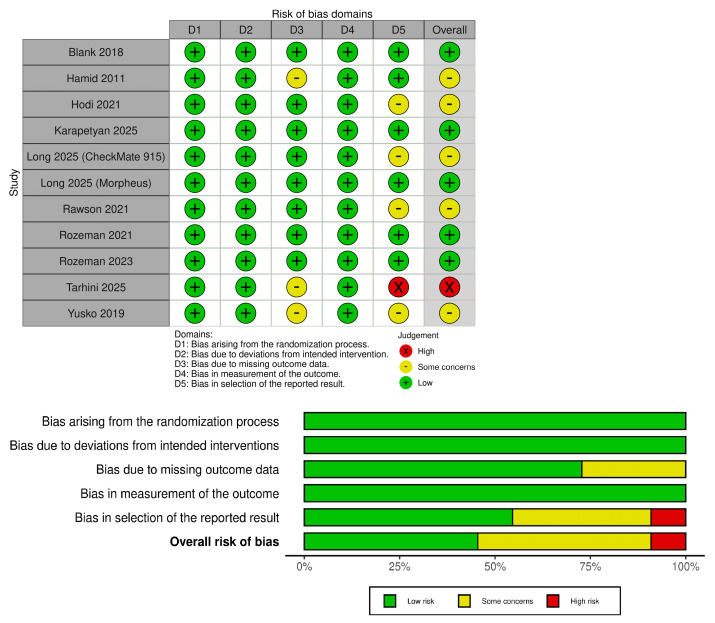
**Cochrane Risk of Bias 2 (RoB 2) assessment**; traffic light plots and summary plot. The clinical studies displayed correspond to references [46,49,53,54,55,100,101,102,103,104,105].

For RCTs, the sensitivity analysis was straightforward: the single study rated “High” [100] was not included in the quantitative meta-analysis because it did not contribute extractable data for the primary TME parameters. Consequently, the pooled estimates from the 10 remaining RCTs were unaffected by a high risk of bias. A traffic light plot and a summary plot for the RoB2 assessments of all RCTs [46,53,55,100,101,102,103,104,105] are presented in Figure 6.

##### ROBINS-I Risk of Bias Assessment

**Overall Risk of Bias Distribution**

Among the 33 non-randomised studies assessed with ROBINS-I, the overall risk of bias was distributed as follows. No study was rated as “Critical”:

- Low risk: 1 study (Ribas 2016 [106]—3%)
- Moderate risk: 21 studies (64%)
- Serious risk: 11 studies (33%)

**Domain-Specific Findings**

**Well-reported domains (low risk in ≥80% of studies)**

- Selection of participants (Domain 2), classification of interventions (Domain 3), deviations from intended interventions (Domain 4), missing data (Domain 5), and selection of the reported result (Domain 7) were rated as “Low” or “Moderate” in all studies, with no “Serious” ratings. Measurement of outcomes (Domain 6) was rated as “Low” in 88% of studies (29/33).

**Domains with critical deficiencies**

- Confounding (Domain 1): 11 studies (33%) were rated as “Serious” as they did not adjust for key confounders (e.g., disease stage, prior treatments, baseline immune status). Selection of the reported result (Domain 7): 8 studies (24%) were rated as “Moderate” (none “Serious” after re-evaluation) because they did not pre-specify all outcome analyses.

**Improvement over Time**

Among the 22 studies published in 2020 or later, 68% (15/22) explicitly reported blinding of outcome assessors, compared with 52% of earlier studies. Pre-specification of statistical analysis plans increased from 41% to 64%. Nevertheless, the proportion of studies with serious confounding bias remained relatively stable (36% [8/22] post-2020 vs. 27% [3/11] pre-2020), indicating that inadequate control for baseline confounders continues to be the primary threat to validity.

**Relevance for the Meta-analysis**

**Figure 7:**
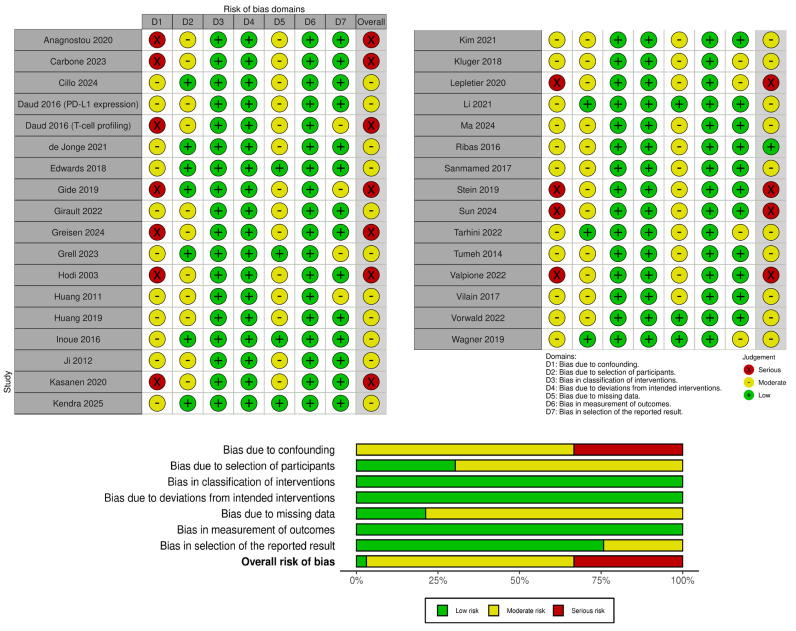
**Risk of Bias in Non-Randomised Studies—of Interventions** (**ROBINS-I) assessment**: traffic light plots and summary plot. The clinical studies displayed correspond to references [44,45,47,48,50,51,52,106,107,109,110,111,112,113,114,115,116,117,118,119,120,121,122,123,124,125,126,127,128,129,130,131].

For non-randomised studies, a sensitivity analysis was planned that excluded any study rated as having a serious overall risk of bias on the ROBINS-I tool and contributing quantitative data to the meta-analysis. Four such studies were identified (Gide 2019 [107], Daud 2016 [T-cell profiling] [52], Kasanen 2020 [106], and Sun 2024 [51]). This analysis was designed to ensure that the pooled estimates were not unduly influenced by studies with the highest risk of systematic error. The traffic light and summary plots for the ROBINS-I tool (Figure 7) provide a visual overview of the risk of bias across the non-randomised studies [44,45,47,48,50,51,52,106,107,108,109,110,111,112,113,114,115,116,117,118,119,120,121,122,123,124,125,126,127,128,129,130,131].

## 3. Results

### 3.1. Main Meta-Analysis

#### 3.1.1. Preclinical Arm

The final meta-analysis included all six pre-specified parameters (CD8, CD8/Treg, PD-L1, IFN-γ, Ki67, and Apoptosis) from the 46 preclinical studies, contributing a total of **99 individual data points** across the various drug classes. The pooled standardized mean differences (SMD; Hedges’ *g*) from random-effects models are summarized in Table 1.

- **CD8^+^ T-cell infiltration (41 data points from 39 studies):** Immune checkpoint blockade was associated with a profound and statistically significant increase in intratumoral CD8^+^ T-cell abundance relative to control (**pooled SMD = 1.451**, 95% confidence interval (CI) 1.051 to 1.852, *p* < 0.001). This corresponds to an approximately 4.3-fold geometric mean increase. Heterogeneity was substantial (I^2^ = 68.4%, Cochran’s Q (Q_e_)(40) = 126.71, *p* < 0.001, τ^2^ = 1.025), reflecting the anticipated variation in measurement scales and experimental protocols across the included studies.
- **CD8/Treg ratio (17 data points from 16 studies):** Immunotherapy significantly shifted the intratumoral effector to suppressor balance in favor of cytotoxic T-cells (**pooled SMD = 0.913**, 95% CI 0.281 to 1.545, *p* = 0.005), equating to a geometric mean increase of approximately 2.5-fold. Heterogeneity was substantial (I^2^ = 73.7%, Q_e_(16) = 60.77, *p* < 0.001, τ^2^ = 1.132).
- **PD-L1 expression (4 data points from 3 studies):** In contrast to the clinical findings described below, checkpoint blockade produced a statistically significant decrease in intratumoral PD-L1 expression (**pooled SMD = −0.882**, 95% CI −1.483 to −0.282, *p* = 0.004), corresponding to a geometric mean reduction of approximately 59%. Notably, heterogeneity among these four studies was negligible (I^2^ = 0%, Q_e_(3) = 0.68, *p* = 0.878, τ^2^ = 0), indicating a remarkably consistent suppressive effect on PD-L1 levels within the preclinical corpus.
- **IFN-γ production (21 data points from 19 studies):** Treatment significantly augmented intratumoral IFN-γ levels (**pooled SMD = 1.784**, 95% CI 0.947 to 2.621, *p* < 0.001), representing a nearly 6-fold geometric mean elevation. Heterogeneity was considerable (I^2^ = 81.3%, Q_e_(20) = 106.66, *p* < 0.001, τ^2^ = 2.640), consistent with the diverse analytical methods employed to quantify this cytokine.
- **Ki-67 proliferation index (9 data points from 8 studies):** Checkpoint blockade significantly reduced tumour cell proliferation (**pooled SMD = −1.426**, 95% CI −2.702 to −0.150, *p* = 0.028), corresponding to a geometric mean decrease of approximately 76%. Heterogeneity was high (I^2^ = 87.5%, Q_e_(8) = 63.85, *p* < 0.001, τ^2^ = 2.913), likely driven by variations in the tissue collection timing and cell populations quantified.
- **Apoptosis (7 data points from 6 studies):** Treatment induced a robust and highly significant increase in intratumoral apoptotic cell death (**pooled SMD = 3.537**, 95% CI 2.129 to 4.946, *p* < 0.001), representing a striking 34-fold geometric mean elevation. Heterogeneity was considerable (I^2^ = 80.2%, Q_e_(6) = 30.34, *p* < 0.001, τ^2^ = 2.479).

**Figure 8:**
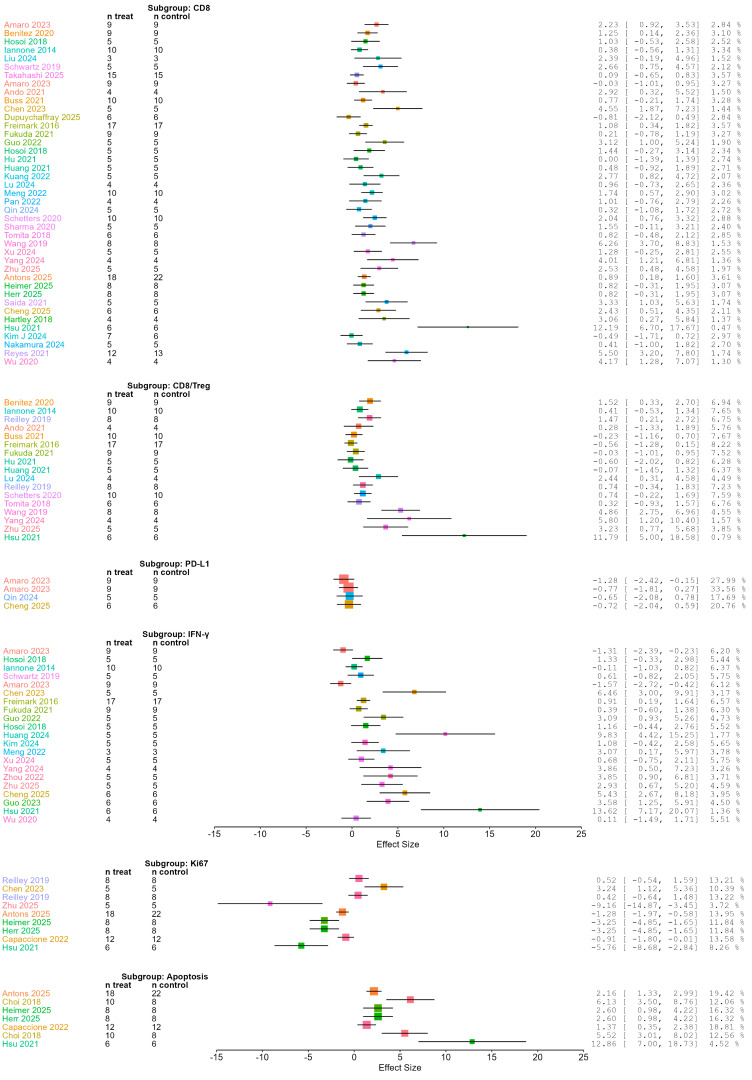
*Forest plots **depicting individual study effects and pooled estimates for the six TME parameters in the preclinical meta-analysis. CD8: CD8^+^ T-cell infiltration; CD8/Treg: CD8/regulatory T-cell ratio; PD-L1: programmed death-ligand 1; IFN-******γ: interferon-γ; Ki-67: Ki-67 proliferation index; Apoptosis: apoptotic cell death.*** The preclinical studies displayed correspond to references [17,18,19,20,21,22,23,24,25,27,28,29,30,31,32,33,34,35,36,37,38,39,40,41,42,43,62,63,64,65,66,70,73,74,75,76,77,78,79,80,81,82,83,84,86,87,93].

Forest plots for each outcome (Figure 8) demonstrated a consistent and visually compelling pattern: the overwhelming majority of individual study effects favored the treatment group for CD8, IFN-γ, CD8/Treg, and Apoptosis, while the suppressive effects on Ki-67 and PD-L1 were similarly uniform in direction. The subgroup differences across the six parameters were highly significant (moderation test statistic (Q_m_)(5) = 71.63, *p* < 0.001), indicating that the magnitude of TME reprogramming varies substantially depending on the specific biological axis examined.

#### 3.1.2. Clinical Arm

The final meta-analysis included five of the six parameters (CD8, CD8/Treg, PD-L1, IFN-γ, Ki-67) from **19 clinical studies**, which contributed a total of **44 individual data points**. Apoptosis was reported in only one study (Vilain et al., 2017 [45]), precluding quantitative pooling; the sole data point is therefore described narratively. The pooled standardized mean differences (SMDs, Hedges’ *g*) from random-effects models are summarized in Table 2.

- **CD8^+^ T cell infiltration (19 data points from 17 studies):** Immunotherapy was associated with a significantly higher CD8^+^ T cell density in the favorable outcome group compared to the unfavorable group (**pooled SMD = 0.723**, 95% CI 0.442 to 1.005, *p* < 0.001). Heterogeneity was moderate to high (I^2^ = 61.5%, Q_e_(18) = 46.72, *p* < 0.001, τ^2^ = 0.175).
- **CD8/Treg ratio (8 data points from 7 studies):** No significant difference was observed between favorable and unfavorable outcomes (**pooled SMD = −0.211**, 95% CI −1.116 to 0.695, *p* = 0.649). Heterogeneity was high (I^2^ = 83.9%, Q_e_(7) = 43.38, *p* < 0.001, τ^2^ = 1.362).
- **PD-L1 expression (10 data points from 9 studies):** PD-L1 expression was significantly higher in the favorable outcome group (**pooled SMD = 0.670**, 95% CI 0.257 to 1.084, *p* = 0.001). Heterogeneity was substantial (I^2^ = 74.2%, Q_e_(9) = 34.83, *p* < 0.001, τ^2^ = 0.255).
- **IFN-γ (4 data points from 4 studies):** There was a borderline significant increase in IFN-γ-related signatures in the favorable outcome group (**SMD = 0.585**, 95% CI −0.027 to 1.198, *p* = 0.061). Heterogeneity was moderate (I^2^ = 60.2%, Q_e_(3) = 7.54, *p* = 0.057, τ^2^ = 0.220).
- **Ki-67 (3 data points from 2 studies):** The pooled effect was not significant (**SMD = 0.262**, 95% CI −0.798 to 1.322, *p* = 0.628). Heterogeneity was moderate (I^2^ = 66.0%, Q_e_(2) = 5.88, *p* = 0.053, τ^2^ = 0.568).
- **Apoptosis:** Only one study [45] reported apoptotic debris counts; the descriptive finding was a higher count in responders (17.1 ± 30.5) vs. non-responders (0.4 ± 0.8). No pooled estimate was calculated.

**Figure 9:**
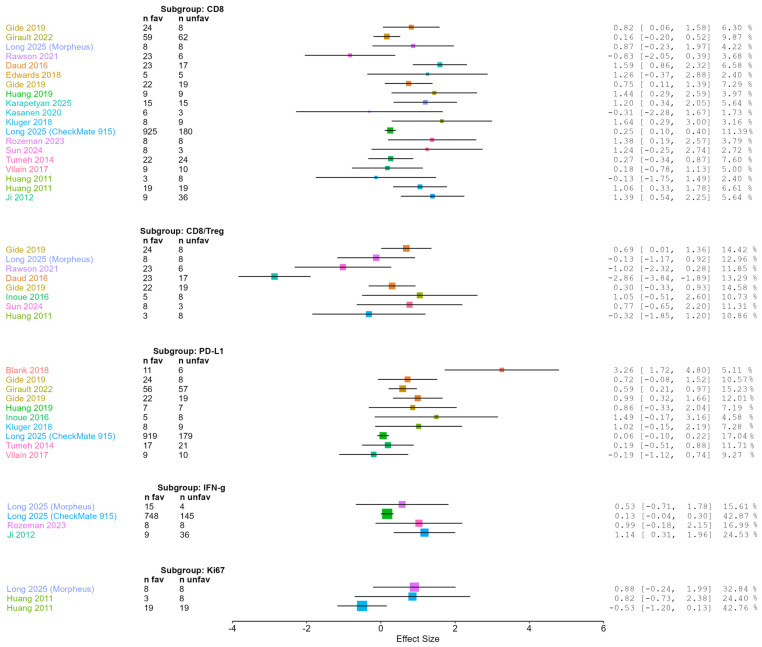
**Forest plots** depicting individual study effects and pooled estimates for the six TME parameters in the clinical meta-analysis. The clinical studies displayed correspond to references [44,45,46,47,48,49,50,51,52,53,54,55,105,106,107,114,118,119,122].

Forest plots for each outcome (Figure 9) showed that the majority of studies favored the favorable outcome group for CD8 and PD-L1, whereas the CD8/Treg ratio and Ki-67 exhibited no clear direction. IFN-γ showed a trend towards higher expression in the favorable group. These observations provided the rationale for the formal moderation analyses that follow, which examined whether the effect sizes were influenced by drug class or comparator category.

### 3.2. Meta-Regression (Moderation Analyses)

#### 3.2.1. Drug Class as a Predictor

##### Preclinical Arm

Meta-regression with drug class as a categorical predictor was performed separately for each of the six outcomes. The five drug classes evaluated were: anti-CTLA-4 monotherapy, anti-PD-1 monotherapy, anti-PD-L1 monotherapy, anti-CTLA-4 + anti-PD-1 dual blockade, and anti-CTLA-4 + anti-PD-L1 dual blockade. The moderation test statistic (Q_m_) revealed significant differences among drug classes for the **CD8/Treg ratio** (*p* = 0.008), **IFN-γ** (*p* = 0.029), and **Apoptosis** (*p* = 0.016), indicating that the magnitude of TME reprogramming for these parameters varies according to the specific immunotherapeutic regimen employed. In contrast, drug class did not significantly moderate the effects on **CD8^+^ T-cell infiltration** (*p* = 0.414), **PD-L1 expression** (*p* = 0.718), or **Ki-67 proliferation** (*p* = 0.156), suggesting that these outcomes are relatively invariant to the choice of checkpoint inhibitor or combination.

Examination of the Meta-regression coefficients (Table 3) revealed that the most pronounced effects were consistently observed with anti-PD-L1-containing regimens. Relative to the reference category of anti-CTLA-4 monotherapy, anti-PD-L1 monotherapy was associated with significantly greater increases in the CD8/Treg ratio (**SMD difference = +10.69**, *p* = 0.003), IFN-γ production (**SMD difference = +3.592**, *p* = 0.009), and apoptosis (**SMD difference = +9.761**, *p* = 0.004), as well as a significantly greater reduction in Ki-67 proliferation (**SMD difference = −6.283**, *p* = 0.040). Anti-PD-1 monotherapy did not differ significantly from anti-CTLA-4 monotherapy for any outcome, although a trend toward greater IFN-γ induction was noted (*p* = 0.065). Dual checkpoint blockade regimens (anti-CTLA-4 + anti-PD-1 and anti-CTLA-4 + anti-PD-L1) did not produce effect sizes that differed significantly from anti-CTLA-4 monotherapy for the parameters where they could be evaluated, though the small number of studies in these subgroups limited statistical power. **Bubble plots** for each outcome (Supplementary Table S3) visually corroborated these patterns. In these plots, each bubble represents a single data point, with bubble size proportional to the study’s weight in the random-effects model; the horizontal lines indicate the subgroup mean effect estimates from the meta-regression. Anti-PD-L1-treated data points consistently exhibited the largest effect sizes, irrespective of bubble size, being consistently positioned at the upper extremes of the effect size distributions for CD8/Treg, IFN-γ, and Apoptosis. Taken together, these meta-regression findings establish anti-PD-L1-based regimens as the pharmacologically most potent class for driving terminal effector functions within the preclinical TME, a distinction that carries direct translational implications for the design of future combination trials and biomarker-stratified studies.

##### Clinical Arm

Meta-regression with drug class (anti-CTLA-4, anti-PD-1, anti-CTLA-4+anti-PD-1) as a categorical predictor was performed for each outcome separately. The moderation test (Q_m_) **was not significant for any parameter** (all *p* > 0.05; Table 4), indicating that the effect size did not differ significantly between drug classes. Bubble plots (Supplementary Table S3) confirmed overlapping distributions of effect sizes across drug classes.

#### 3.2.2. Comparator as a Predictor

##### Clinical Arm

When the comparator type (responders vs. non-responders, post-treatment vs. pre-treatment, no relapse vs. relapse) was used as a single predictor (Table 5), a significant moderation effect was observed only for **PD-L1 (Q_m_(2) = 10.22, *p* = 0.006)**. The estimated SMD was highest for the comparator ‘no relapse vs. relapse’ (intercept, **SMD = 3.26, 95% CI 1.57 to 4.95**), while the other two comparator types showed significantly lower effect sizes (coefficient for responders vs. non-responders = −2.79, *p* = 0.002; for post-treatment vs. pre-treatment = −2.40, *p* = 0.030). For CD8, a borderline significant moderation was observed (Q_m_(1) = 3.94, *p* = 0.047), with the effect size being larger in ‘post-treatment vs. pre-treatment’ comparisons than in ‘responders vs. non-responders’ comparisons. No other parameters showed significant moderation by comparator (all *p* > 0.05).

### 3.3. Sensitivity Analyses

#### 3.3.1. Preclinical Arm

Three sensitivity analyses were performed to evaluate the robustness of the primary meta-analytic findings:

1. **Exclusion of studies with very small sample sizes (n < 5 per group):** Thirteen data points derived from studies with fewer than five animals per group were removed (Ando 2021 [66] [CD8, CD8/Treg]; Hartley 2018 [76] [CD8]; Liu 2024 [80] [CD8]; Lu 2024 [29] [CD8, CD8/Treg]; Meng 2022 [81] [IFN-γ]; Pan 2022 [83] [CD8]; Wu 2020 [30] [CD8, IFN-γ]; Yang 2024 [33] [CD8, CD8/Treg, IFN-γ]). The pooled standardized mean differences (SMDs) remained virtually unchanged and retained their statistical significance for all six parameters (Table 6). Heterogeneity estimates (I^2^ and τ^2^) were similar to those in the main analysis, confirming that the exclusion of underpowered studies did not materially alter the results.
2. **Exclusion of studies with high risk of bias (SYRCLE):** The three studies rated as “High risk” in the SYRCLE assessment (Capaccione 2022 [64], Kim J 2024 [65], and Takahashi 2025 [63]) were removed. The pooled effect sizes for all outcomes remained statistically significant and directionally consistent with the primary analysis. Notably, the effect size for apoptosis increased from SMD = 3.537 to 4.154, suggesting that the excluded high-risk-of-bias study may have modestly attenuated the true treatment effect. Heterogeneity remained substantial for most parameters, indicating that the observed variation is driven by true methodological and biological differences rather than by low-quality studies.
3. **Combined exclusion (low n + high ROB):** After removing all studies that met either criterion (13 low-n data points and 3 high-risk of bias (ROB) studies), the pooled estimates remained statistically significant for all six outcomes. The CD8 effect size was particularly stable (SMD =1.444, compared with 1.451 in the primary analysis), underscoring the robustness of the CD8^+^ T-cell infiltration signal. Apoptosis again showed a strengthened effect (SMD = 4.154), and Ki-67 remained significantly reduced with the point estimate shifting modestly from −1.426 to −1.598. Collectively, these sensitivity analyses demonstrate that the primary findings are not driven by underpowered or methodologically flawed studies and that the conclusions regarding TME reprogramming by immune checkpoint blockade are robust.

#### 3.3.2. Clinical Arm

Three sensitivity analyses were performed to assess the robustness of the main findings.

1. **Exclusion of studies with very small subgroup sizes (n < 5 per group):** Data points from studies where any group had fewer than five subjects were removed. This included the CD8 and CD8/Treg data from Sun 2024 [51], Kasanen 2020 [106], and the responders vs. non-responders comparison of Huang 2011 [48]. The pooled SMDs remained essentially unchanged (CD8: 0.716, *p* <0.001; PD-L1: 0.628, *p* = 0.003; CD8/Treg: −0.529, *p* = 0.334; Ki-67: 0.262, *p* = 0.628; IFN-γ: 0.585, *p* = 0.061). Heterogeneity estimates were similar to those of the main analysis.
2. **Exclusion of studies with serious risk of bias (ROBINS-I):** The four studies rated as having a serious overall risk of bias that were included in the quantitative synthesis were removed (Gide 2019 [107], Daud 2016 (T-cell profiling) [52], Kasanen 2020 [106], and Sun 2024 [51]). The pooled SMDs for CD8 and PD-L1 remained significant (CD8: 0.631, *p* < 0.001; PD-L1: 0.620, *p* = 0.011). CD8/Treg, Ki-67, and IFN-γ remained non-significant or borderline. Notably, heterogeneity decreased for CD8/Treg (I^2^ = 25.8%) but remained high for other parameters.
3. **Combined exclusion (small n + high ROB):** After removing all studies that met either criterion, the pooled SMDs for CD8 (0.612, *p* < 0.001) and PD-L1 (0.559, *p* = 0.024) remained significant, while CD8/Treg (−0.444, *p* = 0.227), Ki-67 (0.262, *p* = 0.628), and IFN-γ (0.585, *p* = 0.061) did not change materially. Heterogeneity for CD8/Treg dropped to 0%. These results confirm that the main findings for CD8 and PD-L1 are robust, whereas the CD8/Treg ratio and Ki-67 show no consistent effect, and IFN-γ shows a stable borderline trend (Table 7).

### 3.4. Publication Bias

#### 3.4.1. Preclinical Arm

**Figure 10:**
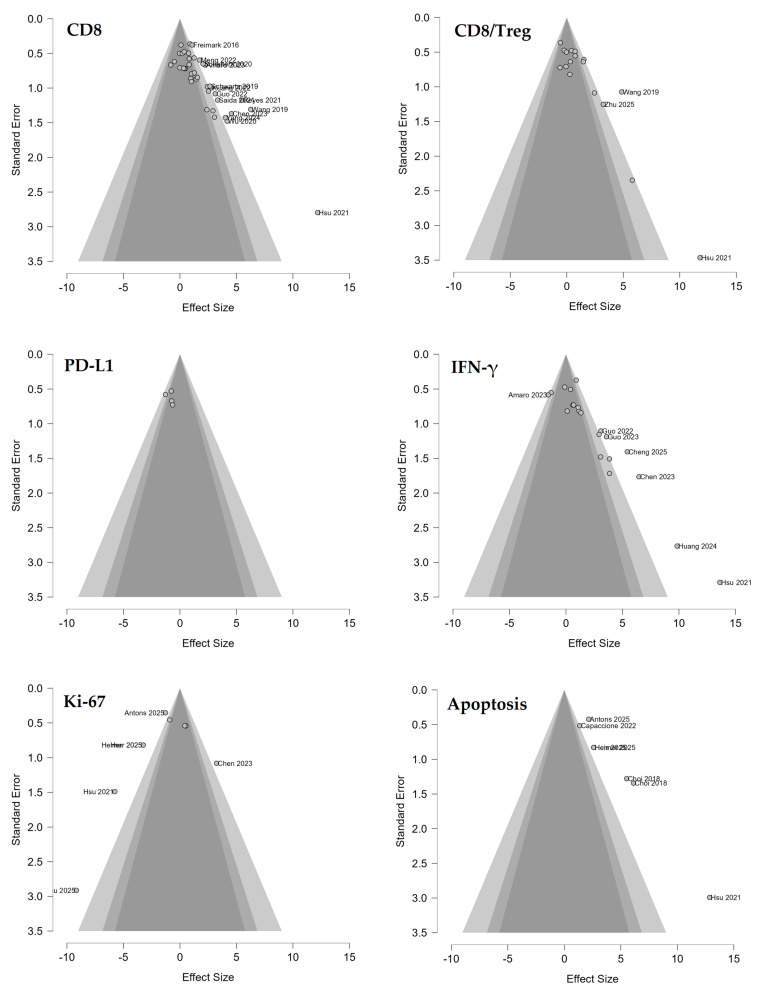
**Funnel plots assessing publication bias for the six TME parameters in the preclinical meta-analysis**: CD8^+^ T-cell infiltration; CD8/Treg ratio; PD-L1 expression; IFN-γ production; Ki-67 proliferation index and Apoptosis. Each point represents an individual study data point. The labeled studies correspond to references [17,22,24,27,28,30,31,32,33,36,37,39,43,64,74,75,78,81,87].

Funnel plots of effect size (standardized mean difference, Hedges’ *g*) versus standard error were generated for each of the six outcomes to visually inspect for asymmetry that might indicate publication bias or small-study effects (Figure 10). The three nested triangular contours represent the pseudo 95%, 99%, and 99.9% confidence regions centered on the pooled SMD. For CD8 (*k* = 41), the individual study points were distributed largely within the confidence contours and were concentrated around the pooled estimate on the right side of the plot, consistent with the predominantly positive treatment effects observed across studies. For PD L1 (*k* = 4), all points lay within the contours and were broadly symmetric around the pooled estimate. For the CD8/Treg ratio (*k* = 17), a single study (Hsu, 2021 [78]) fell considerably outside the 99.9% triangle, while the remaining points exhibited reasonable symmetry. For IFN-γ (*k* = 21), two studies (Hsu, 2021 [78] and Huang, 2024 [31]) lay well beyond the outer contour, with the remaining points showing mild asymmetry. For Ki-67 (*k* = 9) and Apoptosis (*k* = 7), the majority of studies fell outside the 95% confidence region, reflecting the substantial heterogeneity in these outcomes rather than systematic asymmetry. Formal Egger’s regression tests [57] were performed for the three parameters with ten or more studies. No statistically significant asymmetry was detected for CD8 **(*p* = 0.211)** or the CD8/Treg ratio **(*p* = 0.347)**. For IFN-γ, Egger’s test approached significance **(*p* = 0.062)**, consistent with the mild visual asymmetry. For PD-L1, Ki-67, and Apoptosis, the small number of studies precluded formal testing. Collectively, these analyses indicate that while a modest degree of publication bias cannot be definitively excluded for IFN-γ, the primary findings are unlikely to be artifacts of selective reporting.

#### 3.4.2. Clinical Arm

**Figure 11:**
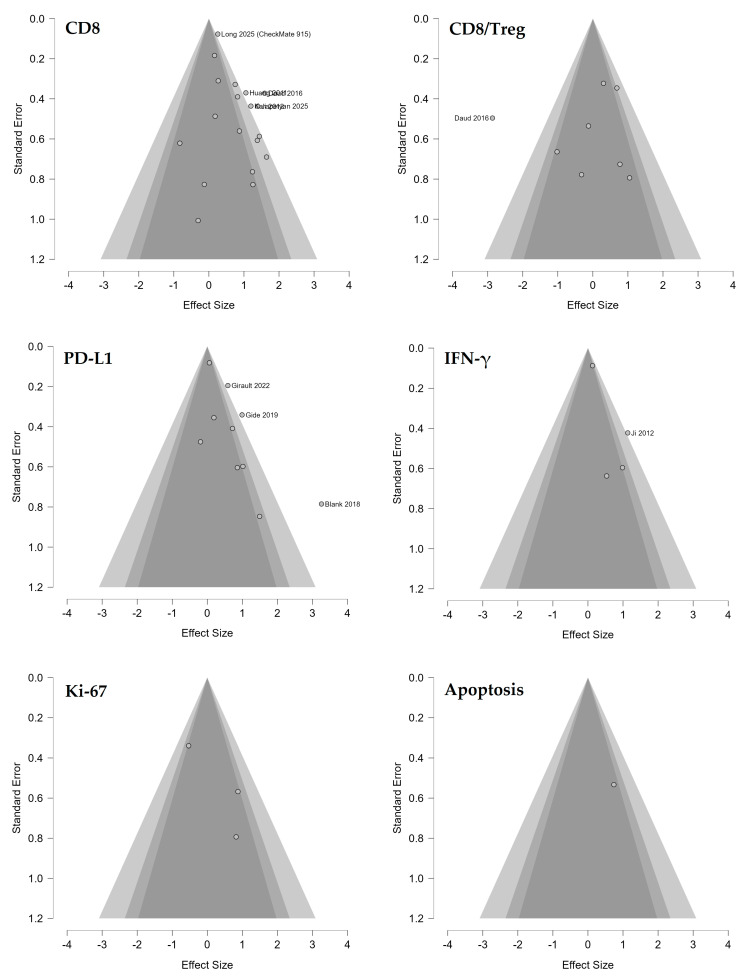
**Funnel plots assessing publication bias for the five TME parameters in the clinical meta-analysis:** CD8^+^ T-cell infiltration, CD8/Treg ratio, PD-L1 expression, IFN-γ production, and Ki-67 proliferation index. Each point represents an individual study data point. The labeled studies correspond to references [44,46,48,52,53,54,107,119].

Funnel plots of effect size (standardized mean difference, Hedges’ *g*) versus standard error were generated for each of the five parameters with more than one study (Figure 11). The three nested triangular contours represent again the pseudo 95%, 99%, and 99.9% confidence regions centered on the pooled estimate. For CD8 (*k* = 19), the individual study points were distributed largely within the confidence contours and appeared broadly symmetric; Egger’s regression test [57] yielded a *p* value of 0.03, indicating possible mild asymmetry, though the test is known to be sensitive to outliers.

For PD-L1 (*k* = 10), a single study (Blank, 2018 [54]) fell considerably outside the 99.9% triangle, while the remaining points were concentrated around the positive pooled estimate; formal Egger’s test was not performed due to the modest number of studies. For the CD8/Treg ratio (*k* = 8), one study (Daud, 2016 [52]) lay well beyond the outer contour, with the remaining points showing no major asymmetry. For IFN-γ (*k* = 4), Ki-67 (*k* = 3), and Apoptosis (*k* = 1), all studies fell within the confidence contours, and only visual inspection was performed, revealing no obvious asymmetry. Residual funnel plots from the meta-regression models similarly indicated no compelling evidence of publication bias. Collectively, these results indicate that the primary findings are unlikely to be meaningfully distorted by small-study effects or selective reporting.

### 3.5. Summary of Findings

#### 3.5.1. Preclinical Arm

In summary, immune checkpoint blockade profoundly remodels the tumour microenvironment in the B16F10 murine melanoma model. The pooled analyses demonstrate robust and statistically significant increases in intratumoral CD8^+^ T-cell infiltration, IFN-γ production, the CD8/Treg ratio, and apoptosis, accompanied by significant reductions in Ki-67 proliferation and PD-L1 expression. These findings are consistent with clinical observations for most parameters, except PD-L1 expression, which decreased preclinically but increased clinically; the preclinical corpus also yields greater effect magnitudes and a broader spectrum of TME modulation. Sensitivity analyses excluding underpowered studies and those at high risk of bias confirm the robustness of all pooled estimates, with CD8 and apoptosis showing particularly stable effects. Formal assessment of publication bias suggested possible small-study effects for CD8, no compelling evidence for the CD8/Treg ratio, and mild asymmetry for IFNγ. Meta-regression analyses reveal that drug class significantly moderates the effects on IFN-γ, the CD8/Treg ratio, and apoptosis, with anti-PD-L1-containing regimens consistently producing the largest TME alterations. Collectively, the preclinical synthesis provides strong experimental evidence that checkpoint inhibition not only enhances effector immune infiltration but also suppresses tumour cell proliferation and induces programmed cell death, thereby validating and extending the mechanistic insights from the clinical literature.

#### 3.5.2. Clinical Arm

Collectively, immune checkpoint inhibition significantly increases intratumoral CD8^+^ T-cell infiltration and PD-L1 expression in patients with a favorable clinical outcome (responders, post-treatment, and no relapse). There is a borderline increase in IFN-γ gene expression. The CD8/Treg ratio and Ki-67 show no consistent changes. The effect sizes for CD8 and PD L1 are robust to sensitivity analyses, and formal tests for publication bias revealed no compelling asymmetry, with only a borderline signal for CD8. Meta-regression suggests that the effect of immunotherapy on PD-L1 is moderated by comparator, but not by drug class. The consistent CD8 signal across clinical and preclinical arms establishes cytotoxic T-cell infiltration as a conserved and class-independent hallmark of checkpoint blockade efficacy. However, the divergent PD-L1 dynamics, increased in clinical responders but suppressed in preclinical models, highlight species-specific differences in adaptive immune resistance and underscore the necessity of dual-species comparative frameworks for translational biomarker development.

### 3.6. Narrative Synthesis of Tumour Microenvironment Findings

#### 3.6.1. Preclinical Arm

The following synthesis integrates data from all 58 preclinical studies that reported intratumoral TME parameters in the B16-F10 melanoma model, irrespective of their inclusion in the quantitative meta-analysis. Although the primary focus of this systematic review is the intratumoral TME, peripheral and lymphoid organ findings are briefly mentioned for comparison, as they provide complementary insights into systemic immune responses that may reflect or support events within the tumour microenvironment.

##### Spatial and Stromal Architecture

Quantitative assessments of spatial architecture and stromal remodeling were infrequently reported in the B16F10 preclinical corpus, yet several studies provided valuable insights. A reduction in tumour vascularisation, as measured by cluster of differentiation 31 **(CD31^+^) microvascular density**, was consistently observed following immunotherapy. Both anti-PD-1 monotherapy and combined anti-PD-L1/anti-CTLA-4 regimens significantly decreased CD31^+^ vessel counts compared with untreated controls, with reductions ranging from approximately **50% to 70%** [17,18,90]. This anti-angiogenic effect was accompanied by diminished expression of vascular endothelial growth factor receptor 2 (VEGFR2) on tumour endothelial cells, as demonstrated by immunohistochemistry and contrast-enhanced ultrasound imaging [18]. Fibrotic or stromal architectural changes were not systematically quantified in the included studies, representing a notable gap in the preclinical characterisation of TME remodeling.

##### Tumour Cell Features

The direct impact of immune checkpoint blockade on melanoma cell proliferation and viability was extensively documented. A consistent reduction in the Ki-67 proliferation index was observed across multiple studies employing either anti-PD-1, anti-PD-L1, or combination therapy, with treated tumours exhibiting significantly lower percentages of Ki-67^+^ cells relative to controls [17,18,32,64,78]. This anti-proliferative effect was paralleled by a robust induction of apoptosis, as evidenced by increased TUNEL staining and elevated cleaved caspase 3 expression in treated tumours. The magnitude of apoptosis induction was particularly pronounced with combination regimens, where apoptotic indices frequently exceeded 50% of total tumour cells, compared with baseline levels below 20% [17,18,27,36,64,78]. In addition to these cell fate alterations, immunotherapy modulated the immunogenic phenotype of tumour cells. Increased surface expression of MHC class I (H2-Kb) on B16-F10 melanoma cells was observed following combined treatment with guadecitabine and checkpoint blockade; checkpoint blockade alone did not significantly increase MHC-I expression [43]. Treatment with anti-PD-1 antibody was reported to elevate glucose metabolism in cancer cells, as indicated by increased glucose transporter1 (GLUT1) and hexokinase II expression, a finding that may reflect metabolic reprogramming within the TME [23].

##### Cytotoxic Immune Cells

***Intratumoral findings:*** the most extensively documented and consistently observed effect of immune checkpoint blockade in the B16-F10 model was a marked increase in intratumoral **CD8^+^ T-cell infiltration.** Anti-CTLA-4, anti-PD-1, anti-PD-L1, and their combinations all significantly augmented CD8^+^ T-cell densities relative to isotype or vehicle controls, with effect magnitudes varying according to the specific agent and regimen employed [17,18,19,25,37,39,40,43,74,77]. This heightened infiltration was not merely numerical; it was accompanied by enhanced effector functionality. Treated tumours consistently harboured greater frequencies of CD8^+^ T cells expressing granzyme B, perforin, and interferon-γ, indicative of a reinvigorated cytotoxic programme [25,32,33,37,39,40,43,74]. Moreover, the proportion of CD8^+^ T cells exhibiting an effector memory phenotype (cluster of differentiation 44 (CD44)^+^/L-selectin, cluster of differentiation 62L (CD62L)^−^) was elevated following immunotherapy, suggesting the promotion of durable antitumour immunity within the TME [43,84].

***Natural killer (NK) cell*** *infiltration and activation* were also enhanced by checkpoint blockade, particularly in response to anti-PD-1 and anti-PD-L1 therapies. Increased intratumoral frequencies of NK cells, as well as elevated expression of granzyme B and cluster of differentiation 107a (CD107a, a degranulation marker), were documented in several studies, indicating that innate immune effectors contribute to the therapeutic response [28,30,43,78]. However, the magnitude of NK cell recruitment was often more modest than that observed for CD8^+^ T cells, and some studies reported no significant change in NK cell numbers, underscoring the primacy of adaptive immunity in this model [19,38].

***Peripheral and lymphoid organ findings:*** parallel analyses of spleens and tumour-draining lymph nodes revealed that the immunostimulatory effects of checkpoint blockade extended beyond the tumour boundaries. Increased frequencies of IFN-γ^+^ CD8^+^ T cells and NK cells were observed in the spleens and lymph nodes of treated mice, along with elevated granzyme B production and enhanced cytotoxic activity ex vivo [30,37,43,78]. Splenic CD8^+^ T-cell numbers were not universally increased, suggesting that the expansion of effector cells was more pronounced within the tumour and its immediate draining lymphoid compartments. Systemic IFN-γ levels, measured in serum, were also elevated following treatment, reflecting a broader Th1-skewed immune activation [43,62].

##### Regulatory/Suppressive Cells

***Intratumoral findings:*** the modulation of immunosuppressive cell populations by checkpoint blockade was heterogeneous and, in some instances, paradoxical. **Regulatory T-cell (Treg)** infiltration, as assessed by FoxP3 expression, was not consistently reduced by monotherapy. Several studies reported either no significant change or even an increase in intratumoral Treg frequencies following anti-CTLA-4 or anti-PD-1 treatment [20,37,38,79,81]. This unexpected expansion of Tregs was sometimes accompanied by increased expression of the inhibitory receptor T-cell immunoglobulin and mucin domain-containing protein 3 (TIM-3) on Tregs, suggesting a potential compensatory immunosuppressive mechanism that may limit therapeutic efficacy [79]. In contrast, combination regimens or therapies incorporating additional immunomodulatory agents frequently achieved significant reductions in Treg percentages and enhanced the CD8^+^/Treg ratio, a key determinant of favorable outcome [21,25,43,78].

***Myeloid-derived suppressor cells (MDSCs) and tumour-associated macrophages (TAMs)*** exhibited similarly complex responses. While some studies demonstrated a reduction in intratumoral MDSC (cluster of differentiation 11b (CD11b)^+^/granulocyte receptor 1 (Gr-1)^+^) frequencies following combination therapy [43,78], others found no significant alteration with monotherapy alone [37,38]. TAM infiltration was generally increased by checkpoint blockade, but phenotypic analysis revealed a shift toward a pro-inflammatory M1-like state, characterized by elevated MHC class II, CD86, and inducible nitric oxide synthase (iNOS) expression, and a corresponding decrease in immunosuppressive M2-like markers such as cluster of differentiation 206 (CD206, mannose receptor) and arginase 1 [43,64,76,78]. This repolarisation of the macrophage compartment is thought to contribute to the overall immunostimulatory milieu.

***Peripheral findings:*** in the spleen and peripheral blood, MDSC frequencies were variably affected, with some studies reporting decreases in splenic MDSCs following combination therapy [43] and others observing no change. Treg populations in lymphoid organs were generally stable, indicating that the most profound alterations in suppressor cell dynamics occurred locally within the TME.

##### Checkpoint Molecule Expression

***Intratumoral expression:*** immune checkpoint blockade profoundly reshaped the expression landscape of co-inhibitory receptors and their ligands within the TME. PD-L1 expression on tumour cells was detected in several preclinical studies; some reported upregulation following treatment, while others observed a decrease, probably indicating differences in therapy and experimental context [43,68,70,75]. Conversely, some studies reported a decrease in PD-L1 expression after anti-PD-L1 therapy, likely reflecting receptor occupancy or target downregulation [68,70]. On infiltrating immune cells, PD-1 expression was commonly elevated on CD8^+^ and CD4^+^ T cells post-treatment, consistent with T-cell activation and the onset of exhaustion programmes [19,43,79].

The expression of alternative immune checkpoints, including **LAG-3**, T-cell immunoreceptor with Ig and ITIM domains **(TIGIT),** and **TIM-3,** was also markedly induced following CTLA-4 or PD-1/PD-L1 blockade. Upregulation of TIM-3 on both CD8^+^ T cells and Tregs was a recurring observation, potentially representing a mechanism of acquired resistance that could be targeted by combination strategies [19,43,79]. Similarly, increased LAG-3 and TIGIT expression on TILs was documented, further illustrating the complex network of compensatory inhibitory pathways engaged upon single-agent checkpoint inhibition [62,89].

##### Cytokine Profiles

***Intratumoral cytokine signatures:*** a pronounced shift toward a Th1-polarised cytokine milieu was a hallmark of effective immunotherapy in the B16-F10 model. Intratumoral levels of interferon-γ (IFN-γ) were consistently and significantly elevated following treatment with anti-CTLA-4, anti-PD-1, anti-PD-L1, and their combinations [39,40,43,62,74,78]. This increase in IFN-γ was often accompanied by concomitant elevations in tumour necrosis factor α (TNF-α) and interleukin 2 (IL-2), reinforcing the activation of cytotoxic T-lymphocyte responses [30,37,43,74]. The production of immunosuppressive T-helper 2 (Th2)-type cytokines, such as interleukin 4 (IL-4), interleukin 5 (IL-5), and interleukin 10 (IL-10), was more variably affected; some studies reported modest increases in IL-10 within the TME following monotherapy, while others demonstrated significant reductions, particularly with combination regimens or adjunctive agents [78,79,81]. The balance between effector and regulatory cytokines ultimately dictated therapeutic outcome, with a high IFN-γ/IL-10 ratio being associated with superior tumour control.

***Peripheral cytokine measurements:*** serum cytokine profiles largely mirrored intratumoral changes, with elevated circulating levels of IFN-γ, TNF-α, and IL-2 detected in treated mice [43,62,78]. Splenocytes from treated animals exhibited enhanced IFN-γ production upon ex vivo restimulation, confirming the systemic nature of the immune activation [37,66].

##### Chemokine Profiles

***Intratumoral chemokine expression:*** the recruitment of effector T cells into the TME was orchestrated, in part, by the upregulation of T-helper 1 (Th1)-type chemokines. Intratumoral expression of C-X-C motif chemokine ligand 9 (CXCL9) and C-X-C motif chemokine ligand 10 (CXCL10), ligands for the C-X-C motif chemokine receptor 3 (CXCR3) receptor expressed on activated CD8^+^ T cells and NK cells, was significantly increased following checkpoint blockade [25,43,62,74]. This chemokine induction was functionally linked to enhanced T-cell trafficking and correlated with the magnitude of CD8^+^ T-cell infiltration.

***Peripheral chemokine measurements:*** systemic levels of CXCL10 were also elevated in the serum of autoimmune regulator (Aire)-deficient treated mice, reflecting the broader inflammatory response and providing a potential peripheral biomarker of intratumoral immune activation [25].

##### Systemic/Peripheral Biomarkers

***Peripheral blood and lymphoid organ correlates:*** while the focus of this review is the intratumoral TME, several studies examined peripheral immune correlates that may inform on treatment response. As noted above, serum IFN-γ and CXCL10 levels were consistently elevated following effective immunotherapy, offering minimally invasive readouts of systemic immune activation [25,43,62]. Splenic immune cell composition was often altered, with increases in effector memory CD8^+^ T cells and activated NK cells, although these changes were generally less pronounced than those observed within the tumour [37,43,78]. Complete blood counts and serum chemistry panels were largely unremarkable, with no significant treatment-related toxicities reported in the majority of studies, underscoring the favorable safety profile of these agents in the immunocompetent B16F10 murine model [18,34,83].

***Tumour mutational burden:*** unlike the clinical setting, tumour mutational burden (TMB) was not a relevant biomarker in the B16-F10 model, as this cell line harbours a relatively low mutational load and lacks the neoantigen heterogeneity characteristic of human melanomas. Consequently, TMB was not assessed in any of the included preclinical studies.

#### 3.6.2. Clinical Arm

This narrative summary draws on all 44 clinical studies that assessed intratumoral TME parameters, whether or not they contributed to the quantitative meta-analysis. While the central emphasis remains on the intratumoral compartment, peripheral blood biomarkers are again noted briefly for context, as they offer additional information on systemic immune responses that can, indirectly, mirror processes unfolding within the tumour microenvironment.

##### Spatial and Stromal Architecture

In post-treatment specimens from the OpACIN-neo trial, the percentage area of fibrosis/fibroinflammatory stroma within the tumour bed was significantly higher in pathological responders than in non-responders (*p* < 0.001) [49]. A 10% increase in fibrosis area was associated with lack of recurrence (odds ratio (OR) 0.94, 95% CI 0.88–0.97, *p* = 0.008) and hyalinized fibrosis, particularly correlated with favorable outcome (OR 0.89, *p* = 0.025). All pathological non-responders who recurred had ≥70% viable tumour. An immune-related pathologic response score based on hematoxylin and eosin (H&E)-stained biopsies identified colocalised neovascularization and proliferative fibrosis as features associated with response (*p* = 0.0006) [127]. After a single neoadjuvant dose of anti-PD-1, tumour necrosis increased significantly (median from 0% to 30%, *p* = 0.007), and higher necrosis was associated with major pathological response (*p* = 0.001) [125]. In a phase II trial of pembrolizumab for desmoplastic melanoma, 73% of on-therapy biopsies showed treatment-related fibrosis and immune-mediated regression, including 46% with complete pathological response [120]. Single-nucleus RNA-seq identified an inflammatory cancer-associated fibroblast iCAF_CCL21 fibroblast subgroup, absent in pre-treatment tumours of non-responders that expressed C-C motif chemokine ligand 21 (CCL21), and was proposed to recruit immune cells via the C-X-C motif chemokine ligand 12 (CXCL12)-C-X-C motif chemokine receptor 4 (CXCR4) axis [51].

##### Tumour Cell Features

A single dose of neoadjuvant pembrolizumab reduced the percentage of viable tumour, with 29.6% of patients achieving complete or major pathological response (≤10% viable tumour); all such patients remained disease-free [50]. Baseline apoptotic debris was more frequent in responders (*p* = 0.008), and a higher mitotic rate correlated with greater radiological tumour shrinkage (r = 0.2238, *p* = 0.0408) [45]. Analyzing paired biopsies from CheckMate 038, responding tumours exhibited IFN-γ-induced dedifferentiation (downregulation of MITF/MLANA and upregulation of AXL) on therapy; baseline enrichment of this dedifferentiation signature tended to be higher in responders (*p* = 0.06) and correlated with improved overall survival in The Cancer Genome Atlas (TCGA) [121]. In the tremelimumab study, degenerative changes in melanoma cells were observed in responders, and one complete responder had no residual tumour cells post-treatment, only lymphocytes and scar tissue [48].

##### Cytotoxic Immune Cells

***Intratumoral findings:*** higher baseline intratumoral CD8^+^ T-cell density consistently predicted favorable outcomes. The landmark pembrolizumab study demonstrated that CD8^+^ density at the invasive margin and tumour centre was significantly higher in responders (*p* < 0.0001), and a predictive model based on invasive margin CD8^+^ density was validated in an independent cohort [47]. Using multiplex IHC and mass cytometry (CyTOF), pre-treatment CD8^+^ densities were higher in responders to both anti-PD-1 monotherapy and anti-PD-1/anti-CTLA-4 combination (*p* < 0.05), with an eomesodermin (EOMES)^+^,cluster of differentiation 69 (CD69, an early activation marker)^+^,cluster of differentiation 45RO (CD45RO, a memory T-cell marker)^+^, and effector memory CD8^+^ T-cell phenotype enriched in responders to combination therapy (*p* < 0.05) [107]. Data from the adjuvant CheckMate 915 analysis showed that high CD8^+^ T-cell infiltration was associated with longer recurrence-free survival (RFS) in both nivolumab (hazard ratio (HR) 0.77) and nivolumab + ipilimumab (HR 0.71) arms [46]. In the neoadjuvant setting, the Morpheus trial found that baseline CD8^+^ density in tumour nests and stroma correlated with major pathological response (*p* < 0.05) [55]. Most responders from a phase II pembrolizumab trial for brain metastases had higher pre-treatment CD8^+^ TIL density, whereas all non-responders had low CD8^+^ TILs [122]. A post-treatment increase in TILs (by H&E) was associated with clinical benefit (*p* = 0.005; OR 13.27) in an ipilimumab trial, though baseline CD8^+^ IHC was not significant [101]. Findings from a sequential checkpoint blockade trial showed that CD8^+^ T-cell counts correlated with T-cell fraction (Pearson correlation coefficient, r = 0.784) and clonality (r = 0.418), and a composite biomarker including T-cell fraction and clonality predicted response in the nivolumab-then-ipilimumab arm (receiver operating characteristic area under the curve (ROC AUC) ~0.70) [103]. An ipilimumab cohort demonstrated that pre-treatment CD8A mRNA expression was 2.8-fold higher in patients with clinical activity (*p* = 5.1 × 10^−4^) [119]. The 4-gene inflammatory signature (including CD8A) was associated with longer progression-free survival (PFS) and overall survival (OS), and combined high TMB and high CD8 IHC yielded a higher objective response rate (ORR) in CheckMate 067 [102].

**On-treatment expansion** and infiltration of CD8^+^ T cells were also documented. Serial biopsies from responders showed a parallel increase in CD8^+^ density at the invasive margin and tumour centre, correlating with radiological tumour size reduction (r = −0.75, *p* = 0.0002), and increased CD8^+^ Ki67^+^ cells in the tumour parenchyma and granzyme B expression on CD8^+^ cells post-treatment (*p* < 0.0001) [47]. After one neoadjuvant pembrolizumab dose, CD8^+^ T-cell infiltration increased, and most tumour CD8^+^ TILs showed an exhausted phenotype (PD-1^+^Tim-3^+^CTLA-4^+^LAG-3^+^TIGIT^+^cluster of differentiation 39 (CD39)^+^, Eomes^hi^ T-box transcription factor (Tbet)^lo^); gp100-specific CD8^+^ T cells rose post-treatment (n = 2) [50]. In the OpACIN-neo trial, higher CD8^+^ T-cell density (flow cytometry) was noted in pathological responders [49]. On-treatment increases in CD8^+^ T cells, CD8^+^Ki67^+^ cells, and CD3^+^Perforin^+^ cells were observed in the nivolumab + ipilimumab arm of the Morpheus trial [55]. In the IMPemBra trial, CD8^+^ T-cell percentage in tumour tissue increased from baseline to week 6 in the pembrolizumab monotherapy cohort [105]. In a lead-in nivolumab trial, intratumoral CD8^+^ density significantly increased after nivolumab (*p* = 0.01), and major pathological response on biopsy correlated with higher CD8^+^ density [53]. In the tremelimumab study, post-treatment intratumoral CD8^+^ density increased from 289 to 955 cells/mm^2^ (*p* = 0.005), irrespective of clinical response [48]. Early during anti-PD-1 therapy, total CD8^+^ T cells and cluster of differentiation 103 (CD103, integrin αE)^+^CD8^+^ tumour-resident T cells expanded; the expansion of CD103^+^ CD8^+^ cells tended to be greater in responders (*p* = 0.07) [114]. Peritumoral CD3^+^ density was significantly higher in responders (*p* = 0.041) [45]. Single-cell RNA-seq identified a high-oxidative phosphorylation (OXPHOS) CD8^+^ T-cell subset (CD8^+^_TOXPHOS) that co-expressed cytotoxic markers (perforin 1 (PRF1), granzyme B (GZMB), IFN-γ) and exhaustion markers and was enriched in non-responders, suggesting a resistance-associated state [124].

***Peripheral blood CD8^+^ T cell and NK cell dynamics:*** although not the focus of this review, several studies reported circulating immune changes. In peripheral blood, CD8^+^ T effector memory (TEM) cells, identified by the combined phenotype CD45RO^+^ (memory marker), C-C motif chemokine receptor 7 (CCR7^−^) (absent lymph node homing receptor), cluster of differentiation 27 (CD27^−^) (absent costimulatory molecule), and cluster of differentiation 57 (CD57^−^) (absent terminal differentiation marker) (CD8^+^CD45RO^+^CCR7^−^CD27^−^CD57^−^), increased in frequency in responders (*p* = 0.006) [108]. The proportion of CTLA-4^hi^PD-1^hi^ CD8^+^ T cells in pre-treatment tumour correlated with response; these cells produced IFN-γ but reduced TNF-α and IL-2 (partially exhausted) [52]. In blood, a robust increase in Ki67^+^ CD8^+^ T cells was observed as early as day 7, with responding cells being PD-1^+^ and enriched for PD-1^+^CTLA-4^+^ [50]. Circulating CD8^+^ mucosal-associated invariant T (MAIT) cell frequency increased on treatment in responders (*p* < 0.01), and high levels were associated with improved overall survival [130]. In a prospective cohort, absolute lymphocyte count at 3 months was higher in responders (1.9 vs. 1.2 × 10^9^/L, *p* = 0.04); older age, lower naïve CD8^+^ T-cell frequency, and higher serum monocyte chemotactic protein 4 (MCP-4)/osteoprotegerin (OPG) were baseline predictors of response [106]. In patients developing vitiligo, PD-1 expression decreased on most T-cell subsets after anti-PD-1 but increased on NK cluster of differentiation 56 (CD56)^bright^ cells [110]. In a nivolumab lead-in arm, CD8^+^ T-cell gene signatures for terminal exhaustion and IFN-γ response were enriched, but phosphorylated SH2 domain-containing leukocyte protein (pSLP76, TCR signaling) in blood did not change, nor did the frequency of cluster of differentiation 38 (CD38^+^) TIM3^+^ CD8^+^ T cells [111]. Natural killer T (NKT) cell frequency increased in responders (*p* = 0.01), and NK cell activation markers (cluster of differentiation 25 (CD25, IL-2 receptor α chain) and CD45RO) also increased (*p* = 0.03 and 0.04, respectively) [106]. In the desmoplastic melanoma trial, pre-existing CD8^+^ T cells were noted by IHC but not quantified [120].

##### Regulatory/Suppressive Cells

***Intratumoral findings:*** regulatory T-cell (FOXP3^+^) data were heterogeneous. Intratumoral FOXP3^+^ densities were higher in responders to combined immunotherapy at early on-treatment (*p* < 0.05) [107]. A neoadjuvant pembrolizumab study found that Treg proliferation (Ki67^+^FOXP3^+^) increased in tumour and blood after anti-PD-1, and high Treg proliferation was associated with recurrence and poor disease-free survival (DFS); increased cluster of differentiation 163 (CD163^+^) myeloid cells were also seen in recurrent tumours [50]. Data from the tremelimumab study showed that FOXP3^+^ cell density increased post-treatment (from 35.2 to 167.4 cells/mm^2^, *p* = 0.0029), with a non-significant trend toward higher density in responders (994 vs. 776 cells/mm^2^, *p* = 0.33) [48]. An ipilimumab phase II trial paradoxically reported that high baseline FOXP3^+^ expression was associated with clinical benefit (*p* = 0.014; OR 10.38) [101]. One anti-PD-1 study observed that high FOXP3^+^ TILs (>10 cells/high-power field (HPF)) were a negative predictor of progression-free survival (PFS) (HR3.04, *p* = 0.048) [116]. FOXP3 mRNA was not significantly different, but the CD8/FOXP3 ratio was higher in responders (*p* = 0.04) [118].

Regarding **myeloid cells**, monocytic MDSCs (cluster of differentiation14 (CD14)^+^ human leukocyte antigen-DR Isotype (HLA-DR)^low^) increased on therapy irrespective of response (*p* = 0.04) [108]. High cluster of differentiation68 (CD68^+^) TAMs were associated with shorter PFS (HR 3.21, *p* = 0.034), and co-expression of FOXP3 and CD68 identified a very high-risk group (HR3.97, *p* = 0.008) [116]. A separate investigation noted that peritumoral CD68^+^ macrophage density early during treatment was significantly higher in responders (389 vs. 196, *p* = 0.017) and macrophage PD-L1 immunoreactive score (IRS) was also higher in responders (150 vs. 46, *p* = 0.033) [45].

***Peripheral blood findings:*** in an adjuvant ipilimumab analysis, high circulating CTLA-4^+^ Tregs and monocytic MDSCs correlated with worse overall survival (OS) and recurrence-free survival (RFS), whereas CD39^+^ Tregs were associated with better outcomes [100]. In an anti-PD-1 study, Tregs were not a focus [106]. Patients developing vitiligo had reduced circulating Treg and T-helper 17 (Th17) cells [110].

##### Checkpoint Molecule Expression

***Intratumoral PD-L1 expression and PD-1/PD-L1 proximity:*** higher baseline PD-L1 expression on tumour or immune cells was consistently associated with response. Pre-treatment PD-L1^+^ cell density was higher in responders (*p* = 0.006), and PD-1/PD-L1 proximity correlated with response (*p* = 0.005) [47]. Intratumoral PD-1 and PD-L1 densities were significantly higher in responders to both monotherapy and combination therapy (*p* < 0.05) [107]. High tumour cell PD-L1 expression was associated with longer recurrence-free survival (RFS) in the adjuvant CheckMate 915 analysis (HR 0.80 for nivolumab, 0.77 for combination) [46]. Findings from the Morpheus trial indicated that baseline PD-L1 expression was higher in the nivolumab + ipilimumab arm than in the tobemstomig (anti-PD-1/LAG-3 bispecific antibody) arm, and baseline PD-L1 was associated with major pathological response (*p* < 0.05) [55]; these data were not included in the meta-analysis because no comparator group was available. PD-L1 expression on tumour cells increased after treatment in the neoadjuvant pembrolizumab study (*p* < 0.01) [50]. A phase I pembrolizumab trial, applying a melanoma PD-L1 expression (MEL) score (0–5), reported that 76% of tumours were PD-L1^+^ (MEL ≥ 2); higher MEL scores correlated with higher ORR (8% to 57%) longer PFS (HR 0.51) and OS (HR 0.50) [112]. Tumour PD-L1 IRS early during treatment was significantly higher in responders (49 vs. 4, *p* = 0.025), and macrophage PD-L1 IRS also increased in responders (150 vs. 46, *p* = 0.033) [45]. PD-L1 expression (MEL score) was higher in responders, although the proximity ligation assay outperformed PD-L1 IHC [44]. Low PD-L1 protein expression by digital spatial profiling was associated with relapse in the OpACIN trial [54]. Pre-treatment PD-L1 mRNA was higher in responders (*p* = 0.03), and a composite score of PD-L1, granzyme A (GZMA), and human leukocyte antigen-A (HLA-A) distinguished responders (*p* = 0.0016) [118]. A separate anti-PD-1 study noted that 94.7% of metastatic site biopsies were PD-L1^+^ (>1%), but PD-L1 expression did not correlate with outcome; instead, high tumour galectin-3 (Gal-3) was associated with progression [115]. The CD8^+^_TOXPHOS subset, identified by single-cell RNA-seq, highly expressed PD-1, TIM-3, LAG-3, CTLA-4, TIGIT, and CXCL13 [124]. In a nivolumab lead-in arm, enrichment of exhaustion and IFN-γ response gene signatures was noted, but quantitative checkpoint protein data were not provided [111].

***Intratumoral PD-1 expression on T cells:*** higher pre-treatment PD-1^+^ cell density in responders (*p* = 0.0002) [47], higher PD-1 expression on T cells in responders [50,107], and an increased proportion of PD-1^+^CTLA-4^+^ CD8^+^ T cells in tumour post-treatment [50] were reported. CTLA-4^hi^PD-1^hi^ CD8^+^ T cells were identified as a predictive subset [52]. PD-1 expression on CD8^−^ CD3^+^ T cells was higher in responders (55.6% vs. healthy 34.2%, *p* = 0.01) [106]. Tumour-infiltrating MAIT cells had increased PD-1 expression compared to circulating cells [130]. PD-1 expression on CD4^+^ T cells decreased after treatment (*p* < 0.05) [115]. In patients developing vitiligo, PD-1 expression decreased on most T-cell subsets after anti-PD-1 but increased on NK CD56^bright^ cells [110].

***Intratumoral co-inhibitory receptors*** *CTLA-4*, *LAG-3*, *TIGIT*, and *TIM-3*: upregulation of LAG-3, TIGIT, and other checkpoint genes in responders was demonstrated by RNA-seq [107]. Baseline LAG-3 protein expression was associated with major pathological response (MPR) (*p* < 0.05) [55]. CD8^+^ TIL expressed LAG-3 and TIGIT [50]. The CD8^+^_TOXPHOS subset co-expressed LAG-3, TIM-3, and TIGIT [124]. Gal-3 (a lectin) hindered pembrolizumab binding in vitro [115].

##### Cytokine Profiles

***Intratumoral cytokine signatures:*** higher pre-treatment expression of IFN-γ-inducible genes was observed in responders [119]. Moreover, upregulation of IFN-γ, tumour necrosis factor (TNF), IL-2, and other cytokine genes in responders was found by RNA-seq [107]. Furthermore, a high IFN-γ RNA signature score was correlated with longer RFS (HR 0.60 for nivolumab, 0.63 for combination) [46]. Similarly, the IFN-γ gene signature was also linked to MPR at baseline (*p* < 0.05) and increased on treatment (*p* < 0.01) [55]. Consistently, an increase in the IFN-γ signature was observed after pembrolizumab monotherapy [105]. Notably, phosphorylated signal transducer and activator of transcription 1 (pSTAT1), a downstream mediator of IFN-γ, was higher in responders at baseline (*p* = 0.002) and during treatment (*p* < 0.0001) [47,50].

***Peripheral blood cytokine measurements:*** additionally, serum interleukin-12B (IL-12B) and tumour necrosis factor receptor superfamily member 9 (TNFRSF9) increased during therapy, particularly in responders (*p* = 0.01 and 0.02) [106]. Serum interleukin-8 (IL-8), also known as C-X-C motif chemokine ligand 8 (CXCL8), changes predicted response [126]. In contrast, stimulated B cells from non-responders produced more tumour necrosis factor alpha (TNF-α) and interleukin-6 (IL-6) [113]. Moreover, increased interleukin-17A (IL-17A)^+^ cells were noted in vitiligo lesions [110]. Finally, plasma Gal-3 and soluble programmed cell death protein 1 (sPD-1) were measured [115], but not classical cytokines. Taken together, the intratumoral and peripheral cytokine profiles consistently pointed to an IFN-γ-driven, Th1-polarised immune activation in responders, accompanied by a selective rise in soluble immune mediators in the circulation, most notably elevated IL-12B and TNFRSF9 in responders and dynamic changes in serum IL-8.

##### Chemokine Profiles

***Intratumoral chemokine expression:*** higher baseline levels of CXCL9 (3.0-fold), CXCL10 (3.5-fold), C-X-C motif chemokine ligand 11 (CXCL11) (3.0-fold), C-C motif chemokine ligand 4 (CCL4) (2.5-fold), and C-C motif chemokine ligand 5 (CCL5) (2.7-fold) were observed in responders, together with a post-treatment increase of CXCL11 (4.0-fold; all *p* < 0.01) [119]. Transcriptomic data also showed upregulation of CXCL13, CCL5, CCL4, CXCL9, and C-C motif chemokine receptor 5 (CCR5) in responders [107]. ICAF_CCL21 fibroblasts expressed CCL21, and the CXCL12-CXCR4 axis recruited B cells [51]. Moreover, high tumour expression of CXCL9, CXCL10, and CXCL11 was associated with better survival [100].

***Peripheral blood chemokine measurements:*** In the circulation, serum CXCL9, CXCL10, and CXCL11 increased during treatment in responders (*p* = 0.001, 0.03, and 0.01, respectively), and CXCR3 expression on CD8^−^ T cells correlated with CXCL9 (r = 0.58, *p* = 0.02) [106]. Consistently, early on-treatment changes in serum IL-8 predicted response: a > 9.2% increase was associated with non-response; in responders, serum IL-8 decreased [126]. Additionally, elevated serum C-C motif chemokine ligand 3 (CCL3) and CXCL11 were associated with improved survival [100]. Furthermore, plasma CXCL9 and CXCL10 increased post-treatment irrespective of response, and high baseline C-X3-C motif chemokine ligand 1 (CX3CL1) was associated with non-response (*p* = 0.0020) [104].

##### Systemic/Peripheral Biomarkers

***Circulating tumour DNA (ctDNA)*:** baseline ctDNA positivity (16.2% of patients) corresponded to a higher risk of recurrence (HR 1.97 for RFS, 2.86 for distant metastasis-free survival (DMFS)) in the adjuvant CheckMate 915 trial, whereas on-treatment ctDNA clearance (“zero conversion”) indicated the best outcome [46].

***Serum lactate dehydrogenase (LDH) and other routine markers*:** elevated LDH was present in 39% of patients in a phase II pembrolizumab trial [122] and was linked to poor outcome in another study [45]. Similarly, elevated C-reactive protein (CRP) correlated with FOXP3^+^ TILs (*p* = 0.024), while LDH showed no significant relationship with survival [116]. Furthermore, absolute lymphocyte count and neutrophil frequency were prognostic for outcome [106]. In a further analysis, LDH was integrated into a risk score but not highlighted alone [100], whereas all patients had normal LDH at baseline in the OpACIN trial (inclusion criterion) [54].

***Tumour mutational burden (TMB) and neoantigens*:** high TMB (>median) conferred improved PFS and OS across treatment arms in CheckMate 067 [102]. In the same vein, in the adjuvant CheckMate 915 study, high TMB (≥350 mutations/tumour) predicted longer RFS (HR 0.72 for nivolumab, 0.67 for combination) [46]. Consistently, high TMB was a predictor of response in the neoadjuvant OpACIN-neo study (median 860 vs. 293, *p* = 0.0013) [104]. In the desmoplastic melanoma trial, the median TMB reached 82.1 mutations per megabase (Mut/Mb), with frequent NF1 mutations [120]. Beyond overall TMB, expressed mutation burden out performed TMB alone in predicting OS [109]. Finally, mutational load displayed a marginal correlation with response in the nivolumab-then-ipilimumab arm (*p* = 0.06) [103]. Collectively, the analysed biomarkers included ctDNA clearance, LDH, CRP, absolute lymphocyte count, neutrophil frequency, TMB, and expressed mutation burden, each evaluated for their association with clinical outcome across multiple cohorts.

Studies that reported only peripheral blood biomarkers without corresponding intratumoral TME data were excluded from the present synthesis, as the primary focus of this review is the intratumoral microenvironment. Nevertheless, a comprehensive list of such peripheral biomarker studies is provided in Supplementary Table S4 [132,133,134,135,136,137,138,139,140,141,142,143,144,145,146,147,148,149,150,151,152,153,154,155,156,157,158,159,160,161,162,163,164,165,166,167,168,169,170,171,172,173,174,175,176,177,178,179].

## 4. Discussion

### 4.1. Principal Findings

This dual systematic review synthesized evidence from 58 preclinical studies (of which 46 contributed extractable quantitative data for the meta-analysis, yielding 99 data points) and 44 clinical studies (of which 19 contributed extractable quantitative data for the meta-analysis, yielding 44 data points). The integrated analysis comprehensively maps the reprogramming of the melanoma tumour microenvironment by immune checkpoint blockade. The principal findings are unequivocal: immunotherapy consistently and profoundly remodels the TME toward an immune-activated, proinflammatory, and tumour-suppressive state. In the preclinical B16F10 model, immune checkpoint blockade significantly increased intratumoral CD8^+^ T-cell infiltration (pooled SMD = 1.45, 95% CI 1.05 to 1.85, *p* < 0.001), IFN-γ production (pooled SMD = 1.78, 95% CI 0.95 to 2.62, *p* < 0.001), the CD8/Treg ratio (pooled SMD = 0.91, 95% CI 0.28 to 1.55, *p* = 0.005), and apoptosis (pooled SMD = 3.54, 95% CI 2.13 to 4.95, *p* < 0.001), while significantly reducing Ki-67 proliferation (pooled SMD = −1.43, 95% CI −2.70 to −0.15, *p* = 0.028) and PD-L1 expression (pooled SMD = −0.88, 95% CI −1.48 to −0.28, *p* = 0.004). The clinical synthesis confirmed the robust increase in CD8^+^ T-cell infiltration (pooled SMD = 0.72, *p* < 0.001) and, in contrast to the preclinical findings, demonstrated a significant increase in PD-L1 expression (pooled SMD = 0.67, *p* = 0.001), together with a borderline increase in IFN-γ signatures (pooled SMD = 0.59, *p* = 0.061). Sensitivity analyses excluding low-n and high-ROB studies confirmed the robustness of these core findings, while formal assessment of publication bias revealed no compelling evidence of small-study effects for the primary outcomes. Collectively, these data establish that immune checkpoint blockade engages a **conserved and multi-faceted TME remodeling programme** across species, albeit with notable divergences in magnitude and specific mechanistic features.

### 4.2. Comparison of Preclinical and Clinical TME Reprogramming

**Throughout this comparison,** it is important to distinguish between the treatment effects observed in the controlled B16F10 preclinical model and the clinical associations derived from heterogeneous patient cohorts; the two arms provide complementary but not equivalent levels of mechanistic inference.

**CD8^+^ T-cell Infiltration:** the most robust and translationally concordant finding across both arms of the review was the increase in intratumoral CD8^+^ T-cell abundance. In the B16F10 preclinical corpus, this effect was documented in 41 data points from 39 studies and was independent of the specific checkpoint inhibitor employed [17,18,19,21,25,37,43,77]. The pooled effect magnitude was substantial, corresponding to a greater than four-fold geometric mean increase. In the clinical literature, higher baseline CD8^+^ density consistently predicted favorable outcomes, and on-treatment expansion of CD8^+^ T cells was documented across multiple trials [46,47,50,105,107]. The convergence of these findings across species underscores the centrality of cytotoxic T-lymphocyte recruitment and proliferation as the canonical mechanism of checkpoint inhibitor efficacy.

**CD8/Treg Ratio:** the preclinical synthesis demonstrated a significant increase in the CD8/Treg ratio (pooled SMD = 0.91, *p* = 0.005), driven by both CD8^+^ T-cell expansion and, in many cases, a reduction in FoxP3^+^ regulatory T-cell frequencies [21,25,43,78]. Among clinical cohorts, the CD8/Treg ratio was not significantly altered in the pooled analysis (SMD = −0.21, *p* = 0.649), and individual studies reported heterogeneous and sometimes paradoxical findings, including increases in Treg density following therapy [48]. The discordance may stem from the greater complexity and plasticity of the human Treg compartment. The preclinical data suggest that combination regimens or adjunctive agents may be required to durably suppress Treg-mediated immunosuppression.

**PD-L1 Expression, a Critical Divergence:** a striking and mechanistically informative divergence emerged in PD-L1 dynamics. In the clinical setting, higher baseline PD-L1 expression and on-treatment upregulation were consistently associated with response [45,47,107,112], reflecting the phenomenon of adaptive immune resistance wherein IFN-γ-driven PD-L1 induction serves as a brake on unrestrained T-cell activity. In stark contrast, the preclinical meta-analysis revealed a significant decrease in intratumoral PD-L1 expression following checkpoint blockade (pooled SMD = −0.88, *p* = 0.004), an effect that was remarkably homogeneous across the **four data points from three studies** contributing to this outcome (I^2^ = 0%) [43,70,84]. While the consistency of this effect across the available studies is noteworthy, the small number of data points (four) necessarily limits the precision of the pooled estimate and the generalisability of this finding; the observed preclinical decrease in PD-L1 should therefore be interpreted as a preliminary signal that warrants replication in independent datasets. This discrepancy may reflect fundamental differences in tumour immunobiology; the B16-F10 model, with its relatively low mutational burden and poor intrinsic immunogenicity, may fail to mount the robust IFN-γ response required to drive adaptive PD-L1 upregulation. Alternatively, the kinetics of PD-L1 modulation may differ, with preclinical assessments often performed at early time points prior to the establishment of full adaptive resistance. This divergence cautions against over-interpreting PD-L1 dynamics in the B16-F10 model.

**IFN-γ Production:** both preclinical and clinical syntheses demonstrated elevated intratumoral IFN-γ signaling following therapy. In murine tumours, IFN-γ levels were markedly increased, with the largest effects observed in anti-PD-L1-containing regimens [39,40,43,74,78]. Clinically, IFN-γ gene signatures were enriched in responders and associated with longer recurrence-free survival [46,105,107,119]. The slightly weaker pooled effect in the clinical meta-analysis (SMD = 0.59, *p* = 0.061) likely reflects the greater heterogeneity of human tumours.

**Apoptosis and Proliferation:** the preclinical literature provided rich, quantitative data on terminal tumour cell fate, demonstrating both a profound induction of apoptosis (pooled SMD = 3.54, *p* < 0.001) and a significant suppression of Ki-67-defined proliferation (pooled SMD = −1.43, *p* = 0.028) [17,18,27,32,36,39,64,78]. These effects were particularly pronounced with combination regimens. In the clinical literature, apoptosis and proliferation data were sparse; only one study reported quantitative apoptotic debris counts [45], and Ki-67 data were limited to three studies with no significant pooled effect. This single clinical data point precludes any meta-analytic inference on apoptosis in patients and highlights a significant gap in the clinical biomarker literature that future studies should urgently address. This asymmetry underscores the value of the B16F10 preclinical corpus in illuminating terminal effector consequences that remain largely opaque in human studies.

**Spatial and Stromal Architecture:** the narrative synthesis revealed a notable gap in the preclinical characterization of spatial and stromal remodeling. While clinical studies documented treatment-related fibrosis, necrosis, and the emergence of immunomodulatory fibroblast subsets [49,51,125,127], the preclinical literature largely confined stromal assessments to quantification of CD31^+^ microvascular density, which was consistently reduced following therapy [17,18,90]. The absence of detailed spatial and architectural analyses in the B16F10 preclinical literature represents an important limitation and an opportunity for future investigation.

**Alternative Checkpoint Upregulation (Adaptive Resistance):** beyond PD-L1 dynamics, the narrative synthesis revealed that checkpoint blockade consistently upregulates a network of alternative co-inhibitory receptors on intratumoral T cells. In the B16F10 model, increased expression of TIM-3, LAG-3, and TIGIT was documented on both CD8^+^ effector cells and FoxP3^+^ regulatory T cells following anti-PD-1 or anti-CTLA-4 therapy [19,43,79]. In human tumours, single-cell analyses identified a CD8^+^ T-cell subset (CD8^+^_TOXPHOS) co-expressing these exhaustion markers that was enriched in non-responders [124]. This conserved transcriptional programme of adaptive resistance may undermine the durability of single-agent checkpoint blockade and underscores the therapeutic rationale for dual or sequential targeting of these parallel inhibitory pathways.

These observations support prioritising PD-L1-based combinations to amplify effector functions, highlight CD8^+^ infiltration and PD-L1 expression as actionable biomarkers for patient stratification and monitoring, and directly inform the design of next-generation immunotherapy trials.

### 4.3. Interpretation of Heterogeneity

Substantial heterogeneity was observed across nearly all outcomes in both the preclinical (I^2^ = 68–88%) and clinical (I^2^ = 60–84%) meta-analyses. This heterogeneity is not a statistical nuisance but rather a faithful reflection of the methodological and biological diversity inherent to the included studies. In the preclinical arm, the use of the standardized mean difference (SMD) harmonized disparate measurement scales, ranging from cells/mm^3^ and cells/field to cells/g and percentages of CD45^+^ leukocytes, yet residual heterogeneity persisted, driven by variations in dosing schedules, antibody clones, tumour sampling time points (day 7 to day 21), and analytical platforms (flow cytometry versus immunohistochemistry). Notably, this residual heterogeneity persisted despite the deliberate restriction to a single inbred mouse strain (C57BL/6), the exclusive use of unmodified, parental B16-F10 cells, a uniform subcutaneous implantation route, and the sole use of intraperitoneal drug administration, a level of methodological standardization that underscores the genuine biological and procedural diversity captured by the meta-analysis. In the clinical arm, heterogeneity was similarly multifactorial, with comparator type, treatment line, and assay methodology all contributing. Critically, sensitivity analyses excluding low-n and high-ROB studies did not appreciably reduce I^2^ for most outcomes, with the notable exception of CD8/Treg in the clinical arm, where exclusion of high-ROB studies reduced I^2^ from 84% to 26%, confirming that the observed variation is largely attributable to true differences in experimental conditions and tumour biology rather than to methodological flaws. Nevertheless, for outcomes with very small numbers of studies, particularly preclinical PD-L1, the pooled estimates should be regarded as provisional, and their interpretive confidence is necessarily constrained until additional data become available. The random-effects model employed throughout appropriately accounts for this heterogeneity, yielding pooled estimates that represent the average effect across a distribution of true effects, rather than assuming a single common effect.

### 4.4. Pharmacological Implications of Drug Class Moderation

Meta-regression analyses in the preclinical arm revealed that drug class significantly moderated the effects on IFN-γ production (***p* = 0.029**), the CD8/Treg ratio (***p* = 0.008**), and apoptosis (***p* = 0.016**). Notably, in the preclinical B16F10 analysis, anti-PD-L1-containing regimens, whether as monotherapy or in combination with anti-CTLA-4, consistently produced the largest effect sizes. Relative to anti-CTLA-4 monotherapy, anti-PD-L1 treatment was associated with substantially greater increases in IFN-γ (SMD difference = +3.59, ***p* = 0.009**), the CD8/Treg ratio (SMD difference = +10.69, ***p* = 0.003**), and apoptosis (SMD difference = +9.76, ***p* = 0.004**), as well as a greater reduction in Ki-67 proliferation (SMD difference = −6.28, ***p* = 0.040**). These findings align with the distinct biology of the PD-1/PD-L1 axis, which primarily regulates effector T-cell function within peripheral tissues, and suggest that PD-L1 blockade may be particularly adept at reinvigorating exhausted CD8^+^ T cells and shifting the intratumoral CD8/Treg balance. The particular potency of PD-L1 blockade likely reflects its dual ability to reinvigorate exhausted CD8^+^ T cells and reverse PD-L1-mediated immunosuppressive myeloid programming, as illustrated in Figure 1B, whereas CTLA-4 blockade acts predominantly during T-cell priming in lymphoid organs and through Fc-mediated Treg depletion (Figure 1A). The lack of significant moderation for CD8^+^ T-cell infiltration **(*p* = 0.414)** indicates that all checkpoint inhibitor classes are capable of promoting T-cell recruitment but that the downstream functional consequences, cytokine elaboration, effector to suppressor balance, and tumour cell killing, are more potently engaged by PD-L1-targeted therapies. In the clinical arm, drug class did not significantly moderate any outcome, likely reflecting the smaller number of studies and the predominance of anti-PD-1 monotherapy in the clinical corpus, which limited power to detect differential effects. These results underscore the superior potency of PD-L1 blockade in the B16F10 model.

**Figure 12:**
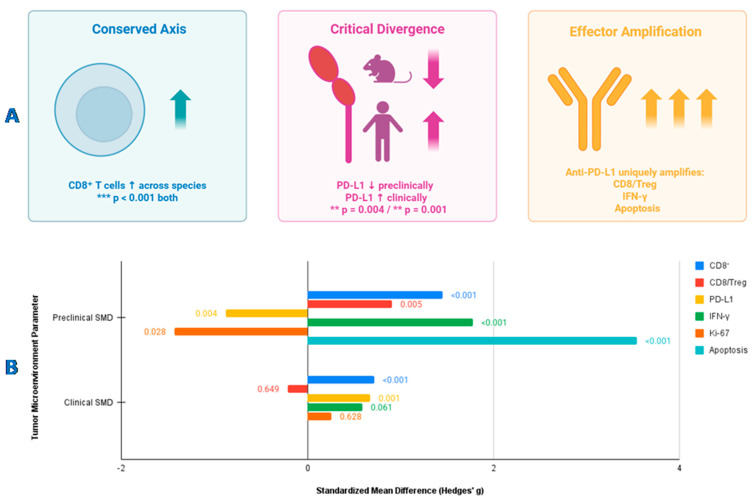
**Conceptual Synthesis and Quantitative Meta-analysis of Melanoma TME Reprogramming by Immune Checkpoint Blockade.** (**A**) Three Pillars of TME Reprogramming. **Left**: Conserved Axis. CD8^+^ T-cell infiltration increases significantly and concordantly across preclinical (B16F10) and clinical cohorts (both *p* < 0.001). Middle: Critical Divergence. PD-L1 expression decreases preclinically (*p* = 0.004) but increases clinically (*p* = 0.001), reflecting species-specific adaptive resistance. **Right**: Effector Amplification. Anti-PD-L1-containing regimens uniquely amplify effector functions relative to anti-CTLA-4, with substantial SMD differences in IFN-γ (+3.59), CD8/Treg ratio (+10.69), and apoptosis (+9.76). (**B**) Pooled Standardized Mean Differences (SMDs) for TME Remodeling. Bar chart displaying pooled SMDs (Hedges’ *g*) from preclinical (B16F10) and clinical meta-analyses. Values on bars represent exact *p*-values. Preclinical SMDs: CD8^+^ +1.45 (*p* < 0.001), CD8/Treg +0.91 (*p* = 0.005), PD-L1 −0.88 (*p* = 0.004), IFN-γ +1.78 (*p* < 0.001), Ki-67 −1.43 (*p* = 0.028), Apoptosis +3.54 (*p* < 0.001). Clinical SMDs: CD8^+^ +0.72 (*p* < 0.001), CD8/Treg −0.21 (*p* = 0.649), PD-L1 +0.67 (*p* = 0.001), IFN-γ +0.59 (*p* = 0.061), Ki-67 +0.26 (*p* = 0.628). Apoptosis was reported in only one clinical study and could not be pooled quantitatively. Created with BioRender.com and sheets.google.com.

These core pharmacological findings are visually synthesised in Figure 12, which presents a unified conceptual framework alongside the underlying quantitative evidence. Panel A integrates the three central messages of the review, conserved CD8^+^ infiltration, divergent PD-L1 dynamics, and superior effector amplification by PD-L1 blockade, into a single schematic, while Panel B provides the corresponding pooled SMDs for all six TME parameters as a bar chart, allowing direct visual comparison of effect magnitudes and directions across the preclinical and clinical arms.

### 4.5. Certainty of Evidence

A formal assessment of the certainty of evidence was conducted using the GRADE (Grading of Recommendations, Assessment, Development and Evaluations) framework [61] for all primary outcomes. The certainty of evidence was rated as Low for the majority of TME parameters, primarily reflecting the incomplete reporting of methodological safeguards in the primary literature rather than any imprecision or inconsistency in the pooled effect estimates. The primary factor contributing to these ratings was the serious risk of bias arising from incomplete reporting of methodological safeguards, specifically, randomization, allocation concealment, and blinding, across the included primary studies. Of note, sensitivity analyses excluding studies with an explicitly high risk of bias did not alter the direction, magnitude, or statistical significance of any pooled estimate, confirming the robustness of the findings. Furthermore, the observed biological effects were substantial in magnitude, including a 34-fold geometric mean increase in preclinical apoptosis (SMD = 3.54) and a greater than four-fold increase in CD8^+^ T-cell infiltration (SMD = 1.45). In accordance with standard GRADE guidance, the certainty ratings for outcomes with large and very large effect sizes were adjusted to reflect the increased confidence warranted by effects of this magnitude. A condensed summary of the pooled standardized mean differences, sample sizes, and final GRADE certainty ratings for all primary outcomes is presented in Table 8. This summary table was constructed in Microsoft Word and draws directly from the complete GRADE evidence profile, which is provided in its entirety as Supplementary Table S5. The complete profile includes detailed explanations for each certainty rating, including the specific reasons for downgrading and upgrading, as well as the legend for the GRADE certainty symbols. The evidence presented therefore provides a transparent account of the methodological limitations present in the literature, while documenting the robust and consistent nature of the tumour microenvironment remodeling induced by immune checkpoint blockade.

### 4.6. Limitations

Several limitations warrant careful consideration. First, the preclinical synthesis was deliberately restricted to the unmodified B16-F10 melanoma model in immunocompetent C57BL/6 mice. While this stringent standardization enhanced internal validity and reduced experimental variance, it necessarily limits the generalizability of the findings to other syngeneic models (e.g., YUMM, RET) and to the broader landscape of melanoma heterogeneity. Consequently, the preclinical findings should be interpreted as specific to this model system and extrapolated to other contexts with due circumspection. Second, the completeness of reporting in the primary preclinical studies was suboptimal, as reflected in the SYRCLE and ARRIVE 2.0 assessments. The predominance of “Unclear” and “Not reported” judgments across domains such as randomisation, blinding, and sample size justification introduces uncertainty regarding the internal validity of individual studies, although sensitivity analyses excluding high-ROB studies confirmed the robustness of the pooled estimates. Third, the clinical meta-analysis was limited by the small number of studies reporting certain outcomes (e.g., Ki-67, apoptosis), by heterogeneity in prior treatments and lines of therapy across the included cohorts, and by the reliance on varied and often non-standardized measurement methods, which necessitated the use of standardized mean differences and precluded direct comparison of absolute effect magnitudes with the preclinical SMDs. Fourth, given the fact that the majority of primary studies presented results only graphically, a substantial proportion of data points were extracted from published graphs using the validated tool WebPlotDigitizer; this approach is widely accepted in meta-analytic research and allowed inclusion of otherwise unavailable data, although it may have introduced minor imprecision in individual effect estimates. Fifth, publication bias, while formally not significant for the majority of outcomes, could not be definitively excluded for IFN-γ (preclinical, Egger’s *p* = 0.062) for CD8 in the clinical arm (Egger’s *p* = 0.03), where mild funnel plot asymmetry was observed.

### 4.7. Future Directions and Translational Relevance

This systematic review and meta-analysis illuminates several priority areas for future investigation. In the preclinical domain, there is a pressing need for studies that systematically characterize **spatial and stromal remodeling**, including quantitative assessments of fibroblast activation, extracellular matrix deposition, and vascular normalization. The incorporation of advanced multiplex imaging and spatial transcriptomics would greatly enhance the resolution of TME characterization. Furthermore, the striking divergence in PD-L1 dynamics between murine and human tumours warrants dedicated mechanistic studies to elucidate the cellular and molecular determinants of adaptive immune resistance in the B16F10 model. In the clinical arena, the findings underscore the imperative for **harmonized TME biomarker reporting**, including **standardized protocols** for tissue collection, processing, and analytical pipelines. The integration of **circulating biomarkers**, such as serum CXCL10 and IFN-γ, which were consistently elevated in preclinical models [25,43,62], with intratumoral assessments may offer a minimally invasive window into TME dynamics and warrants prospective validation. Finally, the superior efficacy of anti-PD-L1-containing regimens in the preclinical meta-regression provides a compelling rationale for prioritizing PD-L1-targeted combinations in future preclinical and early-phase clinical trials, particularly in tumours characterized by high baseline immune infiltration and an inflamed TME. Equally pressing is the need for the field to adopt a **unified system of measurement units** for key TME parameters. The present literature is fragmented by a bewildering array of scales; units that are each scientifically valid but collectively create a chaotic landscape that complicates cross-study synthesis and obscures true biological signals. The development and community-wide endorsement of **standardized reporting conventions**, building upon existing frameworks such as the ARRIVE 2.0 guidelines for preclinical studies and the SYRCLE risk-of-bias tool, would greatly enhance the reproducibility and meta-analytic potential of both preclinical and clinical immuno-oncology research. Prospective studies that link harmonized TME read-outs to longitudinal clinical outcomes and multi-omics data will ultimately be required to translate these research priorities into routine practice.

## 5. Conclusions

In conclusion, this dual systematic review and meta-analysis offers a comprehensive, quantitative cartography of tumour microenvironment reprogramming by immune checkpoint blockade, spanning preclinical models and patients with melanoma. The evidence converges on a **conserved axis of immune activation:** checkpoint inhibition unleashes a cascade defined by heightened CD8^+^ T-cell infiltration, amplified IFN-γ signaling, and a decisive shift in the effector to suppressor equilibrium, a constellation of changes that collectively suppress tumour cell proliferation and drive robust apoptotic death. The B16F10 preclinical evidence base, enriched by experimental control and granular terminal effector data, serves as a **powerful complement to the clinical evidence**, illuminating mechanistic subtleties that remain largely obscured in human studies. Yet the discordant behavior of PD-L1 expression and the striking potency of anti-PD-L1-based regimens simultaneously affirm the translational value and expose the inherent constraints of the B16-F10 model, compelling a nuanced, context-sensitive extrapolation of preclinical insight. The superior effector amplification by anti-PD-L1-containing regimens was observed exclusively in the preclinical B16F10 model and has not been evaluated in the clinical quantitative arm; its translational applicability therefore awaits prospective validation. Taken together, this synthesis establishes **a foundational resource, both a benchmark and a compass**, to steer the rational design of next-generation immunotherapeutic strategies and guide the development of harmonized, robust biomarkers for patient stratification and longitudinal response monitoring.

## Figures and Tables

**Table 1:** Meta-analytic estimates for the primary outcomes (main analysis, without predictors); Preclinical Arm *k* = number of data points; CI = confidence interval; SMD = standardized mean difference (Hedges’ *g*). CD8: CD8^+^ T-cell infiltration; CD8/Treg: CD8/regulatory T-cell ratio; PD-L1: programmed death-ligand 1; IFN-γ: interferon-γ; Ki-67: Ki-67 proliferation index; Apoptosis: apoptotic cell death.

Outcome	*k*	SMD [95% CI]	*p*	I^2^ (%)	τ^2^	Q (df)	*p*(Q)
**CD8**	41	1.451 [1.051, 1.852]	<0.001	68.4	1.025	126.71 (40)	<0.001
**CD8/Treg**	17	0.913 [0.281, 1.545]	0.005	73.7	1.132	60.77 (16)	<0.001
**PD-L1**	4	−0.882 [−1.483, −0.282]	0.004	0.0	0.000	0.68 (3)	0.878
**IFN-γ**	21	1.784 [0.947, 2.621]	<0.001	81.3	2.640	106.66 (20)	<0.001
**Ki67**	9	−1.426 [−2.702, −0.150]	0.028	87.5	2.913	63.85 (8)	<0.001
**Apoptosis**	7	3.537 [2.129, 4.946]	<0.001	80.2	2.479	30.34 (6)	<0.001

**Table 2:** Meta-analytic estimates for the primary outcomes (main analysis, without predictors); Clinical Arm *k* = number of data points; CI = confidence interval; SMD = standardized mean difference (Hedges’ *g*).

Outcome	*k*	SMD [95% CI]	*p*	I^2^ (%)	τ^2^	Q (df)	*p*(Q)
**CD8**	19	0.723 [0.442, 1.005]	<0.001	61.5	0.175	46.72 (18)	<0.001
**CD8/Treg**	8	−0.211 [−1.116, 0.695]	0.649	83.9	1.362	43.38 (7)	<0.001
**PD-L1**	10	0.670 [0.257, 1.084]	0.001	74.2	0.255	34.83 (9)	<0.001
**IFN-γ**	4	0.585 [−0.027, 1.198]	0.061	60.2	0.220	7.54 (3)	0.057
**Ki-67**	3	0.262 [−0.798, 1.322]	0.628	66.0	0.568	5.88 (2)	0.053
**Apoptosis**	1	–	–	–	–	–	–

**Table 3:** **Meta-regression tests for moderation by drug class for the preclinical arm**; the reference category for all comparisons is anti-CTLA-4 monotherapy; df = degrees of freedom.

Outcome	Q_m_	df	*p*	Significant Moderation?
**CD8**	2.86	3	0.414	No
**CD8/Treg**	9.61	2	0.008	Yes
**PD-L1**	0.66	2	0.718	No
**IFN-γ**	7.08	2	0.029	Yes
**Ki67**	6.65	4	0.156	No
**Apoptosis**	8.33	2	0.016	Yes

**Table 4:** **Meta-regression tests for moderation by drug class for the clinical arm**; the reference category for all comparisons is anti-CTLA-4 monotherapy.

Outcome	Q_m_	df	*p*	Significant Moderation?
**CD8**	2.58	2	0.275	No
**CD8/Treg**	0.02	2	0.990	No
**PD-L1**	1.62	1	0.203	No
**IFN-γ**	1.21	2	0.546	No
**Ki-67**	0.65	1	0.419	No

**Table 5:** **Meta-regression tests for moderation by comparator**; for CD8/Treg, the Q_m_ value was reported as 0.004 in the output, which rounds to 0.00; the *p* value remained 0.949, indicating no moderation.

Outcome	Q_m_	df	*p*	Significant Moderation?
**CD8**	3.94	1	0.047	Yes
**CD8/Treg**	0.00	1	0.949	No
**PD-L1**	10.22	2	0.006	Yes
**IFN-γ**	0.31	1	0.577	No
**Ki-67**	0.28	1	0.598	No

**Table 6:** **Sensitivity analyses for the preclinical arm:** pooled standardized mean differences (SMDs) and 95% confidence intervals. All pooled estimates are statistically significant (*p* < 0.05); heterogeneity statistics were derived from the JASP meta-analytic engine.

Analysis	CD8	CD8/Treg	PD-L1	IFN-γ	Ki67	Apoptosis
**Main (all studies)**	1.451 (1.051–1.852)	0.913 (0.281–1.545)	−0.882 (−1.483 to −0.282)	1.784 (0.947–2.621)	−1.426 (−2.702 to −0.150)	3.537 (2.129–4.946)
**Exclude low-n**	1.326 (0.903–1.749)	0.778 (0.121–1.435)	−0.882 (−1.483 to −0.282)	1.772 (0.864–2.680)	−1.426 (−2.702 to −0.150)	3.537 (2.129–4.946)
**Exclude high-ROB**	1.560 (1.149–1.970)	0.913 (0.281–1.545)	−0.882 (−1.483 to −0.282)	1.784 (0.947–2.621)	−1.598 (−3.154 to −0.041)	4.154 (2.438–5.870)
**Combined**	1.444 (1.007–1.881)	0.778 (0.121–1.435)	−0.882 (−1.483 to −0.282)	1.772 (0.864–2.680)	−1.598 (−3.154 to −0.041)	4.154 (2.438–5.870)

**Table 7:** **Sensitivity analyses for the clinical arm:** pooled SMDs (95% CI) for key parameters. All *p*-values correspond to the pooled effect estimates; heterogeneity statistics were derived from the JASP meta-analytic engine.

Analysis	CD8	CD8/Treg	PD-L1	IFN-γ	Ki-67
**Main (all studies)**	0.723 (0.442–1.005)	−0.211 (−1.116–0.695)	0.670 (0.257–1.084)	0.585 (−0.027–1.198)	0.262 (−0.798–1.322)
**Exclude small-n**	0.716 (0.420–1.011)	−0.529 (−1.603–0.545)	0.628 (0.209–1.048)	0.585 (−0.027–1.198)	0.262 (−0.798–1.322)
**Exclude high-ROB**	0.631 (0.322–0.940)	−0.167 (−0.937–0.604)	0.620 (0.142–1.097)	0.585 (−0.027–1.198)	0.262 (−0.798–1.322)
**Combined**	0.612 (0.298–0.927)	−0.444 (−1.164–0.276)	0.559 (0.073–1.045)	0.585 (−0.027–1.198)	0.262 (−0.798–1.322)

**Table 8:** **Summary of Pooled Effects, Sample Sizes, and GRADE Certainty of Evidence for TME Outcomes;** *k* = number of data points; *N* = total number of animals (preclinical) or patients (clinical) in the respective group. **GRADE Certainty:** ⊕⊕⊕◯ = Moderate; ⊕⊕◯◯ = Low; ⊕◯◯◯ = Very Low.

Outcome	*k*	SMD [95% CI]	I^2^ (%)	*N* (Interv)	*N* (Contr)	GRADE Certainty
**Preclinical (B16F10)**						
**CD8^+^ T-cell infiltration**	41	**1.45** [1.05, 1.85]	68.4	270	259	⊕⊕⊕◯
**CD8/Treg ratio**	17	**0.91** [0.28, 1.55]	73.7	85	85	⊕⊕◯◯
**PD-L1 expression**	4	**−0.88** [−1.48, −0.28]	0	30	30	⊕◯◯◯
**IFN-γ production**	21	**1.78** [0.95, 2.62]	81.3	107	107	⊕⊕⊕◯
**Ki-67 proliferation**	9	**−1.43** [−2.70, −0.15]	87.5	63	67	⊕⊕◯◯
**Apoptosis**	7	**3.54** [2.13, 4.95]	80.2	56	60	⊕⊕⊕◯
**Clinical**						
**CD8^+^ T-cell infiltration**	19	**0.72** [0.44, 1.01]	61.5	1205	449	⊕⊕◯◯
**CD8/Treg ratio**	8	**−0.21** [−1.12, 0.70]	83.9	116	77	⊕⊕◯◯
**PD-L1 expression**	10	**0.67** [0.26, 1.08]	74.2	1078	324	⊕⊕◯◯
**IFN-γ production**	4	**0.59** [−0.03, 1.20]	60.2	780	193	⊕⊕◯◯
**Ki-67 proliferation**	3	**0.26** [−0.80, 1.32]	66.0	30	35	⊕◯◯◯

## Data Availability

The curated dataset supporting this meta-analysis is contained within the article and its Supplementary Materials. Further inquiries can be directed to the corresponding author. The study protocol is registered at PROSPERO (CRD420261374242) and available at https://www.crd.york.ac.uk/PROSPERO/view/CRD420261374242, accessed on 24 May 2026.

## Supplementary Materials

The following supporting information can be downloaded at: https://www.mdpi.com/article/10.3390/cells15131182/s1, Supplementary File S1, PROSPERO international prospective register of systematic reviews (CRD420261374242); Supplementary File S2, PRISMA 2020 checklist. Supplementary Tables: Table S1: Full database search strategies for the systematic identification of preclinical and clinical studies; Table S2: Curated meta-analytic dataset: individual study means, standard deviations, sample sizes, drug-class assignments, and comparators used for the quantitative synthesis (preclinical and clinical arms); Table S3: Bubble plots for the meta-regression of drug class on each TME outcome; Table S4: Peripheral-only biomarker studies with no intratumoral TME data; Table S5: Full GRADE evidence profile for all primary outcomes.

## Abbreviations

The following abbreviations are used in this manuscript:

ADCC	Antibody-Dependent Cellular Cytotoxicity
ADCP	Antibody-Dependent Cellular Phagocytosis
Aire	Autoimmune Regulator
AJCC	American Joint Committee on Cancer
APC	Antigen-Presenting Cell
ARRIVE	Animal Research: Reporting of In Vivo Experiments
BRAF	v-Raf Murine Sarcoma Viral Oncogene Homolog B
CCL	C-C Motif Chemokine Ligand
CD	Cluster of Differentiation
CDKN2A	Cyclin-Dependent Kinase Inhibitor 2A
CI	Confidence Interval
CM	Cutaneous Melanoma
CTLA-4	Cytotoxic T-Lymphocyte-Associated Protein 4
ctDNA	Circulating Tumour DNA
CXCL	C-X-C Motif Chemokine Ligand
CXCR	C-X-C Motif Chemokine Receptor
DFS	Disease-Free Survival
DMFS	Distant Metastasis-Free Survival
DSP	Digital Spatial Profiling
ELISA	Enzyme-Linked Immunosorbent Assay
FcγR	Fc Gamma Receptor
FDA	U.S. Food and Drug Administration
FOXP3	Forkhead Box P3
Gal-3	Galectin-3
GZMA	Granzyme A
GZMB	Granzyme B
H&E	Hematoxylin and Eosin
HLA	Human Leukocyte Antigen
HPF	High-Power Field
HR	Hazard Ratio
iCAF	Inflammatory Cancer-Associated Fibroblast
ICI	Immune Checkpoint Inhibitor
IDO	Indoleamine 2,3-Dioxygenase
IFN-γ	Interferon Gamma
Ig	Immunoglobulin
IHC	Immunohistochemistry
IL	Interleukin
iNOS	Inducible Nitric Oxide Synthase
irAE	Immune-Related Adverse Event
IRS	Immunoreactive Score
I^2^	I-Squared (Heterogeneity Statistic)
JASP	Jeffreys’s Amazing Statistics Program
LAG-3	Lymphocyte Activation Gene 3
LDH	Lactate Dehydrogenase
MAIT	Mucosal-Associated Invariant T Cell
MAPK	Mitogen-Activated Protein Kinase
MCP-4	Monocyte Chemotactic Protein4
MDSC	Myeloid-Derived Suppressor Cell
MEK	Mitogen-Activated Extracellular Signal-Regulated Kinase
MELscore	Melanoma PD-L1 Expression Score
MHC	Major Histocompatibility Complex
MITF	Microphthalmia-Associated Transcription Factor
MPR	Major Pathological Response
mRNA	Messenger Ribonucleic Acid
NF1	Neurofibromin 1
NK	Natural Killer
NKT	Natural Killer T-Cell
NRAS	Neuroblastoma RAS Viral Oncogene Homolog
OPG	Osteoprotegerin
ORR	Objective Response Rate
OS	Overall Survival
OXPHOS	Oxidative Phosphorylation
PD-1	Programmed Cell Death Protein 1
PD-L1	Programmed Cell Death Ligand 1
PD-L2	Programmed Cell Death Ligand 2
PFS	Progression-Free Survival
PI3K	Phosphoinositide 3-Kinase
PP2A	Protein Phosphatase 2A
PRAME	PReferentially Expressed Antigen in Melanoma
PRF1	Perforin1
PRISMA	Preferred Reporting Items for Systematic Reviews and Meta-Analyses
pSTAT1	Phosphorylated Signal Transducer and Activator of Transcription-1
PTEN	Phosphatase and Tensin Homolog
Q_e_	Cochran’s Q (Heterogeneity Test)
Q_m_	Meta-regression Moderation Test Statistic
RFS	Recurrence-Free Survival
RNA-seq	RNA Sequencing
ROB	Risk of Bias
ROBINS-I	Risk of Bias in Non-Randomised Studies of Interventions
ROC AUC	Receiver Operating Characteristic Area Under The Curve
SHP-2	Src Homology 2 Domain-Containing Phosphatase 2
SMD	Standardized Mean Difference
sPD-1	Soluble Programmed Cell Death Protein 1
SR/MA	Systematic Review and Meta-Analysis
SYRCLE	Systematic Review Centre for Laboratory Animal Experimentation
T-VEC	Talimogene Laherparepvec
TAM	Tumour-Associated Macrophage
TCGA	The Cancer Genome Atlas
TCR	T-cell Receptor
TEM	T Effector Memory
TIGIT	T-cell Immunoreceptor With Ig And ITIM Domains
TIL	Tumour-Infiltrating Lymphocyte
TIM-3	T-cell Immunoglobulin and Mucin Domain-Containing Protein 3
TLS	Tertiary Lymphoid Structure
TMB	Tumour Mutational Burden
TME	Tumour Microenvironment
TNF-α	Tumour Necrosis Factor Alpha
TNFRSF9	Tumour Necrosis Factor Receptor Superfamily Member9
TP53	Tumour Protein p53
Treg	Regulatory T Cell
TUNEL	Terminal Deoxynucleotidyl Transferase dUTP Nick End Labeling
UV	Ultraviolet
VEGF	Vascular Endothelial Growth Factor
WHO	World Health Organization
ZAP-70	Zeta-Chain-Associated Protein Kinase 70
τ^2^	Tau Squared (Between-Study Variance)
