# Supplementary material for: Reshaping the Battlefield: Reprogramming the Melanoma Tumour Microenvironment (TME) by Anti-CTLA-4, Anti-PD-1, and Anti-PD-L1 Monotherapy and Combination Therapy: A Systematic Review and Meta-Analysis of Preclinical and Clinical Evidence
Source: Cells. 2026 Jun 29;15(13):1182. doi: 10.3390/cells15131182 (PMC13359709; doi:10.3390/cells15131182)
Supplement: Supplementary file 1 [file cells-15-01182-s001.zip › 1. KARAKOUSIS- SUPPLEMENTARY FILE S1- PROSPERO_CRD420261374242.pdf]

Reprogramming the Melanoma Tumor Microenvironment (TME) by anti-CTLA-4, anti-PD-1, and anti-PD-L1 Monotherapy and Combination Therapy: a Systematic Review and Meta-Analysis of Preclinical and Clinical Evidence

VASILEIOS ALEXANDROS KARAKOUSIS, STYLIANOS MANTALOVAS

Citation
VASILEIOS ALEXANDROS KARAKOUSIS, STYLIANOS MANTALOVAS. Reprogramming the Melanoma Tumor Microenvironment (TME) by anti-CTLA-4, anti-PD-1, and anti-PD-L1 Monotherapy and Combination Therapy: a Systematic Review and Meta-Analysis of Preclinical and Clinical Evidence. PROSPERO 2026 CRD420261374242. Available from https://www.crd.york.ac.uk/PROSPERO/view/CRD420261374242.

REVIEW TITLE AND BASIC DETAILS

Review title
Reprogramming the Melanoma Tumor Microenvironment (TME) by anti-CTLA-4, anti-PD-1, and anti-PD-L1 Monotherapy and Combination Therapy: a Systematic Review and Meta-Analysis of Preclinical and Clinical Evidence

Condition or domain being studied
Skin melanoma; Immunotherapy; Immune checkpoint inhibitors

Cutaneous melanoma is an aggressive malignancy with high metastatic potential, and immune checkpoint inhibitors have transformed its treatment by reprogramming the tumor microenvironment (TME).

Rationale for the review
While immune checkpoint blockade has transformed melanoma therapy, clinical outcomes remain profoundly heterogeneous. The tumor microenvironment (TME) constitutes the pivotal biological interface where therapeutic efficacy, resistance, and toxicity are ultimately determined. This systematic review and meta-analysis will quantitatively and qualitatively synthesize the reprogramming of the melanoma TME induced by anti-CTLA-4, anti-PD-1, and anti-PD-L1, administered as monotherapy or in combination. A direct comparison of the effects elicited by these distinct therapeutic regimens will be performed across spatial architecture, immune cell composition, molecular expression, and soluble mediator profiles. Critically, this review will deliberately integrate evidence from stringent preclinical B16F10/C57BL/6 murine models and human clinical studies to build a translational understanding that directly informs therapeutic development. By specifically targeting the TME as the primary outcome and correlating TME phenotypes with clinical outcomes, this work aims to decipher distinct and synergistic immunologic signatures, identify predictive biomarkers of response and resistance, and elucidate TME features associated with immune-related adverse events. To our knowledge, this is the first dual SR/MA to comprehensively map the multifaceted remodelling of the melanoma TME across all three foundational immune checkpoint inhibitors' classes across species. Ultimately, this review will construct a quantitative evidential scaffold to guide the rational design of next-generation combination strategies with improved efficacy-toxicity profiles.

Review objectives
This SR/MA comprehensively synthesises and compares melanoma tumor microenvironment (TME) reprogramming induced by anti-CTLA-4, anti-PD-1, and anti-PD-L1 monotherapy and combination therapy across preclinical and clinical settings. The specific objectives are: (1) provide a comparative synthesis of spatial architecture and stromal composition, including fibrosis and angiogenesis; (2) conduct a

comparative analysis of density and distribution of tumor-infiltrating immune cells ($CD8^+$ T cells, Tregs, MDSCs, TAMs) and tumor cell density; (3) perform a comparative evaluation of expression of immune checkpoint molecules and tumor-associated antigens (CTLA-4, PD-1, PD-L1, LAG-3, TIM-3, TIGIT, VISTA, PRAME); (4) undertake a comparative assessment of local and systemic soluble mediator profiles, including cytokines, chemokines, and biomarkers (IFN-γ, TNF-α, IL-1β, IL-2, IL-4, IL-6, IL-10, IL-12, IL-17A, TGF-β, CCL2/3/4/5/22, CXCL9/10, LDH); (5) correlate TME changes with clinical and preclinical efficacy outcomes; (6) appraise TME alterations as predictive or prognostic biomarkers; (7) pinpoint TME signatures linked to primary or acquired resistance; (8) determine TME hallmarks tied to immune-related adverse events; (9) evaluate preclinical-clinical concordance and translational gaps; and (10) extract mechanistic insights to guide next-generation immunotherapy development.

Keywords

Melanoma; Tumor microenvironment; CTLA-4; PD-1; PD-L1; Ipilimumab; Nivolumab; Atezolizumab; B16F10; C57BL/6; Biomarkers; Translational research

Country

Greece

ELIGIBILITY CRITERIA

Population

Included

Inclusion: Clinical: Human patients (adults ≥18 years) with a confirmed diagnosis of unresectable or metastatic cutaneous melanoma (Stage III/IV). Eligible study designs include randomised controlled trials (RCTs), non randomised interventional studies, and prospective cohort studies. Preclinical: In vivo studies using immunocompetent C57BL/6 mice implanted subcutaneously with unmodified, parental B16F10 cutaneous melanoma cells and receiving intraperitoneal administration of the intervention. All included studies must have a controlled experimental design (i.e., treatment group vs. control group). Only intratumoral TME data extracted directly from the tumor mass are considered.

Excluded

Exclusion: (1) Non relevant articles (e.g., other cancer types, non melanoma skin cancers). (2) Studies not involving treatment with anti-CTLA-4, anti-PD-1, or anti-PD-L1 agents. (3) Studies evaluating mucosal, uveal, or acral lentiginous melanoma, unless separate extractable cutaneous melanoma data are available. (4) Clinical or preclinical studies lacking extractable intratumoral TME data (peripheral blood, serum, or splenic only studies are excluded). (5) Animal studies not conducted in vivo (e.g., in vitro or ex vivo only). (6) Preclinical studies using mouse strains other than C57BL/6, or melanoma cell lines other than unmodified B16F10, or employing tumor inoculation routes other than subcutaneous injection, or utilizing drug administration routes other than intraperitoneal delivery. (7) Case reports, case series with fewer than 5 subjects (clinical) or animals (preclinical), or systematic reviews based on case reports. (8) Letters to the editor, commentaries, or conference abstracts lacking full text peer reviewed articles. (9) Irrelevant systematic reviews or meta analyses. (10) Studies with only abstracts available or full text inaccessible after exhaustive search. (11) Studies lacking quantitative data on at least one predefined Tumor Microenvironment (TME) outcome (e.g., immune cell density, spatial metrics, cytokine levels). (12) Retrospective observational studies, including retrospective cohort and case control studies.

Intervention(s) or exposure(s)

Included

Ipilimumab; Tremelimumab; Nivolumab; Pembrolizumab; Atezolizumab; Avelumab; Durvalumab

Administration of anti-CTLA-4 (e.g., ipilimumab, tremelimumab), anti-PD-1 (e.g., nivolumab, pembrolizumab), or anti-PD-L1 (e.g., atezolizumab, avelumab, durvalumab) as monotherapy, or any combination (e.g., anti-CTLA-4 + anti-PD-1, anti-CTLA-4 + anti-PD-L1, anti-PD-1 + anti-PD-L1), at any dose or schedule.

Excluded

Studies that do not involve treatment with anti-CTLA-4 (e.g., ipilimumab, tremelimumab), anti-PD-1 (e.g., nivolumab, pembrolizumab), or anti-PD-L1 agents (e.g., atezolizumab, avelumab, durvalumab) in at least one dedicated arm of their analysis (e.g., mixed studies lacking an eligible arm are excluded).

Comparator(s) or control(s)

Included
PICO tags selected: Placebo; Active control; Vehicle Control; Usual Care; Evaluating Response To Treatment; Target Lesion Recurrence

Passive comparator: placebo or untreated control. Active comparator: monotherapy versus combination therapy or different immunotherapies. Standard of care: chemotherapy or targeted therapy. For clinical studies, three specific comparator categories were analysed: post-treatment versus pre-treatment, responders versus non-responders, and no-relapse versus relapse, with the favourable outcome (post-treatment, responder, no-relapse) always compared against the unfavourable counterpart.

Excluded
No specific comparator exclusion criteria; studies meeting the overall eligibility criteria with any of the predefined comparators are included.

Study design

Both randomized and nonrandomized study types will be included.

Included
Clinical: Randomized controlled trials (RCTs), non randomized interventional studies, and prospective cohort studies with a defined immunotherapy intervention. Preclinical: Controlled in vivo experimental studies.

Excluded
In vitro and ex vivo experiments, case reports and case series with fewer than five subjects (clinical) or animals (preclinical), letters to the editor, commentaries, and conference abstracts for which full peer reviewed articles are not available, irrelevant systematic reviews and meta analyses, studies with only abstracts accessible or for which the full text could not be obtained after exhaustive search, and retrospective observational studies, including retrospective cohort and case control designs.

Context

This dual systematic review integrates evidence from two distinct settings: (1) Clinical studies conducted in hospital or cancer center settings involving adult patients with unresectable or metastatic cutaneous melanoma; (2) Preclinical in vivo studies performed in laboratory animal facilities using immunocompetent C57BL/6 mice implanted subcutaneously with unmodified B16F10 tumours and receiving intraperitoneal immunotherapy. No geographic restrictions are applied to either clinical or preclinical studies. Only studies with extractable intratumoral TME data are eligible; exclusively peripheral blood or serum biomarker studies are excluded from both settings.

TIMELINE OF THE REVIEW

Date of first submission to PROSPERO

23 April 2026

Review timeline

Start date: 20 January 2026. End date: 20 June 2026.

Date of registration in PROSPERO

23 April 2026

AVAILABILITY OF FULL PROTOCOL

Availability of full protocol

A full protocol has been written and uploaded to PROSPERO. The protocol may be accessed through this link https://www.crd.york.ac.uk/PROSPEROFILES/8c46af9d6c83f22ebf421d25cab65e1b.pdf.

SEARCHING AND SCREENING

Search for unpublished studies

Only published studies will be sought.

Main sources that will be searched

The main databases to be searched are *PubMed* and *Scopus*.

Other important or specialist databases that will be searched
Web of Science, Cochrane Library

Search language restrictions

The review will only include studies published in English.

Search date restrictions

There are no search date restrictions.

Other methods of identifying studies

Other studies will be identified by: *reference list checking (backward citation searching)*.

Link to search strategy

A full search strategy has been uploaded to PROSPERO. The PDF may be accessed through this link https://www.crd.york.ac.uk/PROSPEROFILES/ab71f1695d15b7927a79979afa71167b.pdf.

Selection process

Studies will be screened independently by at least two people (or person/machine combination) with a process to resolve differences.

Other relevant information about searching and screening

None

DATA COLLECTION PROCESS

Data extraction from published articles and reports

Data will be extracted independently by at least two people (or person/machine combination) with a process to resolve differences.

Authors will be asked to provide any required data not available in published reports.

Study risk of bias or quality assessment

Risk of bias will be assessed using: *Cochrane RoB-2* and *ROBINS-I*

SYRCLE's Risk of Bias tool (for preclinical animal studies)

Data will be assessed independently by at least two people (or person/machine combination) with a process to resolve differences.

Additional information will be sought from study investigators if required information is unclear or unavailable in the study publications/reports.

Reporting bias assessment

To assess potential publication bias and selective outcome reporting, funnel plots and Egger's statistical test will be used when a sufficient number of studies (≥10) are included in a meta-analysis.

Certainty assessment

The overall certainty (or quality) of the evidence for key outcomes will be assessed. For clinical evidence, the Grading of Recommendations, Assessment, Development and Evaluations (GRADE) framework will be applied. For preclinical evidence, a modified GRADE approach adapted for animal studies (as developed by SYRCLE) will be used. Both systems classify evidence into four levels: High, Moderate, Low, or Very Low.

OUTCOMES TO BE ANALYSED

Main outcomes

1. Modifications in tumor microenvironment spatial architecture and ultrastructure (e.g., fibrosis, angiogenesis, stromal organisation). 2. Variations in the density and distribution of tumor-infiltrating immune cells (e.g., CD8+ T cells, Tregs, MDSCs, TAMs) and tumor cells. 3.

Changes in the expression of immune-regulatory and tumor-associated molecules (e.g., CTLA-4, PD-1, PD-L1, PRAME). 4. Alterations in the levels of local and systemic soluble mediators (e.g., cytokines, chemokines, IFN-γ, LDH). 5. Changes in tumor cell proliferation (e.g., Ki-67 index). 6. Induction of tumor cell apoptosis (e.g., TUNEL, cleaved caspase-3). For all outcomes, measurements will be taken at any time point following immunotherapy initiation, using techniques including IHC, flow cytometry, ELISA, or transcriptomics. The effect measure for synthesis will be the standardised mean difference (Hedges' g) with 95% confidence intervals.

Additional outcomes

1. Efficacy outcomes: Tumor response (e.g., ORR), survival (e.g., PFS, OS), and tumor growth metrics. 2. Quantitative spatial metrics describing cellular architecture within the TME (e.g., proximity analyses). For all additional outcomes, measurements will be taken at any time point following immunotherapy initiation, using techniques including clinical imaging, caliper measurement, or multiplex imaging. The effect measure for synthesis will be the standardised mean difference (Hedges' g) or risk ratio, as appropriate, with 95% confidence intervals.

PLANNED DATA SYNTHESIS

Strategy for data synthesis

A convergent synthesis approach will be employed, integrating quantitative **meta-analysis** where possible with comprehensive **narrative synthesis.** All methods adhered to the Preferred Reporting Items for Systematic Reviews and Meta-Analyses **(PRISMA) 2020** statement.

For the quantitative meta-analysis, analyses will be conducted using **JASP software (Version 0.96.0.0),** which provides pooled estimates and generates forest plots. For continuous outcomes such as immune cell density or cytokine levels, the mean difference or standardised mean difference with 95% confidence intervals will be calculated from reported data. For dichotomous efficacy outcomes such as response rates, risk ratios with 95% confidence intervals will be calculated. A random-effects model using the inverse variance method will be employed for all meta-analyses to account for anticipated clinical and methodological heterogeneity. Statistical heterogeneity among studies will be assessed using **Cochran's Q statistic** with a significance level of p less than 0.10 and quantified using the **I squared statistic,** where an I squared value greater than 50% will be considered indicative of substantial heterogeneity. The between-study variance will be estimated using **τ^2 (tau-squared),** with its **square root τ (tau)** representing the standard deviation of the true effect sizes across studies. Publication bias will be assessed using **funnel plots** and **Egger's regression test** when ten or more studies are included in a meta analysis.

For outcomes where quantitative pooling is not feasible due to heterogeneity in measurement or reporting, a structured narrative synthesis will be performed. Findings will be summarised and compared across studies, organised by outcome domain.

Pre-planned **subgroup analyses** will investigate sources of heterogeneity. For both arms, subgroups will be defined by treatment regimen: (i) anti-CTLA-4 monotherapy, (ii) anti-PD-1 monotherapy, (iii) anti-PD-L1 monotherapy, (iv) anti-CTLA-4 + anti-PD-1 combination, (v) anti-CTLA-4 + anti-PD-L1 combination, and (vi) other dual/triple combinations of the three drug classes. For the clinical arm, subgroups will also include comparator type: (i) post-treatment versus pre-treatment, (ii) responders versus non-responders, and (iii) no recurrence versus recurrence. Preclinical and clinical data will be synthesised separately and will not be pooled across species or study design.

Sensitivity analyses will be performed to examine the robustness of the primary meta-analytic findings. These will include: (i) exclusion of studies judged to be at high risk of bias according to the design specific tools (SYRCLE, RoB 2, or ROBINS I), and (ii) exclusion of studies with very small sample sizes, defined as fewer than five subjects per group (clinical) or fewer than five animals per group (preclinical). A combined sensitivity analysis excluding both sets of studies will also be conducted for each arm.

CURRENT REVIEW STAGE

Stage of the review at this submission 1 change

Review stage	Started	Completed
Pilot work	✓	✓
Formal searching/study identification	✓	✓
Screening search results against inclusion criteria	✓	✓
Data extraction or receipt of IPD	✓	✓
Risk of bias/quality assessment	✓	✓
Data synthesis	✓	✓

Review status
The review is completed.

Publication of review results
Results of the review will be published in English and Greek.

REVIEW AFFILIATION, FUNDING AND PEER REVIEW

Review team members
Dr VASILEIOS ALEXANDROS KARAKOUSIS (review guarantor and contact) ORCID: 0009-0004-3719-4715. Aristotle University of Thessaloniki. Greece.

No conflict of interest declared.

Dr STYLIANOS MANTALOVAS. Aristotle University of Thessaloniki. Greece.

No conflict of interest declared.

Named contact
Dr VASILEIOS ALEXANDROS KARAKOUSIS (vkarakob@auth.gr). ORCID: 0009-0004-3719-4715. Aristotle University of Thessaloniki. Greece.

Review affiliation
Aristotle University of Thessaloniki, Faculty of Health Sciences, School of Medicine, 54124, Thessaloniki, Greece

Funding source
Review has no funding and no agreed support from an academic institution and is done in authors' own time.

Peer review
There has been no peer review of this planned review.

ADDITIONAL INFORMATION

Additional information
Melanoma is an aggressive cutaneous malignancy with a high metastatic potential. Immune checkpoint inhibitors, anti-CTLA-4, anti-PD-1 and anti-PD-L1, either as monotherapies or in combination, have transformed therapeutic outcomes. The tumor microenvironment (TME) is a critical determinant of treatment efficacy, resistance, and toxicity. This systematic review and meta-analysis will synthesize and compare preclinical and clinical evidence on TME reprogramming induced by these foundational immunotherapies.

Review conflict of interest
Declared individual interests are recorded under team member details.. No additional interests are recorded for this review.

Medical Subject Headings
Melanoma; Tumor Microenvironment; CTLA-4 Antigen; Programmed Cell Death 1 Receptor; B7-H1 Antigen; Immune Checkpoint Inhibitors; Ipilimumab; Nivolumab; Biomarkers; Translational Medical Research

Revision note 1 change

Stage boxes were updated to reflect completion of data extraction, risk of bias assessment, and data synthesis.

SIMILAR REVIEWS

Check for similar records already in PROSPERO

PROSPERO identified a number of existing PROSPERO records that were similar to this one (last check made on 20 April 2026). These are shown below along with the reasons given by that the review team for the reviews being different and/or proceeding.

- Differences of dermatological adverse events associated with anti-PD-1, anti-PD-L1, anti-CTLA-4: a systematic review and meta-analysis [published 5 July 2020] [CRD42020182247]. The review was judged **not to be similar**
- Response to treatment and adverse events for anti-PD-1, anti-PD-L1, and anti-CTLA-4 drugs in malignancies of the hematopoietic and lymphoid tissues: systematic review and meta-analysis [published 28 April 2020] [CRD42020165204]. The review was judged **not to be similar**
- Predictive effect of biomarkers on determining outcome from anti-PD-1/PD-L1 treatment in head and neck squamous cell carcinoma: a systematic review and meta-analysis [published 22 January 2018] [CRD42018086510]. The review was judged **not to be similar**

PROSPERO version history 1 change

- Version 1.1, published 24 May 2026
- Version 1.0, published 23 Apr 2026

Disclaimer

The content of this record displays the information provided by the review team. PROSPERO does not peer review registration records or endorse their content.

PROSPERO accepts and posts the information provided in good faith; responsibility for record content rests with the review team. The guarantor for this record has affirmed that the information provided is truthful and that they understand that deliberate provision of inaccurate information may be construed as scientific misconduct.

PROSPERO does not accept any liability for the content provided in this record or for its use. Readers use the information provided in this record at their own risk.

Any enquiries about the record should be referred to the named review contact
