# Supplementary material for: Reshaping the Battlefield: Reprogramming the Melanoma Tumour Microenvironment (TME) by Anti-CTLA-4, Anti-PD-1, and Anti-PD-L1 Monotherapy and Combination Therapy: A Systematic Review and Meta-Analysis of Preclinical and Clinical Evidence
Source: Cells. 2026 Jun 29;15(13):1182. doi: 10.3390/cells15131182 (PMC13359709; doi:10.3390/cells15131182)
Supplement: Supplementary file 1 [file cells-15-01182-s001.zip › 3. KARAKOUSIS- SUPPLEMENTARY TABLES.pdf]

Supplementary Table S1. Full database search strategies for the systematic identification of preclinical and clinical studies.

A. PUBMED/MEDLINE

Search Type	PubMed/MEDLINE Search String
Clinical Studies	(("Melanoma"[Mesh] OR melanoma*[tiab] OR "cutaneous melanoma"[tiab] OR "malignant melanoma"[tiab] OR "skin melanoma"[tiab] OR "melanoma metastasis"[tiab] OR "metastatic melanoma"[tiab]) AND ("Ipilimumab"[Mesh] OR "Nivolumab"[Mesh] OR "Pembrolizumab"[Mesh] OR "Immune Checkpoint Inhibitors"[Mesh] OR "CTLA-4 Antigen"[Mesh] OR "Programmed Cell Death 1 Receptor"[Mesh] OR ipilimumab[tiab] OR nivolumab[tiab] OR pembrolizumab[tiab] OR atezolizumab[tiab] OR avelumab[tiab] OR durvalumab[tiab] OR "anti-CTLA4"[tiab] OR "anti-CTLA-4"[tiab] OR "anti CTLA4"[tiab] OR "CTLA-4 inhibitor"[tiab] OR "CTLA4 inhibitor"[tiab] OR "anti-PD1"[tiab] OR "anti-PD-1"[tiab] OR "anti PD1"[tiab] OR "PD-1 inhibitor"[tiab] OR "PD1 inhibitor"[tiab] OR "anti-PD-L1"[tiab] OR "PD-L1 inhibitor"[tiab] OR "PDL1 inhibitor"[tiab] OR "immune checkpoint inhibitor"[tiab] OR "checkpoint inhibitor"[tiab] OR "checkpoint blockade"[tiab]) AND (("Tumor Microenvironment"[Mesh] OR "tumor microenvironment"[tiab] OR "tumour microenvironment"[tiab] OR "TME"[tiab] OR "spatial architecture"[tiab] OR "stromal remodeling"[tiab] OR "tumor stroma"[tiab] OR "tumor infiltrating lymphocyte"[tiab] OR "tumor-infiltrating lymphocyte"[tiab] OR "TIL"[tiab] OR "CD8"[tiab] OR "CD8+"[tiab] OR "CD3"[tiab] OR "cytotoxic T cell"[tiab] OR "CD4"[tiab] OR "CD4+"[tiab] OR "helper T cell"[tiab] OR "Treg"[tiab] OR "regulatory T cell"[tiab] OR "Foxp3"[tiab] OR "MDSC"[tiab] OR "myeloid derived suppressor cell"[tiab] OR "macrophage"[tiab] OR "TAM"[tiab] OR "tumor associated macrophage"[tiab] OR "NK cell"[tiab] OR "natural killer cell"[tiab] OR "B cell"[tiab] OR "dendritic cell"[tiab] OR "immune cell infiltration"[tiab] OR "immune infiltrate"[tiab] OR "PD-1"[tiab] OR "PD1"[tiab] OR "PD-L1"[tiab] OR "PDL1"[tiab] OR "CTLA-4"[tiab] OR "CTLA4"[tiab] OR "LAG-3"[tiab] OR "LAG3"[tiab] OR "TIM-3"[tiab] OR "TIM3"[tiab] OR "TIGIT"[tiab] OR "VISTA"[tiab] OR "PRAME"[tiab] OR "Melan-A"[tiab] OR "MART-1"[tiab]) OR ("fibrosis"[tiab] OR "fibrotic"[tiab] OR "desmoplasia"[tiab] OR "angiogenesis"[tiab] OR "angiogenic"[tiab] OR "neovascularization"[tiab] OR "microvessel density"[tiab] OR "CD31"[tiab] OR "VEGF"[tiab] OR "vascular endothelial growth factor"[tiab] OR "extracellular matrix"[tiab] OR "ECM"[tiab] OR "collagen"[tiab] OR "alpha SMA"[tiab] OR "α-SMA"[tiab] OR "spatial analysis"[tiab] OR "spatial distribution"[tiab] OR "spatial organization"[tiab] OR "ultrastructure"[tiab] OR "electron microscopy"[tiab] OR "histopathology"[tiab] OR "immunohistochemistry"[tiab] OR "IHC"[tiab] OR "immunofluorescence"[tiab] OR "IF"[tiab] OR "hematoxylin and eosin"[tiab] OR "H&E"[tiab]

	OR "multiplex immunohistochemistry"[tiab] OR "multiplex immunofluorescence"[tiab] OR "mIHC"[tiab] OR "spatial transcriptomics"[tiab] OR "digital pathology"[tiab] OR "cytokine"[tiab] OR "chemokine"[tiab] OR "soluble mediator"[tiab] OR "IFN-gamma"[tiab] OR "IFNγ"[tiab] OR "interferon gamma"[tiab] OR "TNF-alpha"[tiab] OR "TNFα"[tiab] OR "tumor necrosis factor alpha"[tiab] OR "interleukin"[tiab] OR "IL-2"[tiab] OR "IL-4"[tiab] OR "IL-6"[tiab] OR "IL-10"[tiab] OR "IL-12"[tiab] OR "IL-17"[tiab] OR "TGF-beta"[tiab] OR "TGFβ"[tiab] OR "CCL2"[tiab] OR "CCL3"[tiab] OR "CCL4"[tiab] OR "CCL5"[tiab] OR "CCL22"[tiab] OR "CXCL9"[tiab] OR "CXCL10"[tiab] OR "soluble PD-L1"[tiab] OR "sPD-L1"[tiab] OR "soluble PD-1"[tiab] OR "sPD-1"[tiab] OR "LDH"[tiab] OR "lactate dehydrogenase"[tiab] OR "plasma"[tiab] OR "serum"[tiab] OR "peripheral blood"[tiab] OR "blood biomarker"[tiab] OR "multiplex assay"[tiab] OR "Luminex"[tiab] OR "ELISA"[tiab] OR "proximity extension assay"[tiab])) AND Humans[mh] AND ("Randomized Controlled Trial"[pt] OR "Clinical Trial"[pt] OR "Controlled Clinical Trial"[pt] OR "Observational Study"[pt]))
**Preclinical Studies**	(("Melanoma"[Mesh] OR "Melanoma, Experimental"[Mesh] OR melanoma*[tiab] OR "B16F10"[tiab] OR B16[tiab] OR "cutaneous melanoma"[tiab] OR "malignant melanoma"[tiab] OR "skin melanoma"[tiab] OR "melanoma metastasis"[tiab] OR "metastatic melanoma"[tiab]) AND ("Ipilimumab"[Mesh] OR "Nivolumab"[Mesh] OR "Pembrolizumab"[Mesh] OR "Immune Checkpoint Inhibitors"[Mesh] OR "CTLA-4 Antigen"[Mesh] OR "Programmed Cell Death 1 Receptor"[Mesh] OR ipilimumab[tiab] OR nivolumab[tiab] OR pembrolizumab[tiab] OR atezolizumab[tiab] OR avelumab[tiab] OR durvalumab[tiab] OR "anti-CTLA4"[tiab] OR "anti-CTLA-4"[tiab] OR "anti CTLA4"[tiab] OR "CTLA-4 inhibitor"[tiab] OR "CTLA4 inhibitor"[tiab] OR "anti-PD1"[tiab] OR "anti-PD-1"[tiab] OR "anti PD1"[tiab] OR "PD-1 inhibitor"[tiab] OR "PD1 inhibitor"[tiab] OR "anti-PD-L1"[tiab] OR "PD-L1 inhibitor"[tiab] OR "PDL1 inhibitor"[tiab] OR "immune checkpoint inhibitor"[tiab] OR "checkpoint inhibitor"[tiab] OR "checkpoint blockade"[tiab]) AND (("Tumor Microenvironment"[Mesh] OR "tumor microenvironment"[tiab] OR "tumour microenvironment"[tiab] OR "TME"[tiab] OR "spatial architecture"[tiab] OR "stromal remodeling"[tiab] OR "tumor stroma"[tiab] OR "tumor infiltrating lymphocyte"[tiab] OR "tumor-infiltrating lymphocyte"[tiab] OR "TIL"[tiab] OR "CD8"[tiab] OR "CD8+"[tiab] OR "CD3"[tiab] OR "cytotoxic T cell"[tiab] OR "CD4"[tiab] OR "CD4+"[tiab] OR "helper T cell"[tiab] OR "Treg"[tiab] OR "regulatory T cell"[tiab] OR "Foxp3"[tiab] OR "MDSC"[tiab] OR "myeloid derived suppressor cell"[tiab] OR "macrophage"[tiab] OR "TAM"[tiab] OR "tumor associated macrophage"[tiab] OR "NK cell"[tiab] OR "natural killer cell"[tiab] OR "B cell"[tiab] OR "dendritic cell"[tiab] OR "immune cell infiltration"[tiab] OR "immune infiltrate"[tiab] OR "PD-1"[tiab] OR "PD1"[tiab] OR "PD-L1"[tiab] OR "PDL1"[tiab] OR "CTLA-4"[tiab] OR "CTLA4"[tiab] OR "LAG-3"[tiab] OR "LAG3"[tiab] OR "TIM-3"[tiab] OR "TIM3"[tiab] OR "TIGIT"[tiab] OR "VISTA"[tiab] OR "PRAME"[tiab] OR "Melan-A"[tiab] OR "MART-1"[tiab]) OR ("fibrosis"[tiab] OR "fibrotic"[tiab] OR "desmoplasia"[tiab] OR "angiogenesis"[tiab]

	OR "angiogenic"[tiab] OR "neovascularization"[tiab] OR "microvessel density"[tiab] OR "CD31"[tiab] OR "VEGF"[tiab] OR "vascular endothelial growth factor"[tiab] OR "extracellular matrix"[tiab] OR "ECM"[tiab] OR "collagen"[tiab] OR "alpha SMA"[tiab] OR "α-SMA"[tiab] OR "spatial analysis"[tiab] OR "spatial distribution"[tiab] OR "spatial organization"[tiab] OR "ultrastructure"[tiab] OR "electron microscopy"[tiab] OR "histopathology"[tiab] OR "immunohistochemistry"[tiab] OR "IHC"[tiab] OR "immunofluorescence"[tiab] OR "IF"[tiab] OR "hematoxylin and eosin"[tiab] OR "H&E"[tiab] OR "multiplex immunohistochemistry"[tiab] OR "multiplex immunofluorescence"[tiab] OR "mIHC"[tiab] OR "spatial transcriptomics"[tiab] OR "digital pathology"[tiab] OR "cytokine"[tiab] OR "chemokine"[tiab] OR "soluble mediator"[tiab] OR "IFN-gamma"[tiab] OR "IFNγ"[tiab] OR "interferon gamma"[tiab] OR "TNF-alpha"[tiab] OR "TNFα"[tiab] OR "tumor necrosis factor alpha"[tiab] OR "interleukin"[tiab] OR "IL-2"[tiab] OR "IL-4"[tiab] OR "IL-6"[tiab] OR "IL-10"[tiab] OR "IL-12"[tiab] OR "IL-17"[tiab] OR "TGF-beta"[tiab] OR "TGFβ"[tiab] OR "CCL2"[tiab] OR "CCL3"[tiab] OR "CCL4"[tiab] OR "CCL5"[tiab] OR "CCL22"[tiab] OR "CXCL9"[tiab] OR "CXCL10"[tiab] OR "soluble PD-L1"[tiab] OR "sPD-L1"[tiab] OR "soluble PD-1"[tiab] OR "sPD-1"[tiab] OR "LDH"[tiab] OR "lactate dehydrogenase"[tiab] OR "plasma"[tiab] OR "serum"[tiab] OR "peripheral blood"[tiab] OR "blood biomarker"[tiab] OR "multiplex assay"[tiab] OR "Luminex"[tiab] OR "ELISA"[tiab] OR "proximity extension assay"[tiab])) AND Animals[mh] AND (mice[mh] OR "mouse"[tiab] OR "murine"[tiab]) AND ("B16"[tiab] OR "syngeneic"[tiab] OR "C57BL/6"[tiab] OR "YUMM"[tiab] OR "Yummer"[tiab] OR "RET"[tiab] OR "SM1"[tiab] OR "SM2"[tiab]))

B. SCOPUS/ELSEVIER

Search Type	SCOPUS/ELSEVIER Search String
Clinical Studies	TITLE-ABS-KEY (melanoma* OR "cutaneous melanoma" OR "malignant melanoma" OR "metastatic melanoma") AND TITLE-ABS-KEY (ipilimumab OR nivolumab OR pembrolizumab OR atezolizumab OR avelumab OR durvalumab OR "anti-ctla4" OR "anti-ctla-4" OR "anti ctla4" OR "ctla-4 inhibitor" OR "ctla4 inhibitor" OR "anti-pd1" OR "anti-pd-1" OR "anti pd1" OR "pd-1 inhibitor" OR "pd1 inhibitor" OR "anti-pd-l1" OR "pd-l1 inhibitor" OR "pdl1 inhibitor" OR "immune checkpoint inhibitor" OR "checkpoint inhibitor" OR "checkpoint blockade") AND (TITLE ("tumor microenvironment" OR "tumour microenvironment" OR "TME" OR "spatial architecture" OR "stromal remodeling" OR "tumor

	stroma" OR "tumor infiltrating lymphocyte" OR "tumor-infiltrating lymphocyte" OR "TIL" OR "CD8" OR "CD8+" OR "CD3" OR "cytotoxic T cell" OR "CD4" OR "CD4+" OR "helper T cell" OR "Treg" OR "regulatory T cell" OR "Foxp3" OR "MDSC" OR "myeloid derived suppressor cell" OR "macrophage" OR "TAM" OR "tumor associated macrophage" OR "NK cell" OR "natural killer cell" OR "B cell" OR "dendritic cell" OR "immune cell infiltration" OR "immune infiltrate") OR TITLE ("PD-1" OR "PD1" OR "PD-L1" OR "PDL1" OR "CTLA-4" OR "CTLA4" OR "LAG-3" OR "LAG3" OR "TIM-3" OR "TIM3" OR "TIGIT" OR "VISTA" OR "PRAME" OR "Melan-A" OR "MART-1") OR TITLE ("fibrosis" OR "fibrotic" OR "desmoplasia" OR "angiogenesis" OR "angiogenic" OR "neovascularization" OR "microvessel density" OR "CD31" OR "VEGF" OR "vascular endothelial growth factor" OR "extracellular matrix" OR "ECM" OR "collagen" OR "alpha SMA" OR "α-SMA" OR "spatial analysis" OR "spatial distribution" OR "spatial organization" OR "ultrastructure" OR "electron microscopy" OR "histopathology" OR "immunohistochemistry" OR "IHC" OR "immunofluorescence" OR "IF" OR "hematoxylin and eosin" OR "H&E" OR "multiplex immunohistochemistry" OR "multiplex immunofluorescence" OR "mIHC" OR "spatial transcriptomics" OR "digital pathology") OR TITLE ("cytokine" OR "chemokine" OR "soluble mediator" OR "IFN-gamma" OR "IFNγ" OR "interferon gamma" OR "TNF-alpha" OR "TNFα" OR "tumor necrosis factor alpha" OR "interleukin" OR "IL-2" OR "IL-4" OR "IL-6" OR "IL-10" OR "IL-12" OR "IL-17" OR "TGF-beta" OR "TGFβ" OR "CCL2" OR "CCL3" OR "CCL4" OR "CCL5" OR "CCL22" OR "CXCL9" OR "CXCL10" OR "soluble PD-L1" OR "sPD-L1" OR "soluble PD-1" OR "sPD-1" OR "LDH" OR "lactate dehydrogenase" OR "plasma" OR "serum" OR "peripheral blood" OR "blood biomarker" OR "multiplex assay" OR "Luminex" OR "ELISA" OR "proximity extension assay")) AND ABS ("melanoma" AND ("tumor microenvironment" OR "immunotherapy" OR "checkpoint inhibitor" OR "clinical trial") AND ("patient" OR "patients" OR "human")) AND TITLE-ABS-KEY ("biomarker" OR "predictive" OR "prognostic" OR "correlative" OR "translational") AND NOT TITLE-ABS-KEY (mouse OR mice OR murine OR rat OR rodent OR animal OR b16 OR syngeneic OR "c57bl/6" OR "animal model" OR "murine model" OR xenograft) AND LIMIT-TO (SUBJAREA , "MEDI") AND LIMIT-TO (DOCTYPE , "ar") AND LIMIT-TO (LANGUAGE , "English")
**Preclinical Studies**	TITLE-ABS-KEY (melanoma* OR "cutaneous melanoma" OR "malignant melanoma" OR "skin melanoma" OR "melanoma metastasis" OR "metastatic melanoma" OR "B16F10" OR "B16" OR "YUMM" OR "Yummer" OR "RET" OR "SM1" OR "SM2" OR "syngeneic" OR "C57BL/6") AND TITLE-ABS-KEY (ipilimumab OR nivolumab OR pembrolizumab OR atezolizumab OR avelumab OR durvalumab OR "anti-ctla4" OR "anti-ctla-4" OR "anti ctla4" OR "ctla-4 inhibitor" OR "ctla4 inhibitor" OR "anti-pd1" OR "anti-pd-1" OR "anti pd1" OR "pd-1 inhibitor" OR "pd1 inhibitor" OR "anti-pd-l1" OR "pd-l1 inhibitor" OR "pdl1 inhibitor" OR "immune checkpoint inhibitor" OR "checkpoint inhibitor" OR "checkpoint blockade") AND (TITLE ("tumor microenvironment" OR "tumour microenvironment" OR "TME" OR "spatial architecture" OR

	"stromal remodeling" OR "tumor stroma" OR "tumor infiltrating lymphocyte" OR "tumor-infiltrating lymphocyte" OR "TIL" OR "CD8" OR "CD8+" OR "CD3" OR "cytotoxic T cell" OR "CD4" OR "CD4+" OR "helper T cell" OR "Treg" OR "regulatory T cell" OR "Foxp3" OR "MDSC" OR "myeloid derived suppressor cell" OR "macrophage" OR "TAM" OR "tumor associated macrophage" OR "NK cell" OR "natural killer cell" OR "B cell" OR "dendritic cell" OR "immune cell infiltration" OR "immune infiltrate" OR "PD-1" OR "PD1" OR "PD-L1" OR "PDL1" OR "CTLA-4" OR "CTLA4" OR "LAG-3" OR "LAG3" OR "TIM-3" OR "TIM3" OR "TIGIT" OR "VISTA" OR "PRAME" OR "Melan-A" OR "MART-1" OR "fibrosis" OR "fibrotic" OR "desmoplasia" OR "angiogenesis" OR "angiogenic" OR "neovascularization" OR "microvessel density" OR "CD31" OR "VEGF" OR "vascular endothelial growth factor" OR "extracellular matrix" OR "ECM" OR "collagen" OR "alpha SMA" OR "α-SMA" OR "spatial analysis" OR "spatial distribution" OR "spatial organization" OR "ultrastructure" OR "electron microscopy" OR "histopathology" OR "immunohistochemistry" OR "IHC" OR "immunofluorescence" OR "IF" OR "hematoxylin and eosin" OR "H&E" OR "multiplex immunohistochemistry" OR "multiplex immunofluorescence" OR "mIHC" OR "spatial transcriptomics" OR "digital pathology" OR "cytokine" OR "chemokine" OR "soluble mediator" OR "IFN-gamma" OR "IFNγ" OR "interferon gamma" OR "TNF-alpha" OR "TNFα" OR "tumor necrosis factor alpha" OR "interleukin" OR "IL-2" OR "IL-4" OR "IL-6" OR "IL-10" OR "IL-12" OR "IL-17" OR "TGF-beta" OR "TGFβ" OR "CCL2" OR "CCL3" OR "CCL4" OR "CCL5" OR "CCL22" OR "CXCL9" OR "CXCL10" OR "soluble PD-L1" OR "sPD-L1" OR "soluble PD-1" OR "sPD-1" OR "LDH" OR "lactate dehydrogenase" OR "plasma" OR "serum" OR "peripheral blood" OR "blood biomarker" OR "multiplex assay" OR "Luminex" OR "ELISA" OR "proximity extension assay") OR ABS ("tumor microenvironment" OR "tumour microenvironment" OR "TME" OR "spatial architecture" OR "stromal remodeling" OR "tumor stroma" OR "tumor infiltrating lymphocyte" OR "tumor-infiltrating lymphocyte" OR "TIL" OR "CD8" OR "CD8+" OR "CD3" OR "cytotoxic T cell" OR "CD4" OR "CD4+" OR "helper T cell" OR "Treg" OR "regulatory T cell" OR "Foxp3" OR "MDSC" OR "myeloid derived suppressor cell" OR "macrophage" OR "TAM" OR "tumor associated macrophage" OR "NK cell" OR "natural killer cell" OR "B cell" OR "dendritic cell" OR "immune cell infiltration" OR "immune infiltrate" OR "PD-1" OR "PD1" OR "PD-L1" OR "PDL1" OR "CTLA-4" OR "CTLA4" OR "LAG-3" OR "LAG3" OR "TIM-3" OR "TIM3" OR "TIGIT" OR "VISTA" OR "PRAME" OR "Melan-A" OR "MART-1" OR "fibrosis" OR "fibrotic" OR "desmoplasia" OR "angiogenesis" OR "angiogenic" OR "neovascularization" OR "microvessel density" OR "CD31" OR "VEGF" OR "vascular endothelial growth factor" OR "extracellular matrix" OR "ECM" OR "collagen" OR "alpha SMA" OR "α-SMA" OR "spatial analysis" OR "spatial distribution" OR "spatial organization" OR "ultrastructure" OR "electron microscopy" OR "histopathology" OR "immunohistochemistry" OR "IHC" OR "immunofluorescence" OR "IF" OR "hematoxylin and eosin" OR "H&E" OR "multiplex immunohistochemistry" OR "multiplex immunofluorescence" OR "mIHC" OR "spatial transcriptomics" OR "digital pathology" OR "cytokine" OR "chemokine" OR "soluble mediator" OR "IFN-gamma" OR "IFNγ" OR "interferon gamma" OR "TNF-alpha" OR "TNFα" OR

	"tumor necrosis factor alpha" OR "interleukin" OR "IL-2" OR "IL-4" OR "IL-6" OR "IL-10" OR "IL-12" OR "IL-17" OR "TGF-beta" OR "TGFβ" OR "CCL2" OR "CCL3" OR "CCL4" OR "CCL5" OR "CCL22" OR "CXCL9" OR "CXCL10" OR "soluble PD-L1" OR "sPD-L1" OR "soluble PD-1" OR "sPD-1" OR "LDH" OR "lactate dehydrogenase" OR "plasma" OR "serum" OR "peripheral blood" OR "blood biomarker" OR "multiplex assay" OR "Luminex" OR "ELISA" OR "proximity extension assay")) AND ABS ("mouse" OR "mice" OR "murine" OR "B16" OR "syngeneic" OR "C57BL/6") AND NOT TITLE-ABS-KEY (human OR patient OR clinical OR trial OR "clinical trial" OR "phase" OR "cohort" OR "randomized" OR "observational") AND (LIMIT-TO (SUBJAREA , "MEDI") OR LIMIT-TO (SUBJAREA , "BIOC") OR LIMIT-TO (SUBJAREA , "IMMU") OR LIMIT-TO (SUBJAREA , "PHAR") OR LIMIT-TO (SUBJAREA , "CENG") OR LIMIT-TO (SUBJAREA , "MULT")) AND LIMIT-TO (DOCTYPE , "ar") AND LIMIT-TO (LANGUAGE , "English")

C. WEB OF SCIENCE

Search Type	WEB OF SCIENCE Search String
Clinical Studies	TI=(melanoma* OR "cutaneous melanoma" OR "malignant melanoma" OR "skin melanoma" OR "melanoma metastasis" OR "metastatic melanoma") AND (TI=(ipilimumab OR nivolumab OR pembrolizumab OR "anti-CTLA4" OR "anti-CTLA-4" OR "anti CTLA4" OR "CTLA-4 inhibitor" OR "anti-PD1" OR "anti-PD-1" OR "anti PD1" OR "PD-1 inhibitor" OR "anti-PD-L1" OR "PD-L1 inhibitor" OR "immune checkpoint inhibitor" OR "checkpoint inhibitor" OR "checkpoint blockade") OR AB=(ipilimumab OR nivolumab OR pembrolizumab OR "anti-CTLA4" OR "anti-CTLA-4" OR "anti CTLA4" OR "CTLA-4 inhibitor" OR "anti-PD1" OR "anti-PD-1" OR "anti PD1" OR "PD-1 inhibitor" OR "anti-PD-L1" OR "PD-L1 inhibitor" OR "immune checkpoint inhibitor" OR "checkpoint inhibitor" OR "checkpoint blockade")) AND (TI=("tumor microenvironment" OR "tumour microenvironment" OR "TME" OR "spatial architecture" OR "stromal remodeling" OR "tumor stroma" OR "tumor infiltrating lymphocyte" OR "tumor-infiltrating lymphocyte" OR "TIL") OR TI=("CD8" OR "CD8+" OR "CD3" OR "cytotoxic T cell" OR "CD4" OR "CD4+" OR "helper T cell" OR "Treg" OR "regulatory T cell" OR "Foxp3") OR TI=("MDSC" OR "myeloid derived suppressor cell" OR "macrophage" OR "TAM" OR "tumor associated macrophage" OR "NK cell" OR "natural killer cell" OR "B cell" OR "dendritic cell" OR "immune cell infiltration" OR "immune infiltrate") OR TI=("PD-1" OR "PD1" OR "PD-L1" OR "PDL1" OR "CTLA-4" OR "CTLA4" OR "LAG-3" OR "LAG3"

	OR "TIM-3" OR "TIM3" OR "TIGIT" OR "VISTA" OR "PRAME" OR "Melan-A" OR "MART-1") OR TI=("fibrosis" OR "fibrotic" OR "desmoplasia" OR "angiogenesis" OR "angiogenic" OR "neovascularization" OR "microvessel density" OR "CD31" OR "VEGF" OR "vascular endothelial growth factor") OR TI=("extracellular matrix" OR "ECM" OR "collagen" OR "alpha SMA" OR "α-SMA" OR "spatial analysis" OR "spatial distribution" OR "spatial organization") OR TI=("ultrastructure" OR "electron microscopy" OR "histopathology" OR "immunohistochemistry" OR "IHC" OR "immunofluorescence" OR "IF") OR TI=("hematoxylin and eosin" OR "H&E" OR "multiplex immunohistochemistry" OR "multiplex immunofluorescence" OR "mIHC" OR "spatial transcriptomics" OR "digital pathology") OR TI=("cytokine" OR "chemokine" OR "soluble mediator" OR "IFN-gamma" OR "IFNγ" OR "interferon gamma" OR "TNF-alpha" OR "TNFα" OR "tumor necrosis factor alpha") OR TI=("interleukin" OR "IL-2" OR "IL-4" OR "IL-6" OR "IL-10" OR "IL-12" OR "IL-17" OR "TGF-beta" OR "TGFβ") OR TI=("CCL2" OR "CCL3" OR "CCL4" OR "CCL5" OR "CCL22" OR "CXCL9" OR "CXCL10" OR "soluble PD-L1" OR "sPD-L1" OR "soluble PD-1" OR "sPD-1") OR TI=("LDH" OR "lactate dehydrogenase" OR "plasma" OR "serum" OR "peripheral blood" OR "blood biomarker" OR "multiplex assay" OR "Luminex" OR "ELISA" OR "proximity extension assay"))<br><br>AND DT=(Article) AND LA=(English) AND WC=(Oncology OR Immunology) AND (TI=("clinical trial" OR "phase II" OR "phase III" OR "phase 2" OR "phase 3" OR "randomized" OR "prospective" OR "cohort") OR AB=("clinical trial" OR patient OR patients OR cohort OR "phase II" OR "phase III" OR "phase 2" OR "phase 3" OR "randomized" OR "prospective" OR "clinical study")) NOT (TI=(mouse OR mice OR murine OR rat OR rodent OR animal OR B16 OR syngeneic OR "C57BL/6" OR xenograft OR "in vivo") OR AB=(mouse OR mice OR murine OR rat OR rodent OR animal OR B16 OR syngeneic OR "C57BL/6" OR xenograft OR "in vivo")) NOT DT=(Meeting Abstract OR Editorial Material OR Letter OR Book Chapter OR Note OR Reprint OR Retracted Publication)
**Preclinical Studies**	TI=(melanoma* OR "cutaneous melanoma" OR "malignant melanoma" OR "skin melanoma" OR "melanoma metastasis" OR "metastatic melanoma" OR "B16F10" OR "B16" OR "YUMM" OR "Yummer" OR "RET" OR "SM1" OR "SM2") AND (TI=(ipilimumab OR nivolumab OR pembrolizumab OR "anti-CTLA4" OR "anti-CTLA-4" OR "anti CTLA4" OR "CTLA-4 inhibitor" OR "anti-PD1" OR "anti-PD-1" OR "anti PD1" OR "PD-1 inhibitor" OR "anti-PD-L1" OR "PD-L1 inhibitor" OR "immune checkpoint inhibitor" OR "checkpoint inhibitor" OR "checkpoint blockade") OR AB=(ipilimumab OR nivolumab OR pembrolizumab OR "anti-CTLA4" OR "anti-CTLA-4" OR "anti CTLA4" OR "CTLA-4 inhibitor" OR "anti-PD1" OR "anti-PD-1" OR "anti PD1" OR "PD-1 inhibitor" OR "anti-PD-L1" OR "PD-L1 inhibitor" OR "immune checkpoint inhibitor" OR "checkpoint inhibitor" OR "checkpoint blockade")) AND (TI=("tumor microenvironment" OR "tumour microenvironment" OR "TME" OR "spatial architecture" OR "stromal remodeling" OR "tumor stroma" OR "tumor infiltrating lymphocyte" OR "tumor-infiltrating

	lymphocyte" OR "TIL" OR "CD8" OR "CD8+" OR "CD3" OR "cytotoxic T cell" OR "CD4" OR "CD4+" OR "helper T cell" OR "Treg" OR "regulatory T cell" OR "Foxp3" OR "MDSC" OR "myeloid derived suppressor cell" OR "macrophage" OR "TAM" OR "tumor associated macrophage" OR "NK cell" OR "natural killer cell" OR "B cell" OR "dendritic cell" OR "immune cell infiltration" OR "immune infiltrate" OR "PD-1" OR "PD1" OR "PD-L1" OR "PDL1" OR "CTLA-4" OR "CTLA4" OR "LAG-3" OR "LAG3" OR "TIM-3" OR "TIM3" OR "TIGIT" OR "VISTA" OR "PRAME" OR "Melan-A" OR "MART-1" OR "fibrosis" OR "fibrotic" OR "desmoplasia" OR "angiogenesis" OR "angiogenic" OR "neovascularization" OR "microvessel density" OR "CD31" OR "VEGF" OR "vascular endothelial growth factor" OR "extracellular matrix" OR "ECM" OR "collagen" OR "alpha SMA" OR "α-SMA" OR "spatial analysis" OR "spatial distribution" OR "spatial organization" OR "ultrastructure" OR "electron microscopy" OR "histopathology" OR "immunohistochemistry" OR "IHC" OR "immunofluorescence" OR "IF" OR "hematoxylin and eosin" OR "H&E" OR "multiplex immunohistochemistry" OR "multiplex immunofluorescence" OR "mIHC" OR "spatial transcriptomics" OR "digital pathology" OR "cytokine" OR "chemokine" OR "soluble mediator" OR "IFN-gamma" OR "IFNγ" OR "interferon gamma" OR "TNF-alpha" OR "TNFα" OR "tumor necrosis factor alpha" OR "interleukin" OR "IL-2" OR "IL-4" OR "IL-6" OR "IL-10" OR "IL-12" OR "IL-17" OR "TGF-beta" OR "TGFβ" OR "CCL2" OR "CCL3" OR "CCL4" OR "CCL5" OR "CCL22" OR "CXCL9" OR "CXCL10" OR "soluble PD-L1" OR "sPD-L1" OR "soluble PD-1" OR "sPD-1" OR "LDH" OR "lactate dehydrogenase" OR "plasma" OR "serum" OR "peripheral blood" OR "blood biomarker" OR "multiplex assay" OR "Luminex" OR "ELISA" OR "proximity extension assay") OR AB=("tumor microenvironment" OR "tumour microenvironment" OR "TME" OR "spatial architecture" OR "stromal remodeling" OR "tumor stroma" OR "tumor infiltrating lymphocyte" OR "tumor-infiltrating lymphocyte" OR "TIL" OR "CD8" OR "CD8+" OR "CD3" OR "cytotoxic T cell" OR "CD4" OR "CD4+" OR "helper T cell" OR "Treg" OR "regulatory T cell" OR "Foxp3" OR "MDSC" OR "myeloid derived suppressor cell" OR "macrophage" OR "TAM" OR "tumor associated macrophage" OR "NK cell" OR "natural killer cell" OR "B cell" OR "dendritic cell" OR "immune cell infiltration" OR "immune infiltrate" OR "PD-1" OR "PD1" OR "PD-L1" OR "PDL1" OR "CTLA-4" OR "CTLA4" OR "LAG-3" OR "LAG3" OR "TIM-3" OR "TIM3" OR "TIGIT" OR "VISTA" OR "PRAME" OR "Melan-A" OR "MART-1" OR "fibrosis" OR "fibrotic" OR "desmoplasia" OR "angiogenesis" OR "angiogenic" OR "neovascularization" OR "microvessel density" OR "CD31" OR "VEGF" OR "vascular endothelial growth factor" OR "extracellular matrix" OR "ECM" OR "collagen" OR "alpha SMA" OR "α-SMA" OR "spatial analysis" OR "spatial distribution" OR "spatial organization" OR "ultrastructure" OR "electron microscopy" OR "histopathology" OR "immunohistochemistry" OR "IHC" OR "immunofluorescence" OR "IF" OR "hematoxylin and eosin" OR "H&E" OR "multiplex immunohistochemistry" OR "multiplex immunofluorescence" OR "mIHC" OR "spatial transcriptomics" OR "digital pathology" OR "cytokine" OR "chemokine" OR "soluble mediator" OR "IFN-gamma" OR "IFNγ" OR "interferon gamma" OR "TNF-alpha" OR "TNFα" OR "tumor necrosis factor alpha" OR "interleukin" OR "IL-

	2" OR "IL-4" OR "IL-6" OR "IL-10" OR "IL-12" OR "IL-17" OR "TGF-beta" OR "TGFβ" OR "CCL2" OR "CCL3" OR "CCL4" OR "CCL5" OR "CCL22" OR "CXCL9" OR "CXCL10" OR "soluble PD-L1" OR "sPD-L1" OR "soluble PD-1" OR "sPD-1" OR "LDH" OR "lactate dehydrogenase" OR "plasma" OR "serum" OR "peripheral blood" OR "blood biomarker" OR "multiplex assay" OR "Luminex" OR "ELISA" OR "proximity extension assay")) AND AB=("mouse" OR "mice" OR "murine" OR "B16" OR "syngeneic" OR "C57BL/6" OR "animal model" OR "in vivo") AND DT=(Article) AND LA=(English) NOT (TI=("clinical trial" OR "phase II" OR "phase III" OR "phase 2" OR "phase 3" OR "randomized" OR "prospective" OR "cohort" OR "clinical study") AND TI=(patient OR patients OR human)) NOT DT=(Meeting Abstract OR Editorial Material OR Letter OR Book Chapter OR Note OR Reprint OR Retracted Publication)

D. COCHRANE LIBRARY

Search Type	COCHRANE LIBRRY Search String (Final, Executable)
Clinical Studies	#1: melanoma* OR "cutaneous melanoma" OR "malignant melanoma" OR "skin melanoma" OR "melanoma metastasis" OR "metastatic melanoma"<br><br>#2: ipilimumab OR nivolumab OR pembrolizumab OR atezolizumab OR avelumab OR durvalumab OR "anti-CTLA4" OR "anti-CTLA-4" OR "anti CTLA4" OR "CTLA-4 inhibitor" OR "CTLA4 inhibitor" OR "anti-PD1" OR "anti-PD-1" OR "anti PD1" OR "PD-1 inhibitor" OR "PD1 inhibitor" OR "anti-PD-L1" OR "PD-L1 inhibitor" OR "PDL1 inhibitor" OR "immune checkpoint inhibitor" OR "checkpoint inhibitor" OR "checkpoint blockade"<br><br>#3: "tumor microenvironment" OR "tumour microenvironment" OR "TME" OR "spatial architecture" OR "stromal remodeling" OR "tumor stroma" OR "tumor infiltrating lymphocyte" OR "tumor-infiltrating lymphocyte" OR "TIL" OR "CD8" OR "CD8+" OR "CD3" OR "cytotoxic T

	cell" OR "CD4" OR "CD4+" OR "helper T cell" OR "Treg" OR "regulatory T cell" OR "Foxp3" OR "MDSC" OR "myeloid derived suppressor cell" OR "macrophage" OR "TAM" OR "tumor associated macrophage" OR "NK cell" OR "natural killer cell" OR "B cell" OR "dendritic cell" OR "immune cell infiltration" OR "immune infiltrate" OR "PD-1" OR "PD1" OR "PD-L1" OR "PDL1" OR "CTLA-4" OR "CTLA4" OR "LAG-3" OR "LAG3" OR "TIM-3" OR "TIM3" OR "TIGIT" OR "VISTA" OR "PRAME" OR "Melan-A" OR "MART-1" OR "fibrosis" OR "fibrotic" OR "desmoplasia" OR "angiogenesis" OR "angiogenic" OR "neovascularization" OR "microvessel density" OR "CD31" OR "VEGF" OR "vascular endothelial growth factor" OR "extracellular matrix" OR "ECM" OR "collagen" OR "alpha SMA" OR "alpha-SMA" OR "spatial analysis" OR "spatial distribution" OR "spatial organization" OR "ultrastructure" OR "electron microscopy" OR "histopathology" OR "immunohistochemistry" OR "IHC" OR "immunofluorescence" OR "IF" OR "hematoxylin and eosin" OR "H&E" OR "multiplex immunohistochemistry" OR "multiplex immunofluorescence" OR "mIHC" OR "spatial transcriptomics" OR "digital pathology"<br><br>#4: "cytokine" OR "chemokine" OR "soluble mediator" OR "IFN-gamma" OR "IFN gamma" OR "interferon gamma" OR "TNF-alpha" OR "TNF alpha" OR "tumor necrosis factor alpha" OR "interleukin" OR "IL-2" OR "IL-4" OR "IL-6" OR "IL-10" OR "IL-12" OR "IL-17" OR "TGF-beta" OR "TGF beta" OR "CCL2" OR "CCL3" OR "CCL4" OR "CCL5" OR "CCL22" OR "CXCL9" OR "CXCL10" OR "soluble PD-L1" OR "sPD-L1" OR "soluble PD-1" OR "sPD-1" OR "LDH" OR "lactate dehydrogenase" OR "plasma" OR "serum" OR "peripheral blood" OR "blood biomarker" OR "multiplex assay" OR "Luminex" OR "ELISA" OR "proximity extension assay"<br><br>#5: #1 AND #2 AND (#3 OR #4)<br><br>#6: "patient" OR "patients" OR "human subjects" OR "clinical study" OR "clinical trial" OR "randomized controlled trial" OR "controlled clinical trial"<br><br>#7: "advanced melanoma" OR "unresectable melanoma" OR "stage III" OR "stage IV"<br><br>#8: #5 AND #6 AND #7<br><br>#9: "treatment"<br><br>#10: #8 AND #9

	LIMITS APPLIED:<br><br>- Content type: Trials<br><br>- Search word variations: Disabled<br><br>- Language: English (via protocol, though not filterable in interface)
**Preclinical Studies**	#1: melanoma* OR "cutaneous melanoma" OR "malignant melanoma" OR "skin melanoma" OR "melanoma metastasis" OR "metastatic melanoma" OR "B16F10" OR B16 OR "YUMM" OR "Yummer" OR "RET" OR "SM1" OR "SM2" OR "syngeneic" OR "C57BL/6"#2: ipilimumab OR nivolumab OR pembrolizumab OR atezolizumab OR avelumab OR durvalumab OR "anti-CTLA4" OR "anti-CTLA-4" OR "anti CTLA4" OR "CTLA-4 inhibitor" OR "CTLA4 inhibitor" OR "anti-PD1" OR "anti-PD-1" OR "anti PD1" OR "PD-1 inhibitor" OR "PD1 inhibitor" OR "anti-PD-L1" OR "PD-L1 inhibitor" OR "PDL1 inhibitor" OR "immune checkpoint inhibitor" OR "checkpoint inhibitor" OR "checkpoint blockade"#3: "tumor microenvironment" OR "tumour microenvironment" OR "TME" OR "spatial architecture" OR "stromal remodeling" OR "tumor stroma" OR "tumor infiltrating lymphocyte" OR "tumor-infiltrating lymphocyte" OR "TIL" OR "CD8" OR "CD8+" OR "CD3" OR "cytotoxic T cell" OR "CD4" OR "CD4+" OR "helper T cell" OR "Treg" OR "regulatory T cell" OR "Foxp3" OR "MDSC" OR "myeloid derived suppressor cell" OR "macrophage" OR "TAM" OR "tumor associated macrophage" OR "NK cell" OR "natural killer cell" OR "B cell" OR "dendritic cell" OR "immune cell infiltration" OR "immune infiltrate" OR "PD-1" OR "PD1" OR "PD-L1" OR "PDL1" OR "CTLA-4" OR "CTLA4" OR "LAG-3" OR "LAG3" OR "TIM-3" OR "TIM3" OR "TIGIT" OR "VISTA" OR "PRAME" OR "Melan-A" OR "MART-1" OR "fibrosis" OR "fibrotic" OR "desmoplasia" OR "angiogenesis" OR "angiogenic" OR "neovascularization" OR "microvessel density" OR "CD31" OR "VEGF" OR "vascular endothelial growth factor" OR "extracellular matrix" OR "ECM" OR "collagen" OR "alpha SMA" OR "alpha-SMA" OR "spatial analysis" OR "spatial distribution" OR "spatial organization" OR "ultrastructure" OR "electron microscopy" OR "histopathology" OR "immunohistochemistry" OR "IHC" OR "immunofluorescence" OR "IF" OR "hematoxylin and eosin" OR "H&E" OR "multiplex immunohistochemistry" OR "multiplex immunofluorescence" OR "mIHC" OR "spatial transcriptomics" OR "digital pathology"#4: "cytokine" OR "chemokine" OR "soluble mediator" OR "IFN-gamma" OR "IFN gamma" OR "interferon gamma" OR "TNF-alpha" OR "TNF alpha" OR "tumor necrosis factor alpha" OR "interleukin" OR "IL-2" OR "IL-4" OR "IL-6" OR "IL-10" OR "IL-12" OR "IL-17" OR "TGF-beta" OR "TGF beta"

	OR "CCL2" OR "CCL3" OR "CCL4" OR "CCL5" OR "CCL22" OR "CXCL9" OR "CXCL10" OR "soluble PD-L1" OR "sPD-L1" OR "soluble PD-1" OR "sPD-1" OR "LDH" OR "lactate dehydrogenase" OR "plasma" OR "serum" OR "peripheral blood" OR "blood biomarker" OR "multiplex assay" OR "Luminex" OR "ELISA" OR "proximity extension assay"#5: #1 AND #2 AND (#3 OR #4) AND ("TME" OR "metastatic melanoma" OR "animal model" OR "murine model" OR "in vivo" OR "preclinical" OR "syngeneic" OR "translational" OR "B16" OR "B16F10")<br><br>LIMITS APPLIED TO LINE #5:<br><br>-Content type: Trials<br><br>-Search word variations: Disabled<br><br>-Language: English (via protocol)

Supplementary Table S2. **Curated meta-analytic dataset: individual study means, standard deviations, sample sizes, drug-class assignments, and comparators used for the quantitative synthesis (preclinical and clinical arms). [17–55, 63–66, 70, 73–84, 86, 87, 93, 105, 107, 108, 114, 118, 119, 122].**

A. PRECLINICAL STUDIES

Study	Parameter	n_treatment	mean_treatment	sd_treatment	n_control	mean_control	sd_control	Drug_Class
Amaro 2023	CD8	9	4.55	0.75	9	3.12	0.43	anti-CTLA-4
Amaro 2023	CD8	9	3.09	1.15	9	3.12	0.43	anti-PD-1
Amaro 2023	IFN-γ	9	1.97	1.43	9	3.94	1.43	anti-CTLA-4
Amaro 2023	IFN-γ	9	1.97	0.90	9	3.94	1.43	anti-PD-1
Amaro 2023	PD-L1	9	0.10	0.09	9	0.38	0.28	anti-CTLA-4
Amaro 2023	PD-L1	9	0.21	0.10	9	0.38	0.28	anti-PD-1
Ando 2021	CD8	4	23.31	1.72	4	18.92	0.67	anti-PD-1
Ando 2021	CD8/Treg	4	32.33	23.10	4	25.78	17.40	anti-PD-1
Antons 2025	CD8	18	153.66	149.96	22	48.78	65.04	anti-CTLA-4+anti-PD-L1
Antons 2025	Ki67	18	32.07	14.43	22	59.38	25.87	anti-CTLA-4+anti-PD-L1
Antons 2025	Apoptosis	18	56.58	17.35	22	22.67	13.19	anti-CTLA-4+anti-PD-L1
Benitez 2020	CD8	9	13.01	10.20	9	2.55	4.74	anti-CTLA-4
Benitez 2020	CD8/Treg	9	4.83	3.63	9	0.50	1.26	anti-CTLA-4
Buss 2021	CD8	10	42.57	33.50	10	22.01	14.18	anti-PD-1
Buss 2021	CD8/Treg	10	3.58	3.52	10	4.98	7.37	anti-PD-1
Capaccione 2022	Ki67	12	1.91	1.33	12	4.01	2.86	anti-CTLA-4+anti-PD-1
Capaccione 2022	Apoptosis	12	0.54	0.54	12	0.00	0.00	anti-CTLA-4+anti-PD-1
Chen 2023	CD8	5	11.19	0.98	5	6.77	0.76	anti-PD-1
Chen 2023	IFN-γ	5	33.18	3.23	5	9.39	3.42	anti-PD-1
Chen 2023	Ki67	5	7.66	1.43	5	2.82	1.26	anti-PD-1
Cheng 2025	CD8	6	46.84	10.98	6	26.11	1.87	anti-PD-L1

Cheng 2025	IFN-γ	6	144.97	7.69	6	92.31	10.06	anti-PD-L1
Cheng 2025	PD-L1	6	95.20	7.20	6	99.20	0.40	anti-PD-L1
Choi 2018	Apoptosis	10	33.06	5.54	8	5.74	2.32	anti-CTLA-4+anti-PD-L1
Choi 2018	Apoptosis	10	36.61	7.17	8	5.74	2.32	anti-CTLA-4+anti-PD-1
Dupuychaff 2025	CD8	6	15949.00	7442.00	6	26329.00	14882.00	anti-PD-1
Freimark 2016	CD8	17	7.38	2.25	17	5.01	2.04	anti-PD-1
Freimark 2016	CD8/Treg	17	16.04	18.64	17	37.59	49.27	anti-PD-1
Freimark 2016	IFN-γ	17	15.46	6.80	17	9.20	6.58	anti-PD-1
Fukuda 2021	CD8	9	43.78	53.73	9	33.83	35.82	anti-PD-1
Fukuda 2021	CD8/Treg	9	8.09	15.33	9	8.79	25.81	anti-PD-1
Fukuda 2021	IFN-γ	9	21.63	8.04	9	17.80	10.32	anti-PD-1
Guo 2022	CD8	5	35.56	2.34	5	25.73	3.27	anti-PD-1
Guo 2022	IFN-γ	5	25.35	6.05	5	8.14	3.72	anti-PD-1
Guo 2023	IFN-γ	6	1.00	0.27	6	0.21	0.10	anti-PD-L1
Hartley 2018	CD8	4	1.09	0.36	4	0.16	0.10	anti-PD-L1
Heimer 2025	CD8	8	395.40	522.50	8	71.90	55.60	anti-CTLA-4+anti-PD-L1
Heimer 2025	Ki67	8	27.20	9.70	8	58.70	8.60	anti-CTLA-4+anti-PD-L1
Heimer 2025	Apoptosis	8	52.30	17.20	8	17.10	5.60	anti-CTLA-4+anti-PD-L1
Herr 2025	CD8	8	395.40	522.50	8	71.90	55.60	anti-CTLA-4+anti-PD-L1
Herr 2025	Ki67	8	27.20	9.70	8	58.70	8.60	anti-CTLA-4+anti-PD-L1
Herr 2025	Apoptosis	8	52.30	17.20	8	17.10	5.60	anti-CTLA-4+anti-PD-L1
Hosoi 2018	CD8	5	0.42	0.31	5	0.07	0.02	anti-PD-1
Hosoi 2018	CD8	5	0.28	0.26	5	0.07	0.02	anti-CTLA-4
Hosoi 2018	IFN-γ	5	0.34	0.34	5	0.03	0.03	anti-PD-1
Hosoi 2018	IFN-γ	5	0.24	0.20	5	0.03	0.03	anti-CTLA-4
Hsu 2021	CD8	6	164.47	4.94	6	99.23	4.94	anti-PD-L1

**Hsu 2021**	CD8/Treg	6	2.79	0.19	6	0.99	0.06	anti-PD-L1
**Hsu 2021**	IFN-γ	6	186.70	4.79	6	98.94	6.91	anti-PD-L1
**Hsu 2021**	Ki67	6	78.69	2.81	6	99.77	3.86	anti-PD-L1
**Hsu 2021**	Apoptosis	6	146.56	3.13	6	98.33	3.76	anti-PD-L1
**Hu 2021**	CD8	5	0.61	0.54	5	0.61	0.27	anti-PD-1
**Hu 2021**	CD8/Treg	5	0.41	0.43	5	0.71	0.47	anti-PD-1
**Huang 2021**	CD8	5	7.01	5.77	5	4.40	3.74	anti-PD-1
**Huang 2021**	CD8/Treg	5	0.32	0.43	5	0.36	0.63	anti-PD-1
**Huang 2024**	IFN-γ	5	5.10	0.41	5	1.36	0.26	anti-PD-1
**Iannone 2014**	CD8	10	0.56	0.45	10	0.40	0.36	anti-CTLA-4
**Iannone 2014**	CD8/Treg	10	3.08	2.67	10	2.14	1.64	anti-CTLA-4
**Iannone 2014**	IFN-γ	10	3.01	1.41	10	3.27	2.89	anti-CTLA-4
**Kim 2024**	IFN-γ	5	25.27	7.60	5	13.41	11.75	anti-PD-1
**Kim J 2024**	CD8	7	0.95	0.07	6	0.99	0.08	anti-PD-L1
**Kuang 2022**	CD8	5	21.01	2.81	5	13.05	2.36	anti-PD-1
**Liu 2024**	CD8	3	24.18	4.57	3	11.29	4.03	anti-CTLA-4
**Lu 2024**	CD8	4	24.50	1.86	4	22.43	1.89	anti-PD-1
**Lu 2024**	CD8/Treg	4	1.84	0.16	4	1.43	0.13	anti-PD-1
**Meng 2022**	CD8	10	0.50	0.22	10	0.20	0.08	anti-PD-1
**Meng 2022**	IFN-γ	3	140.48	15.08	3	79.37	16.66	anti-PD-1
**Nakamura 2024**	CD8	5	33.89	76.33	5	8.89	18.27	anti-PD-L1
**Pan 2022**	CD8	4	0.68	0.26	4	0.46	0.06	anti-PD-1
**Qin 2024**	CD8	5	14.38	16.55	5	10.00	5.82	anti-PD-1
**Qin 2024**	PD-L1	5	29.53	14.29	5	42.75	21.69	anti-PD-1
**Reilley 2019**	CD8/Treg	8	15.96	9.42	8	4.97	3.40	anti-CTLA-4
**Reilley 2019**	CD8/Treg	8	7.85	3.92	8	4.97	3.40	anti-PD-1

Reilley 2019	Ki67	8	26.99	22.13	8	15.81	18.24	anti-CTLA-4
Reilley 2019	Ki67	8	25.53	24.80	8	15.81	18.24	anti-PD-1
Reyes 2021	CD8	12	1197.12	173.08	13	475.96	46.88	anti-PD-L1
Saida 2021	CD8	5	11.75	2.37	5	4.65	1.34	anti-CTLA-4+anti-PD-L1
Schetters 2020	CD8	10	24.70	13.78	10	3.20	3.68	anti-PD-1
Schetters 2020	CD8/Treg	10	2.42	2.05	10	1.00	1.62	anti-PD-1
Schwartz 2019	CD8	5	24.74	5.41	5	9.47	4.94	anti-CTLA-4
Schwartz 2019	IFN-γ	5	1.52	0.40	5	1.05	0.89	anti-CTLA-4
Sharma 2020	CD8	5	27.26	3.35	5	22.79	1.51	anti-PD-1
Takahashi 2025	CD8	15	1.07	0.66	15	1.01	0.66	anti-CTLA-4
Tomita 2018	CD8	6	0.66	0.27	6	0.46	0.17	anti-PD-1
Tomita 2018	CD8/Treg	6	4.13	3.03	6	3.29	1.68	anti-PD-1
Wang 2019	CD8	8	46.27	3.51	8	21.37	3.99	anti-PD-1
Wang 2019	CD8/Treg	8	2.47	0.31	8	1.00	0.26	anti-PD-1
Wu 2020	CD8	4	31.69	3.27	4	13.95	4.07	anti-PD-L1
Wu 2020	IFN-γ	4	19.94	2.60	4	19.10	8.92	anti-PD-L1
Xu 2024	CD8	5	4.53	1.72	5	2.35	1.33	anti-PD-1
Xu 2024	IFN-γ	5	2.97	1.73	5	1.84	1.23	anti-PD-1
Yang 2024	CD8	4	34.81	1.54	4	27.08	1.80	anti-PD-1
Yang 2024	CD8/Treg	4	3.41	0.50	4	0.96	0.14	anti-PD-1
Yang 2024	IFN-γ	4	38.53	7.47	4	14.53	1.58	anti-PD-1
Zhou 2022	IFN-γ	5	3.33	0.91	5	0.55	0.14	anti-PD-1
Zhu 2025	CD8	5	18.75	2.20	5	14.02	0.93	anti-PD-1
Zhu 2025	CD8/Treg	5	0.25	0.03	5	0.17	0.01	anti-PD-1
Zhu 2025	IFN-γ	5	1.09	0.26	5	0.45	0.10	anti-PD-1
Zhu 2025	Ki67	5	24.53	0.47	5	33.58	1.17	anti-PD-1

B. CLINICAL STUDIES

Study	Parameter	n_fav	mean_fav	sd_fav	n_unfav	mean_unfav	sd_unfav	Drug_Class	Comparator
**Blank 2018**	PD-L1	11	0.40	0.09	6	0.15	0.05	anti-CTLA-4+anti-PD-1	No relapse vs Relapse
**Daud 2016**	CD8	23	46.74	6.98	17	37.91	3.25	anti-PD-1	Responders vs Non-responders
**Daud 2016**	CD8/Treg	23	2.28	0.42	17	3.76	0.58	anti-PD-1	Responders vs Non-responders
**Edwards 2018**	CD8	5	1.84	1.50	5	0.34	0.27	anti-PD-1	Responders vs Non-responders
**Gide 2019**	CD8	22	132.23	132.23	19	49.59	77.13	anti-PD-1	Responders vs Non-responders
**Gide 2019**	CD8/Treg	22	2.21	2.74	19	1.38	2.64	anti-PD-1	Responders vs Non-responders
**Gide 2019**	PD-L1	22	1508.98	1748.51	19	191.62	574.85	anti-PD-1	Responders vs Non-responders
**Gide 2019**	CD8	24	137.74	126.72	8	49.59	77.13	anti-CTLA-4+anti-PD-1	Responders vs Non-responders
**Gide 2019**	CD8/Treg	24	3.41	4.86	8	0.83	1.81	anti-CTLA-4+anti-PD-1	Responders vs Non-responders
**Gide 2019**	PD-L1	24	2157.58	1866.66	8	945.45	1381.82	anti-CTLA-4+anti-PD-1	Responders vs Non-responders
**Girault 2022**	CD8	59	1.73	0.94	62	1.58	0.92	anti-CTLA-4+anti-PD-1	Responders vs Non-responders
**Girault 2022**	PD-L1	56	1.71	1.34	57	1.00	1.04	anti-CTLA-4+anti-PD-1	Responders vs Non-

									responders
**Huang 2011**	CD8	3	407.34	329.38	8	452.37	294.89	anti-CTLA-4	Responders vs Non-responders
**Huang 2011**	CD8/Treg	3	0.41	0.45	8	0.58	0.51	anti-CTLA-4	Responders vs Non-responders
**Huang 2011**	Ki67	3	266.23	137.72	8	130.27	162.73	anti-CTLA-4	Responders vs Non-responders
**Huang 2011**	CD8	19	955.00	832.55	19	289.00	265.89	anti-CTLA-4	Post-treatment vs Pre-treatment
**Huang 2011**	Ki67	19	835.12	515.23	19	1075.38	355.95	anti-CTLA-4	Post-treatment vs Pre-treatment
**Huang 2019**	CD8	9	1.80	1.25	9	0.34	0.55	anti-PD-1	Post-treatment vs Pre-treatment
**Huang 2019**	PD-L1	7	2.86	1.13	7	1.89	0.98	anti-PD-1	Post-treatment vs Pre-treatment
**Inoue 2016**	CD8/Treg	5	10.87	11.42	8	1.71	1.36	anti-PD-1	Responders vs Non-responders
**Inoue 2016**	PD-L1	5	0.00147	0.00098	8	0.000306	0.000297	anti-PD-1	Responders vs Non-responders
**Ji 2012**	CD8	9	144.80	65.10	36	52.60	65.10	anti-CTLA-4	Responders vs Non-responders
**Ji 2012**	IFN-g	9	14.40	4.24	36	9.50	4.24	anti-CTLA-4	Responders vs Non-responders
**Karapetyan 2025**	CD8	15	15.49	11.77	15	5.04	2.37	anti-PD-1	Post-treatment vs Pre-treatment
**Kasanen 2020**	CD8	6	0.00	0.11	3	0.22	0.89	anti-PD-1	Responders vs Non-

									responders
**Kluger 2018**	CD8	8	4.35	3.28	9	0.32	0.25	anti-PD-1	Responders vs Non-responders
**Kluger 2018**	PD-L1	8	14.56	12.91	9	4.71	1.46	anti-PD-1	Responders vs Non-responders
**Long 2025 (CheckMate 915)**	CD8	925	7.86	7.94	180	6.01	6.61	anti-PD-1	Responders vs Non-responders
**Long 2025 (CheckMate 915)**	IFN-g	748	0.05	0.88	145	-0.06	0.80	anti-PD-1	Responders vs Non-responders
**Long 2025 (CheckMate 915)**	PD-L1	919	0.46	0.66	179	0.42	0.66	anti-PD-1	Responders vs Non-responders
**Long 2025 (Morpheus)**	CD8	8	1814.70	1093.80	8	900.70	879.50	anti-CTLA-4+anti-PD-1	Post-treatment vs Pre-treatment
**Long 2025 (Morpheus)**	CD8/Treg	8	2.85	0.96	8	3.06	2.02	anti-CTLA-4+anti-PD-1	Post-treatment vs Pre-treatment
**Long 2025 (Morpheus)**	IFN-g	15	0.07	1.08	4	-0.52	1.03	anti-CTLA-4+anti-PD-1	Responders vs Non-responders
**Long 2025 (Morpheus)**	Ki67	8	985.80	777.10	8	411.50	405.50	anti-CTLA-4+anti-PD-1	Post-treatment vs Pre-treatment
**Rawson 2021**	CD8	23	24.80	13.10	6	42.80	26.80	anti-CTLA-4+anti-PD-1	Responders vs Non-responders
**Rawson 2021**	CD8/Treg	23	4.07	2.93	6	10.19	7.69	anti-CTLA-4+anti-PD-1	Responders vs Non-responders

**Rozeman 2023**	CD8	8	39.25	17.38	8	18.28	10.49	anti-PD-1	Post-treatment vs Pre-treatment
**Rozeman 2023**	IFN-g	8	1.49	2.02	8	0.00	0.04	anti-PD-1	Post-treatment vs Pre-treatment
**Sun 2024**	CD8	8	11.55	4.29	3	6.64	2.76	anti-PD-1	Responders vs Non-responders
**Sun 2024**	CD8/Treg	8	2.24	1.00	3	1.53	0.64	anti-PD-1	Responders vs Non-responders
**Tumeh 2014**	CD8	22	3.16	10.25	24	1.12	2.96	anti-PD-1	Responders vs Non-responders
**Tumeh 2014**	PD-L1	17	1.60	5.43	21	0.85	0.65	anti-PD-1	Responders vs Non-responders
**Vilain 2017**	CD8	9	416.00	452.00	10	332.00	452.00	anti-PD-1	Responders vs Non-responders
**Vilain 2017**	PD-L1	9	8.10	11.20	10	14.60	44.10	anti-PD-1	Responders vs Non-responders
**Vilain 2017**	Apoptosis	9	17.10	30.50	10	0.40	0.80	anti-PD-1	Responders vs Non-responders

Supplementary Table S3. Bubble plots for the meta-regression of drug class on each TME outcome. Each bubble corresponds to one data point, with size proportional to the study weight; horizontal lines denote subgroup mean effect estimates from the random-effects meta-regression.

A. PRECLINICAL STUDIES

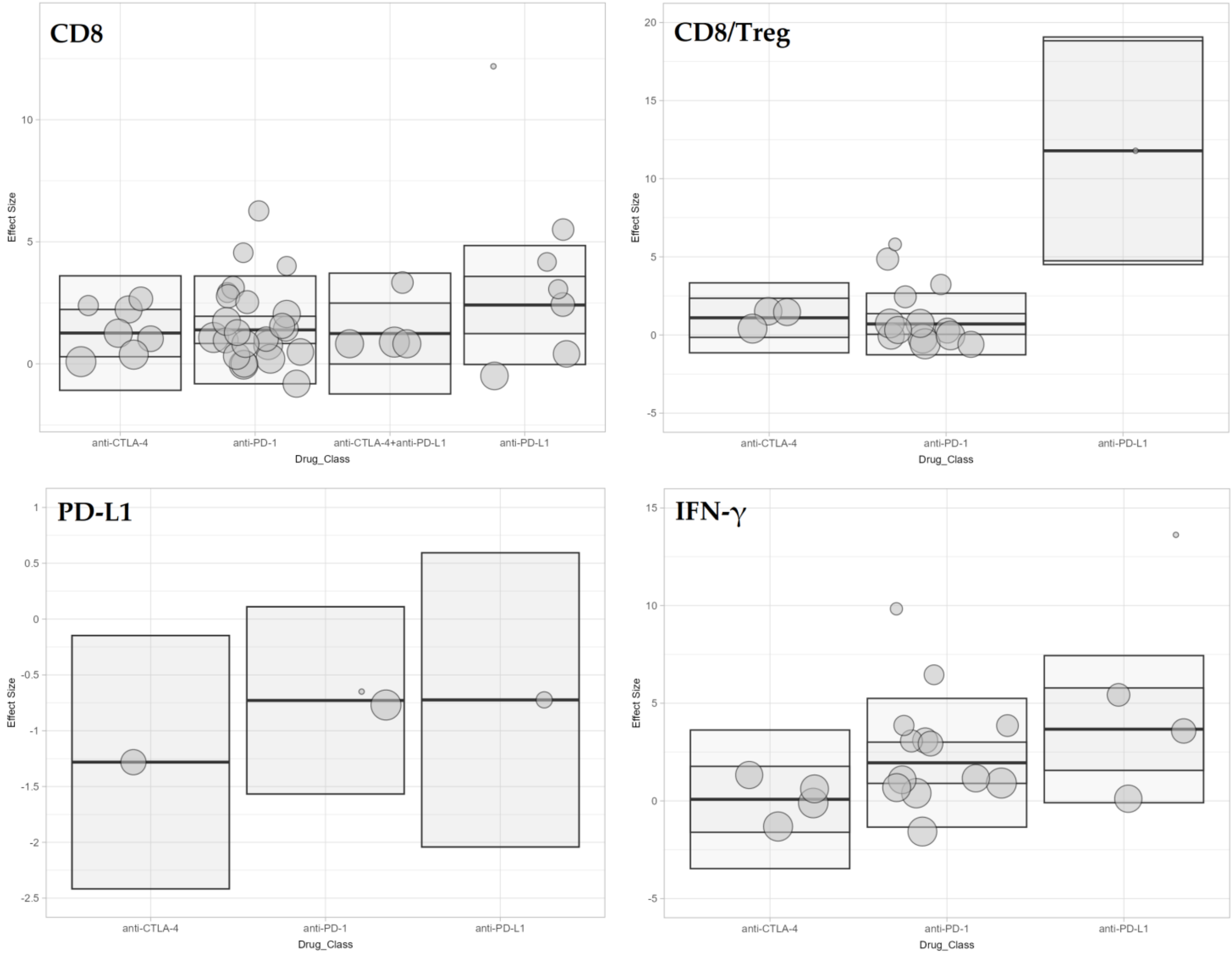

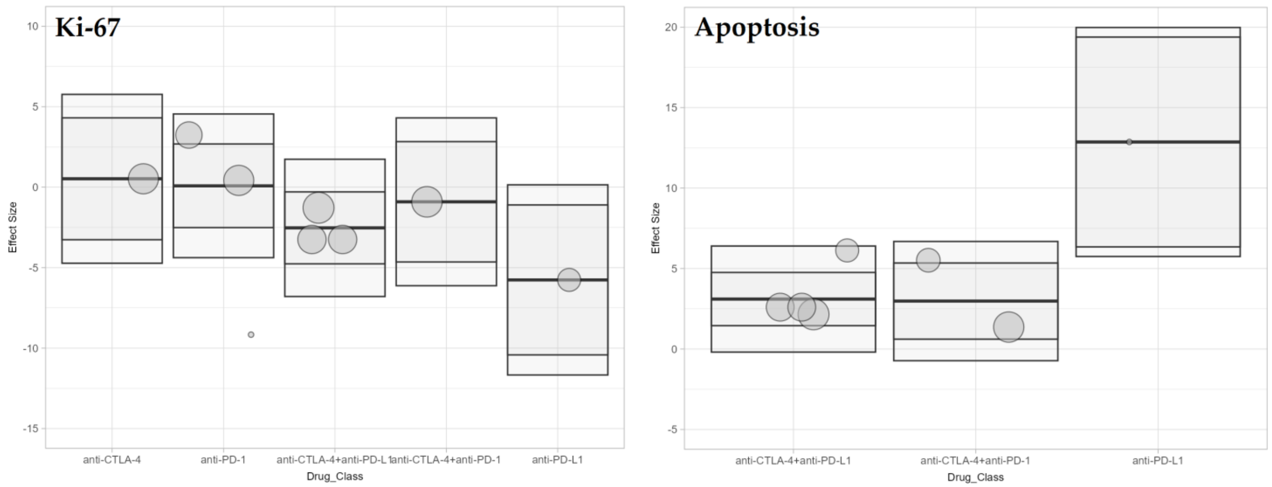

B. CLINICAL STUDIES

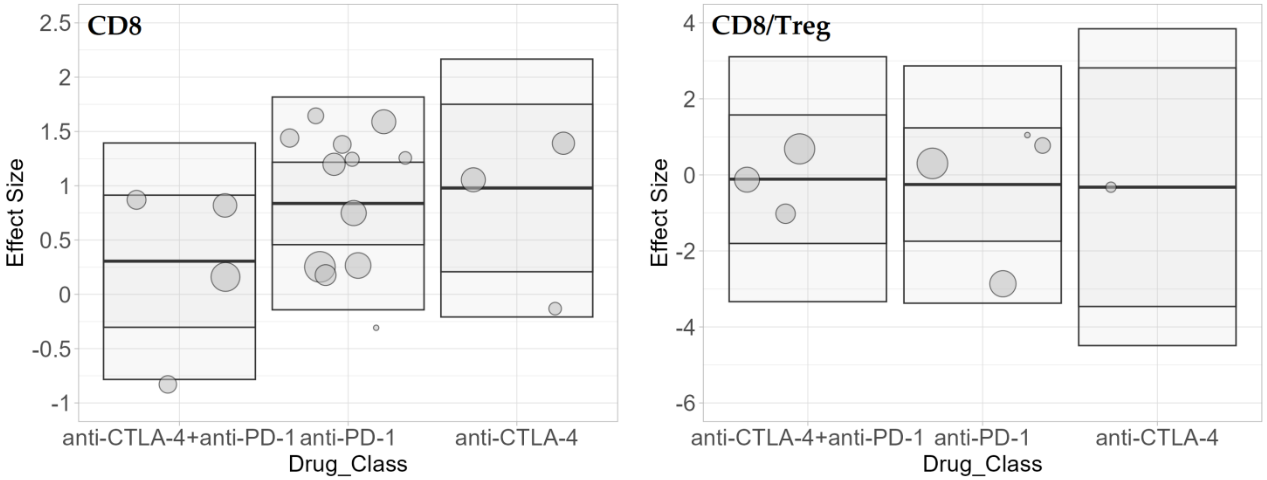

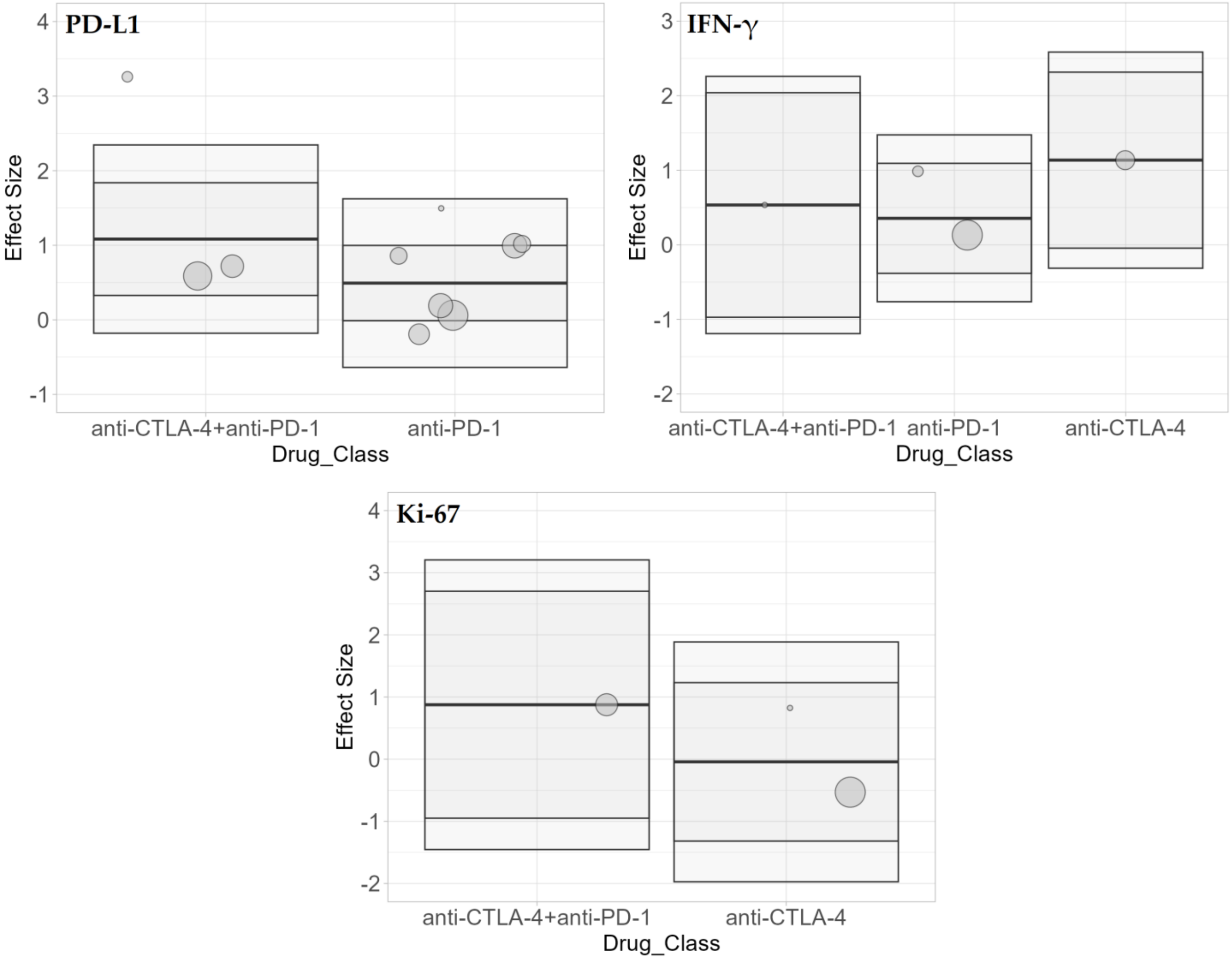

PD-L1
Effect Size
anti-CTLA-4+anti-PD-1
anti-PD-1
Drug_Class
IFN-γ
Effect Size
anti-CTLA-4+anti-PD-1
anti-PD-1
anti-CTLA-4
Drug_Class
Ki-67
Effect Size
anti-CTLA-4+anti-PD-1
anti-CTLA-4
Drug_Class

Supplementary Table S4. **Peripheral-only biomarker studies with no intratumoral TME data [132-179]**

A. PRECLINICAL STUDIES

First Author (Year)	Biomarker type	Key finding(s)
**Takahashi (2025)**	Tumor volume, flow cytometry (IFNγ$^+$CD8$^+$ T cells, Foxp3$^+$ Tregs), ELISA (IL-6, IFNβ)	SN38-PROTAC + anti-PD-1 increased intratumoral IFNγ$^+$CD8$^+$ T cells and CTL/Treg ratio in B16-F10 tumors, dependent on STING signaling. Numerical values not extractable.
**Liu (2018)**	Tumor volume, flow cytometry (CD8$^+$, CD4$^+$ T cells, PD-1, PD-L1), ELISA (IFN-γ)	Anti-PD-1, anti-PD-L1, and anti-CTLA-4 monotherapy delayed B16 tumor growth and increased CD8$^+$ T cell infiltration and IFN-γ production. Numerical values not extractable.
**Rouanet (2020)**	Tumor volume, survival, RT-qPCR (Ctla4, Pdcd1lg2, Ifnγ, Tnfα, Gzmb)	Anti-PD-1 monotherapy showed best single-agent survival benefit; anti-CTLA-4 + anti-PD-1 combination most effective in B16F10 model. Numerical intratumoral TME data not reported.

B. CLINICAL STUDIES

First Author (Year)	Biomarker type	Key finding(s)
**Arakawa (2019)**	T-cell receptor repertoire	Restricted CD4+ TCR diversity pre-treatment predicts longer survival; increasing CD8+ restriction post-treatment associated with worse survival and severe irAEs.
**Babačić (2020)**	Plasma proteins (PEA, LC-MS)	Increase in IL-6, IL-10, CCL2, CCL3, CCL4, sPD-1, sPD-L1 during anti-PD-1 therapy associated with response; high baseline levels negative.
**Bolovan (2025)**	Flow cytometry (PBMC), serum cytokines	Increasing CD8+ T cells and NK cells during nivolumab; IL-6 negatively correlated with T cell subsets; LDH correlated with IL-6 and S100.
**Bustos (2020)**	Cell-free microRNAs (cfmiRs)	29-cfmiR signature in plasma distinguishes melanoma brain metastasis from other brain tumors.
**Chen (2025)**	Plasma cytokines, CBC	Baseline IL-23, CXCL6, IL-10 predict durable clinical benefit (AUC=0.79); early decrease in NLR (driven by lymphocyte increase) predicts better survival.
**Comin-Anduix (2008)**	Flow cytometry (PBMC), MHC tetramers	No sustained increase in melanoma-specific CD8+ T cells; increase in T-cell activation/memory markers (HLA-DR, CD45RO) correlates with response.
**Comin-Anduix (2010)**	Phosphoflow (PBMC)	Tremelimumab alters TCR/cytokine signaling in T cells and monocytes; differential phosphorylation of pZAP70, pAkt, pSTAT6 associated with response.
**Cristinziano (2022)**	Flow cytometry (neutrophils)	High baseline frequency of PD-L1+ neutrophils (≥44.9%) associated with worse OS and PFS, especially in BRAF wild-type patients.
**Dalle (2024)**	Flow cytometry (whole	Baseline immune dysfunction (reduced NK function, altered T-cell phenotype); on-treatment

	blood)	decrease in HVEM on T cells and increased memory T cell polyfunctionality in responders.
**Edwards (2025)**	Spectral flow cytometry (PBMC)	On-treatment expansion of CD4+ central memory T cells (Ki67+) predicts response; higher TIM-3+ CD8+ T cells associated with severe irAEs.
**Friedlander (2017)**	Whole-blood RNA (qPCR)	15-gene classifier (including TGFB1) predicts response and 1-year survival to tremelimumab (AUC 0.86 discovery, 0.62 validation).
**Gambichler (2019)**	Flow cytometry (PBMC)	Adjuvant nivolumab decreases circulating PD-1+ Tregs and increases CTLA-4+ Tregs; no change in CD8+ or CD4+ T cell counts.
**Grecea-Balaj (2025)**	Flow cytometry, serum cytokines	Stable CD8+ T cell percentages; elevated IL-2 at 3 months and TNF-α at 6 months correlate with longer OS in melanoma.
**Hurkmans (2020)**	Serum ECM biomarkers (ELISA)	High baseline PRO-C3 (collagen formation) associated with worse PFS/OS; on-treatment increase in VICM (M1 macrophage activity) predicts better OS.
**Khattak (2020) (CTC)**	Flow cytometry (CTCs)	Presence of ≥1 PD-L1+ circulating tumour cell at baseline predicts response to pembrolizumab (median PFS 26.6 vs 5.5 months).
**Khattak (2020) (VEGF)**	Serum VEGF (Luminex)	High baseline serum VEGF associated with poor response and worse PFS specifically in ipilimumab-treated patients.
**Koguchi (2015)**	Serum cytokines (Luminex)	High baseline CXCL11 and sMICA predict poor OS after ipilimumab; elevated VEGF, LDH, sCD25 and low ALC are general prognostic factors.
**Koguchi (2021)**	Serum drug levels, cytokines	Higher ipilimumab trough levels associated with better OS and irAEs; inversely correlated with baseline CXCL11, sCD25 and on-treatment CRP, IL-6.
**Krieg (2018)**	Mass cytometry (PBMC)	High baseline frequency of activated classical monocytes (>19.38%) predicts better PFS/OS; responders have lower circulating CD8+ T cells but with activated phenotype.

**Lee (2017)**	ctDNA (ddPCR)	Persistently detectable ctDNA identifies patients with very poor prognosis (ORR 6%, median PFS 2.7 mo); ctDNA dynamics superior to LDH.
**Lereim (2025)**	Plasma cytokines (multiplex)	Baseline G-CSF, IL-2RA, MIP-1α, SCF higher in non-responders; high IL-2RA, IFNγ, PDGF-bb, MIG associated with inferior OS.
**Lim (2019)**	Plasma cytokines (65-plex)	CYTOX score (11 cytokines) predicts severe irAEs in anti-PD-1 + anti-CTLA-4 treated patients (AUC 0.68-0.78).
**Martinović (2023)**	Serum cytokines (ELISA)	Distinct cytokine dynamics (TGF-β, IL-6, IFN-γ, IL-8, IL-10) during pembrolizumab associated with disease control vs progression.
**Martinovic (2025)**	Flow cytometry (PBMC), CBC	Responders show increased NK cell degranulation (CD107a), activating receptors (NKG2D, NKp46), decreased CD14+HLA-DR- monocytes, and lower NLR.
**Nyakas (2019)**	Serum proteins (ELISA)	High baseline endostatin and galectin-3 binding protein (Gal3BP) are independent prognostic biomarkers for poor 2-year survival after ipilimumab.
**Pedersen (2020)**	ctDNA (ddPCR), plasma cytokines	Undetectable ctDNA at week 6-8 predicts longer PFS (26.3 vs 2.1 mo); high baseline MCP-1 and TNF-α associated with superior PFS.
**Pedersen (2022)**	Plasma cytokines, soluble checkpoints	High increment of sPD-1 predicts superior PFS in nivolumab+ipilimumab (HR=0.13); treatment-specific cytokine signatures differ between nivo/ipi and pembro.
**Pico de Coaña (2020)**	Flow cytometry (PBMC), CBC	High CD69+ NK cells and monocytic MDSCs predict shorter PFS/OS; high non-classical monocytes predict better outcome; NLR and LDH negative.
**Rad Pour (2021)**	scRNA-seq, flow cytometry (monocytes)	High frequency of S100A9+ monocytes (>15.3%) associated with poor response and shorter survival; higher CD4+ T cell/monocyte ratio predicts better response.
**Reuben (2006)**	Flow cytometry (PBMC), PCR,	Responders show decreased circulating Tregs, decreased constitutive IL-10, and increased activation-induced IL-2; distinct CTLA-4/GITR vs CTLA-4/PD-1 correlations.

	cytokines	
**Romano (2017)**	Flow cytometry (PBMC)	Low frequency of FKBP51s+ Tregs predicts non-response to ipilimumab (92.6% of low expressors were NR); also predicts response to subsequent nivolumab.
**Sosa Cuevas (2024)**	Multi-parametric flow cytometry (PBMC)	Responders have higher baseline cDC1s, CD8+ T cells, PD-1+ CD4+ T cells, and distinct γδ T-cell differentiation profiles; non-responders show increased PD-L2 on DCs.
**Sun (2021)**	Flow cytometry (MDSC subsets)	Total MDSC increases on therapy; responders show sharp rise and plateau, non-responders have higher baseline and blunted increase; double-positive CD14+CD15+ MDSC increase in PD.
**Takahashi (2020)**	Flow cytometry (intracellular PD-1)	Intracellular PD-1 accumulation in CD8+ T cells; nivolumab enhances antigen-specific IFN-γ production; intracellular PD-1 may be a reservoir for surface PD-1.
**Tietze (2017)**	Flow cytometry (PBMC)	Low baseline CD45RO+ CD8+ memory T cells (≤25%) associated with non-response to ipilimumab (80% of NR); effector memory subset drives correlation.
**Triozzi (2022)**	scRNA-seq, flow cytometry, metabolomics	Responder T cells have higher glycolytic capacity, spare respiratory capacity, and SLC2A14 (Glut-14) expression; plasma metabolomics distinguishes responders.
**Visconti (2023)**	Serum N-glycome (UHPLC)	Pre-treatment N-glycan signature (low antennary fucose, high low-branched glycans) associated with response and longer survival.
**Wang (2020)**	Serum cytokines (Luminex), machine learning	16-cytokine signature predicts nivolumab clearance and overall survival (AUC=0.75), validated in independent cohort.
**Weber (2012)**	Flow cytometry (PBMC), ELISA	Ipilimumab increases activated and central/effector memory CD8+ T cells, decreases naive T cells; no change in Treg frequency; boosts humoral responses to tumour/recall antigens.

**Willsmore (2025)**	Mass cytometry (B cells), serum antibodies	Expanded regulatory B cells (IL-10+ plasmablasts, DN B cells) and Th2-biased antibodies (IgG4, IgE) at baseline predict protection from irAEs; early B-Cell decrease (≥13%) predicts worse survival.
**Wistuba-Hamprecht (2017)**	Flow cytometry (T-cell subsets)	High baseline CD8 EM1 (>13%) associated with longer OS and higher response; decrease in PD-1+EM1 during ipilimumab associated with response.
**Woods (2018)**	Flow cytometry (Treg pSTAT3), in vitro assays	Increased Treg pSTAT3 and reduced Treg suppressive function in patients with clinical benefit; PD-1 blockade induces IL-10 from CD8+ T cells in STAT3-dependent manner.
**Woods (2020)**	High-parameter flow cytometry (PBMC)	NIVO and IPI induce distinct immunophenotypic changes; CD4+CD38+CD39+CD127−GARP− T cells increase after IPI and associate with resistance to subsequent NIVO.
**Wu (2018)**	ELISA, Luminex	Anti-CTLA-4 (but not anti-PD-1) elicits anti-Gal-3 antibodies; high baseline Gal-3 and Gal-1 predict poor response to PD-1 blockade.
**Yang (2025)**	Serum proteomics (Olink)	10-protein IFNγ-associated signature (including CCL3, CCL4, CCL7, LAG3) predicts major pathological response (AUC=0.68) in neoadjuvant setting.

Supplementary Table S5. **Full GRADE Evidence Profile for All Primary Outcomes.**

Certainty assessment							№ of patients		Effect		Certainty	Importance
№ of studies	Study design	Risk of bias	Inconsistency	Indirectness	Imprecision	Other considerations	Immune checkpoint inhibitors	Control, comparator	Relative (95% CI)	Absolute (95% CI)		
**CD8+ T-cell infiltration (Preclinical)**												
41	non-randomised studies	serious[a]	not serious	not serious	not serious	strong association, all plausible residual confounding would reduce the demonstrated effect[i,m]	270	259	-	SMD 1.45 SD higher (1.05 higher to 1.85 higher)	⊕⊕⊕◯ Moderate[a,i,m]	CRITICAL
**CD8/Treg ratio (Preclinical)**												
17	non-randomised studies	serious[a]	not serious	not serious	not serious	none	85	85	-	SMD 0.91 SD higher (0.28 higher to 1.55 higher)	⊕⊕◯◯ Low	CRITICAL

Certainty assessment							№ of patients		Effect		Certainty	Importance
№ of studies	Study design	Risk of bias	Inconsistency	Indirectness	Imprecision	Other considerations	Immune checkpoint inhibitors	Control, comparator	Relative (95% CI)	Absolute (95% CI)		
**PD-L1 expression (Preclinical)**												
4	non-randomised studies	serious[a]	not serious	not serious	serious[c]	none	30	30	-	SMD 0.88 SD lower (1.48 lower to 0.28 lower)	⊕◯◯◯ Very low[a,c]	CRITICAL
**IFN-γ production (Preclinical)**												
21	non-randomised studies	serious[a]	not serious	not serious	not serious	strong association, all plausible residual confounding would reduce the demonstrated effect[j,m]	107	107	-	SMD 1.78 SD higher (0.95 higher to 2.62 higher)	⊕⊕⊕◯ Moderate[a,j,m]	CRITICAL
**Ki-67 proliferation (Preclinical)**												
9	non-randomised studies	serious[a]	not serious	not serious	not serious	strong association[k]	63	67	-	SMD 1.43 SD lower (2.7 lower to 0.15 lower)	⊕⊕◯◯ Low[a,k]	CRITICAL

Certainty assessment							№ of patients		Effect		Certainty	Importance
№ of studies	Study design	Risk of bias	Inconsistency	Indirectness	Imprecision	Other considerations	Immune checkpoint inhibitors	Control, comparator	Relative (95% CI)	Absolute (95% CI)		
**Apoptosis (Preclinical)**												
7	non-randomised studies	serious[a]	not serious	not serious	not serious	very strong association[l]	56	60	-	SMD 3.54 SD higher (2.13 higher to 4.95 higher)	⊕⊕⊕◯ Moderate[a,l]	CRITICAL
**CD8+ T-cell infiltration (Clinical)**												
19	RCTs & non-randomised studies	serious[e]	not serious	not serious	not serious	all plausible residual confounding would reduce the de-monstrated effect[n]	1205	449	-	SMD 0.72 SD higher (0.44 higher to 1.01 higher)	⊕⊕◯◯ Low[e,n]	CRITICAL

Certainty assessment							№ of patients		Effect		Certainty	Importance
№ of studies	Study design	Risk of bias	Inconsistency	Indirectness	Imprecision	Other considerations	Immune checkpoint inhibitors	Control, comparator	Relative (95% CI)	Absolute (95% CI)		
**CD8/Treg ratio (Clinical)**												
8	RCTs & non-randomised studies	serious[e]	not serious	not serious	not serious	none	116	77	-	SMD 0.21 SD lower (1.12 lower to 0.7 higher)	⊕⊕◯◯ Low[e]	CRITICAL
**PD-L1 expression (Clinical)**												
10	RCTs & non-randomised studies	serious[e]	not serious	not serious	not serious	all plausible residual confounding would reduce the de monstrated effect[n]	1078	324	-	SMD 0.67 SD higher (0.26 higher to 1.08 higher)	⊕⊕◯◯ Low[e,n]	CRITICAL
**IFN-γ production (Clinical)**												
4	RCTs & non-randomised studies	serious[e]	not serious	not serious	not serious	none	780	193	-	SMD 0.59 SD higher (0.03 lower to 1.2 higher)	⊕⊕◯◯ Low[e]	CRITICAL

Certainty assessment							№ of patients		Effect		Certainty	Importance
№ of studies	Study design	Risk of bias	Inconsistency	Indirectness	Imprecision	Other considerations	Immune checkpoint inhibitors	Control, comparator	Relative (95% CI)	Absolute (95% CI)		
**Ki-67 proliferation (Clinical)**												
3	RCTs & non-randomised studies	serious[e]	not serious	not serious	serious[h]	none	30	35	-	SMD 0.26 SD higher (0.8 lower to 1.32 higher)	⨁◯◯◯ Very low[e,h]	CRITICAL

CI: confidence interval; **SMD:** standardized mean difference

Question: Immune checkpoint inhibitors (anti-CTLA-4, anti-PD-1, anti-PD-L1, monotherapy or combination) compared to placebo, untreated control, active comparator, or standard of care for preclinical B16F10/C57BL/6 melanoma models and clinical cutaneous melanoma cohorts

Explanations:

a. Risk of bias (Preclinical): Across all preclinical outcomes, 54 of 58 included studies (93%) were rated as having "some concerns" on the SYRCLE tool, primarily due to incomplete reporting of randomization, allocation concealment, and blinding. This constitutes a serious limitation, warranting a one-level reduction in certainty.

b. Imprecision (Preclinical CD8/Treg): Based on 17 studies. The 95% confidence interval (0.28 to 1.55) is wide and crosses thresholds for both small and large effects, despite statistical significance (p=0.005). Certainty reduced by one level.

c. Imprecision (Preclinical PD-L1): Based on only 4 studies with approximately 40 animals total. The 95% confidence interval (-1.48 to -0.28) is wide, and the optimal information size was not met. Certainty reduced by one level.

d. Imprecision (Preclinical Ki-67): Based on only 9 studies. The 95% confidence interval (-2.70 to -0.15) is wide, and the optimal information size was not met. Certainty reduced by one level.
e. Risk of bias (Clinical): Across all clinical outcomes, 19 studies were assessed with RoB 2 and ROBINS-I. One RCT had high risk, and 33% of non-randomized studies had serious risk of bias. This constitutes a serious limitation, warranting a one-level reduction in certainty.
f. Imprecision (Clinical CD8/Treg): Based on 8 studies. The 95% confidence interval (-1.12 to 0.70) is wide and crosses the null. Certainty reduced by one level.
g. Imprecision (Clinical IFN-γ): Based on only 4 studies. The 95% confidence interval (-0.03 to 1.20) crosses the null, and the optimal information size was not met. Certainty reduced by one level.
h. Imprecision (Clinical Ki-67): Based on only 3 studies with small total sample size. The 95% confidence interval (-0.80 to 1.32) is very wide and crosses the null. Certainty reduced by one level.

Factors Increasing Certainty
i. Large magnitude of effect (Preclinical $CD8^+$): The pooled standardized mean difference exceeds 1.0 (SMD = 1.45), representing a substantial increase in $CD8^+$ T-cell infiltration. According to GRADE, a large effect increases confidence in the finding, warranting a one-level increase in certainty.
j. Large magnitude of effect (Preclinical IFN-γ): The pooled standardized mean difference exceeds 1.0 (SMD = 1.78), representing a large increase in IFN-γ production. A one-level increase in certainty was applied.
k. Large magnitude of effect (Preclinical Ki-67): The absolute magnitude of the pooled standardized mean difference exceeds 1.0 (SMD = –1.43), representing a substantial reduction in tumor cell proliferation. A one-level increase in certainty was applied.
l. Very large magnitude of effect (Preclinical Apoptosis): The pooled standardized mean difference exceeds 2.0 (SMD = 3.54), representing an exceptionally large induction of apoptosis. GRADE guidance permits a two-level increase in certainty for very large effects.
m. Plausible residual confounding (Preclinical $CD8^+$, IFN-γ): In highly controlled preclinical models, any unmeasured sources of variability (e.g., subtle differences in tumor implantation or environmental factors) would be expected to introduce noise that underestimates the true treatment effect. The observation of robust, statistically significant effects despite this inherent variability increases confidence in the findings, warranting a one-level increase in certainty.
n. Plausible residual confounding (Clinical $CD8^+$, PD-L1): In the included clinical studies, patients with higher disease burden or poorer prognosis were more likely to receive immunotherapy. This confounding by indication would, if anything, underestimate the true benefit of treatment. The clear effects observed despite this bias increase confidence in the findings, warranting a one-level increase in certainty.
